# Mechanism of Contrasting Ionic Conductivities in Li_2_ZrCl_6_ via I and Br Substitution

**DOI:** 10.1002/smll.202505926

**Published:** 2025-09-02

**Authors:** Yeji Choi, Hiram Kwak, Jae‐Seung Kim, Daseul Han, Hae‐Yong Kim, Sunho Ko, Jong Seok Kim, Juhyoun Park, Gihan Kwon, Kyung‐Wan Nam, Dong‐Hwa Seo, Yoon Seok Jung

**Affiliations:** ^1^ School of Chemical and Biomolecular Engineering Yonsei University Seoul 03722 Republic of Korea; ^2^ Department of Battery Engineering Yonsei University Seoul 03722 Republic of Korea; ^3^ Department of Materials Science and Engineering Korea Advanced Institute of Science and Technology (KAIST) Daejeon 34141 Republic of Korea; ^4^ Department of Energy and Materials Engineering Dongguk University Seoul 04620 Republic of Korea; ^5^ National Synchrotron Light Source II Brookhaven National Laboratory Upton NY 11973 USA

**Keywords:** anion substitutions, halide solid electrolytes, Li^+^ ionic conductivities, solid‐state batteries, structural disorders

## Abstract

Understanding the complex structural and chemical factors that influence ionic conduction mechanisms is paramount for developing advanced inorganic superionic conductors in all‐solid‐state batteries, particularly halide solid electrolytes with excellent electrochemical oxidative stability and mechanical sinterability. Herein, contrasting ionic conduction behaviors in I^−^ and Br^−^ substituted Li_2_ZrCl_6_ are revealed by combining experimental structural analyses and theoretical calculations. The inter‐slab distance along the *c*‐axis, which varies with the anion substitution and M2‐M3 site disorder, is a key factor for opening the *ab*‐plane conduction and facilitating the overall Li^+^ conduction. Increased M3 site occupancy generally leads to contracted inter‐slab distance. The substantial increase in Li^+^ conductivity upon I substitution (from 0.40 to 0.91 mS cm^−1^) originates from a sufficiently expanded lattice volume owing to its large ionic radii (I^−^ = 2.20 Å), particularly inter‐slab distance that facilitates the *ab* intra‐plane Li^+^ conduction, which also benefits from decreased M2‐M3 disorder. In contrast, Br (Br^−^ = 1.96 Å) substitution results in insufficiently expanded Li^+^ channels, which, exacerbated by increased M2‐M3 disorder, leads to degradation in Li^+^ conductivity. Implementing I^−^ substituted Li_2_ZrCl_6_ resulted in superior electrochemical performance in LiCoO_2_||Li‐In cells compared to those with an unsubstituted catholyte.

## Graphical abstract

Iodine and bromine substitutions in Li_2_ZrCl_6_ distinctly influence Li^+^ conductivity through changes in inter‐slab distance and cation site disorder. Iodine enlarges conduction pathways and reduces disorder, enhancing ionic transport, whereas bromine induces structural constraints and increases disorder, suppressing conductivity. These findings guide the structural design of halide solid electrolytes for high‐performance all‐solid‐state batteries.

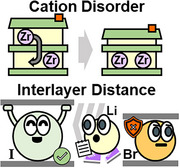

## 1 Introduction

While lithium‐ion batteries have become indispensable in daily life applications, they have limitations in terms of energy density and pose safety risks,^[^ ^1^, ^2^ ^]^ consequently spurring extensive research into all‐solid‐state batteries (ASSBs), which rely on the innovation of advanced inorganic solid electrolytes (SEs).^[^ ^2^, ^3^, ^4^, ^5^, ^6^, ^7^, ^8^, ^9^, ^10^, ^11^, ^12^, ^13^, ^14^, ^15^ ^]^ In particular, the investigation into the factors affecting ionic conduction mechanisms in inorganic SEs is a vibrant and evolving field, with the structure and composition of the anion framework being key considerations^.[^ ^7^, ^8^, ^9^, ^10^, ^13^, ^16^, ^17^, ^18^, ^19^, ^20^, ^21^ ^]^ For a class of identical anions, the body‐centered cubic (bcc) anion framework is recognized for its lower activation barrier compared to hexagonal close‐packed (hcp) and cubic close‐packed (ccp) structures.^[^ ^17^ ^]^ However, several compounds based on close‐packed structures exhibit superionic conduction,^[^ ^22^, ^23^, ^24^, ^25^, ^26^, ^27^, ^28^, ^29^, ^30^, ^31^, ^32^ ^]^ explainable by the concerted migration of charge‐carrier cations.^[^ ^21^ ^]^ When comparing structures with different anions (e.g., S^2−^ vs O^2−^), the introduction of larger and more polarizable anions generally enhances ionic conduction, attributed to the increased lattice size, weaker binding to Li^+^ owing to the lower electronegativity of S^2−^, and lower activation energy (E_A_).^[^ ^16^, ^33^ ^]^ In particular, the dynamic structural evolution of argyrodite‐type sulfide SEs (Li_7‐y_PS_6‐y_X_y_ (X═Cl, Br, I, 0 ≤ y < 2)) that affect Li^+^ conductivity illustrates the importance and complexity of anion compositional tuning. Specifically, substituting Cl^−^ with larger and more polarizable Br^−^ or I^−^ generally lowers both the activation energy and the Arrhenius pre‐factor due to lattice softening. However, in the case of I^−^ substitution, the lack of S^2−^/X^−^ site disorder leads to an increase in activation energy, while the pre‐factor either rises again or remains unchanged, thereby complicating the overall conductivity trend.^[^ ^34^, ^35^, ^36^ ^]^ Moreover, when I is partly or wholly used in X in Li_6_PS_5_X, beneficial S^2−^/X^−^ site disorder does not occur due to the significant size mismatch between I^−^ and X^−^ ions, thereby increasing the Li^+^ migration activation energy and ultimately lowering the overall ionic conductivity.^[^ ^35^, ^36^, ^37^ ^]^ The S^2−^/X^−^ site disorder can also be achieved through cation P‐site substitution.^[^ ^36^, ^38^, ^39^ ^]^ Consequently, state‐of‐the‐art argyrodite SEs (e.g., Li_6+x_P_1‐x_Ge_x_S_5_I, Li_6+x_M_x_Sb_1‐x_S_5_I (M═Si, Ge, Sn), Li_6‐x_PS_5‐x_ClBr_x_) have achieved ionic conductivities of ≥10 mS cm^−1^.^[^ ^36^, ^38^, ^39^, ^40^, ^41^, ^42^ ^]^

Since the landmark report on Li_3_YCl_6_ and Li_3_YBr_6_ in 2018,^[^ ^22^ ^]^ halide SEs have been considered promising candidates owing to their excellent electrochemical oxidative stability, mechanical deformability, and high ionic conductivity.^[^ ^7^, ^8^, ^9^, ^13^, ^22^, ^25^, ^26^, ^27^, ^43^, ^44^, ^45^, ^46^ ^]^ Extensive investigations have identified novel compositions with high ionic conductivities of ≥1 mS cm^−1^, which can be categorized into several classes based on the anion framework; i) close‐packed structure (e.g., Li_3_YCl_6_, Li_3_YBr_6_, Li_3_InCl_6_, Li_x_ScCl_3+x_, Li_2_ZrCl_6_, Li_3_YbCl_6_,),^[^ ^22^, ^23^, ^24^, ^25^, ^26^, ^27^, ^28^ ^]^ ii) non‐close‐packed structure (e.g., LiMOCl_4_ (M═Nb, Ta), Li_0.388_Ta_0.238_La_0.475_Cl_3_, LiTaCl_5_X^n−^_1/n_ (X═F, Cl, Br, I, O_2_, OH, O, S)),^[^ ^47^, ^48^, ^49^, ^50^ ^]^ and iii) others, including pliable SEs, such as xLiCl‐GaF_3_ and LiAlCl_4‐2x_O_x_.^[^ ^51^, ^52^, ^53^, ^54^ ^]^ When paired with uncoated layered Li_x_MO_2_ (M═Co, Ni, Mn, and/or Al mixture) cathode active materials (CAMs), halide SEs have demonstrated outstanding durability in ASSB cells.^[^ ^25^, ^26^, ^27^, ^30^, ^31^, ^47^, ^48^, ^49^, ^50^, ^53^, ^55^, ^56^, ^57^, ^58^, ^59^, ^60^ ^]^ The applicability of halide SEs has been extended to Li‐excess layered oxide CAMs.^[^ ^61^, ^62^, ^63^, ^64^ ^]^ Despite their close‐packed anion frameworks, many halide SEs exhibit high ionic conductivities, attributable to the low Coulombic attraction between the monovalent halogen anions and Li^+^.^[^ ^19^ ^]^ Therefore, the ionic conduction mechanisms of halide SEs have become a central focus in the ASSB field.^[^ ^19^, ^22^, ^24^, ^26^, ^27^, ^30^, ^47^, ^50^, ^65^, ^66^, ^67^, ^68^ ^]^

The initially investigated Li_3_YCl_6_ and Li_3_YBr_6_ adopt different hcp and ccp structures, respectively, which affect the Li^+^ pathways. Li_3_YCl_6_ (hcp) features a 1D Li^+^ network along the *c*‐axis, connected by tetrahedral sites in the *ab* plane, creating an overall anisotropic pathway. In contrast, Li_3_YBr_6_ (ccp) forms an isotropic 3D Li^+^ pathway.^[^ ^19^ ^]^ For hcp Li_3_YCl_6_, the Li^+^ conduction along the *c*‐axis is influenced by the position of the central metal. Mechanochemical methods induce cation disorder, where the central metal shifts from the (002) plane (i.e., M2 sites) to the (001) plane (i.e., M3 sites)—referred to as “M2‐M3 disorder.” This shift activates the connection between the 2D Li^+^ pathway in the *ab* plane and the 1D pathway along the *c*‐axis, thereby enhancing ionic conductivity.^[^ ^24^, ^65^ ^]^ Stacking faults or defects between the slabs have a similar effect on ionic conductivity as M2‐M3 disorder, resulting in a locally sparse distribution of Y^3+^.^[^ ^67^ ^]^ This change in the cation arrangement alters the repulsive forces between Y^3+^ and Li^+^, thereby influencing the ionic conductivity.^[^ ^67^ ^]^ Yu et al. recently investigated the effects of the central metal distribution on ion conduction mechanisms and analyzed the structural properties along the *ab* plane and *c*‐axis directions through theoretical calculations for each possible structure.^[^ ^68^ ^]^ They discovered that when the central metal is absent from the (002) plane, the M2‐M3 disorder shortens the inter‐slab distance, disrupting the Li^+^ pathway in the *ab* plane. Furthermore, even without M2‐M3 disorder, the YCl_6_^3−^ polyhedra in the *ab* plane can obstruct the Li^+^ pathway in the (001) plane, yielding an optimal Y occupancy in the (002) plane, ranging from 0.167 to 0.444.^[^ ^68^ ^]^

Li_2_ZrCl_6_ (LZC) is an important halide SE because Zr is neither scarce nor expensive, unlike many other halide SEs.^[^ ^13^, ^26^, ^31^, ^69^ ^]^ Depending on the preparation method, LZC can adopt either hcp or ccp structures. Heat treatment of LZC results in a monoclinic ccp structure with a low Li^+^ conductivity of 5.7 × 10^−6^ S cm^−1^ at 30 °C. In contrast, the mechanochemical method yields a trigonal hcp structure with a higher Li^+^ conductivity of 0.40 mS cm^−1^.^[^ ^26^ ^]^ A distinct difference from previous studies on other halide SEs with trivalent central metals such as Y is the tetravalence of Zr, making the investigation particularly intriguing. A recent study using Monte Carlo molecular dynamics simulations proposed that the disorder in LZC affects the Li^+^ conduction mechanisms.^[^ ^70^ ^]^ However, a comprehensive structural understanding and detailed ion conduction mechanism for LZC, integrating both experimental and theoretical insights, remain limited.

In this study, we systematically investigated the structural evolution of a series of Li_2_ZrCl_6‐y_X_y_ (X═Br or I) compounds and their effects on Li^+^ conduction dynamics. The I substitution increased Li^+^ conductivity, reaching a maximum of 0.91 mS cm^−1^ at 30 °C for Li_2_ZrCl_5.5_I_0.5_, showcasing more than a twofold improvement over LZC (0.40 mS cm^−1^). In sharp contrast, Br substitution hardly increased the Li^+^ conductivity. Complementary analyses using X‐ray diffraction (XRD), pair distribution function (PDF), and X‐ray absorption spectroscopy (XAS) measurements revealed structural evolutions varying with M2‐M3 disorder and anion substitutions, affecting Li^+^ transport. These findings were successfully supported by density functional theory (DFT) calculations with ab initio molecular dynamics (AIMD) simulations. Contrary to previous observations in Li_3_YCl_6_ and Li_3_ErCl_6_,^[^ ^24^, ^65^ ^]^ in Li_2_ZrCl_6_, the structure with a fully occupied M2 site (M2F, i.e., 100% M2 site occupancy at the (002) plane) exhibited higher Li^+^ diffusivity than the structure with a fully occupied M3 site (M3F, i.e., 100% M3 site occupancy at the (001) plane), rationalized by the more expanded inter‐slab spacing. While Br^−^ substitution increases Zr M2‐M3 site disorder, I^−^ substitution results in decreased site disorder. Importantly, this contrasting site disorder features a significant increase in inter‐slab distance for I and a moderate increase for Br—a critical factor contributing to the sharply contrasting Li^+^ conductivities of the I^−^ and Br^−^‐substituted LZC. Additionally, despite the reduced electrochemical oxidative stability introduced by I, a substantially improved rate capability of LiCoO_2_||Li‐In ASSB cells at 30 °C was demonstrated using Li_2_ZrCl_5.75_I_0.25_ without significantly compromising reversibility. These results highlight the importance of balancing the properties of halide SEs to achieve optimal performance.

## 2 Results and Discussion

### 2.1 Results of Conductivity and Structural Evolution in Li_2_ZrCl_6‐y_X_y_

**Figure 1:**
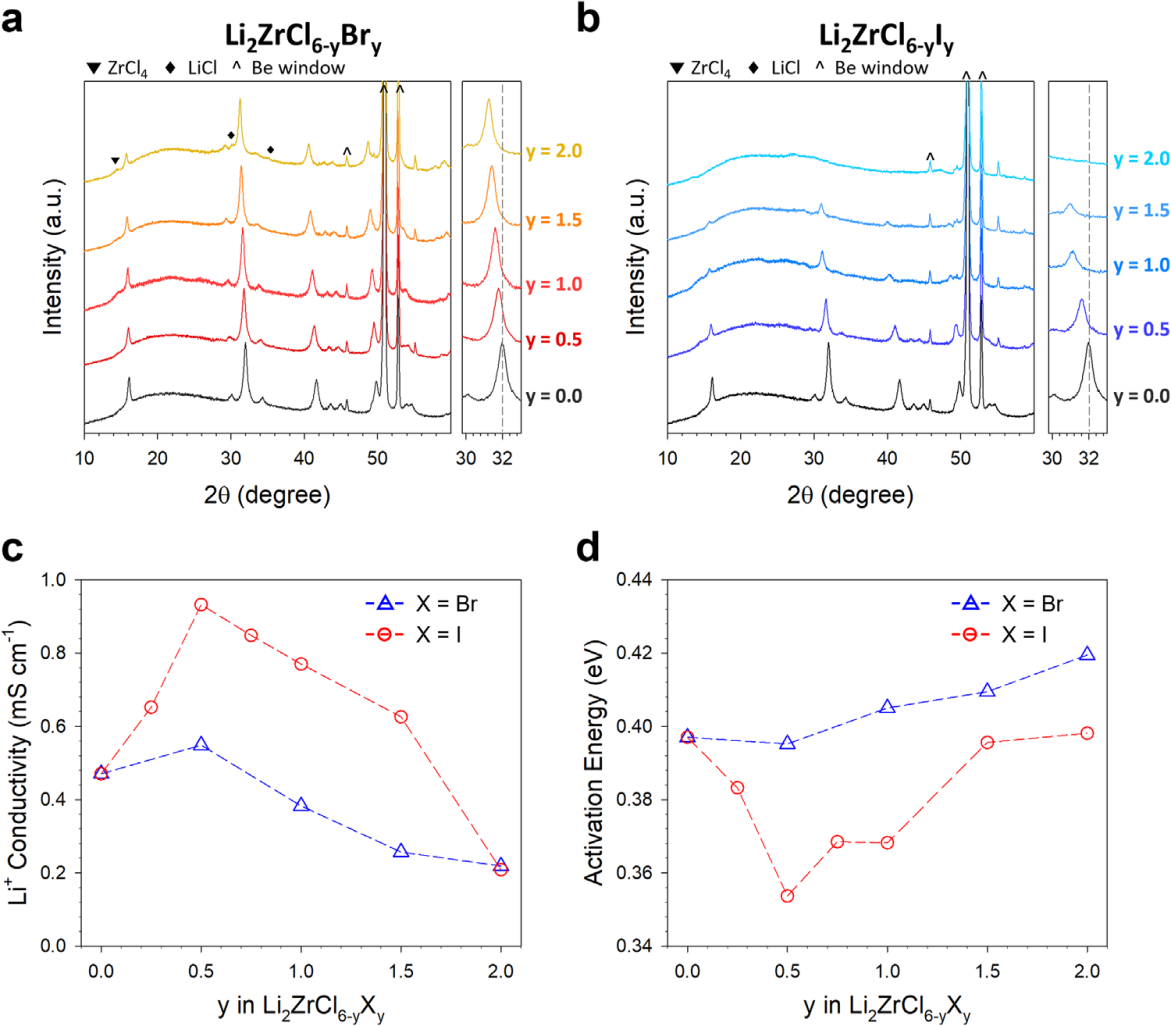
Characterization of Li_2_ZrCl_6‐y_X_y_ (X═Br, I). a,b) XRD patterns of Li_2_ZrCl_6‐y_Br_y_ (a) and Li_2_ZrCl_6‐y_I_y_ (b). c,d) Ionic conductivities at 30 °C (c) and activation energies for Li_2_ZrCl_6‐y_X_y_ (d).

Li_2_ZrCl_6‐y_Br_y_ (LZCB) and Li_2_ZrCl_6‐y_I_y_ (LZCI) were prepared by mechanochemical milling of stoichiometric mixtures of LiCl, LiX (X═Br, I), and ZrCl_4_ for y ≤ 2 and LiCl, LiX, ZrCl_4_, and ZrX_4_ for y > 2. The XRD patterns of LZCB and LZCI are shown in **Figures** **1**a,b, and S1 (Supporting Information). The main reflections of LZC matched those of hcp trigonal Li_3_YCl_6_ (space group *P* $\bar{3}$ *m*1).^[^ ^22^, ^26^ ^]^ As substitution proceeded, most reflections exhibited gradual negative shifts, indicating solid solution behavior, attributed to expanded lattices owing to the larger ionic radii of Br^−^ (1.96 Å) and I^−^ (2.2 Å) than Cl^−^ (1.81 Å).^[^ ^26^, ^30^ ^]^ For LZCB, although impurity peaks emerged at y ≥ 2, the peak shifting continued up to y = 4.0, as shown in Figure S1 (Supporting Information), indicating its solubility limit. The reflection intensities of the LZCB remained constant. However, LZCI exhibits attenuated peak intensities upon substitution, resulting in indistinct peaks at y = 2.0, suggesting the potential formation of an amorphous phase.

The variation in ionic conductivity at 30 °C with activation energy (E_A_) as a function of substitution (y) in a series of Li_2_ZrCl_6‐y_X_y_ (X═Br, I) is shown in Figure 1c,d. Corresponding Arrhenius plots are presented in Figure S2 (Supporting Information). Notably, the I‐substitution led to a rapid increase in Li^+^ conductivity, reaching a maximum of 0.91 mS cm^−1^ in Li_2_ZrCl_5.5_I_0.5_, representing more than a twofold improvement compared to LZC. Subsequent substitution decreased Li^+^ conductivity, recording 0.21 mS cm^−1^ at y = 2.0. Given the significant attenuation of the XRD reflections of I‐substituted LZC, this observation may be associated with the evolution of the amorphous phase (Figure 1b). Conversely, LZCB exhibited a distinctly different trend in Li^+^ conductivity (Figure 1c). Except for y = 0.5, where the Li^+^ conductivity showed a slight improvement, the Li^+^ conductivities remained lower than that of the LZC across all y ranges—thoroughly explored in the section of “Discussions of Conductivity and Structural Evolution in Li_2_ZrCl_6‐y_X_y_”, focusing on the correlation between the local structure and ion conduction mechanism. The E_A_ results are closely correlated with the ionic conductivity: a higher ionic conductivity corresponds to a lower E_A_.

**Figure 2:**
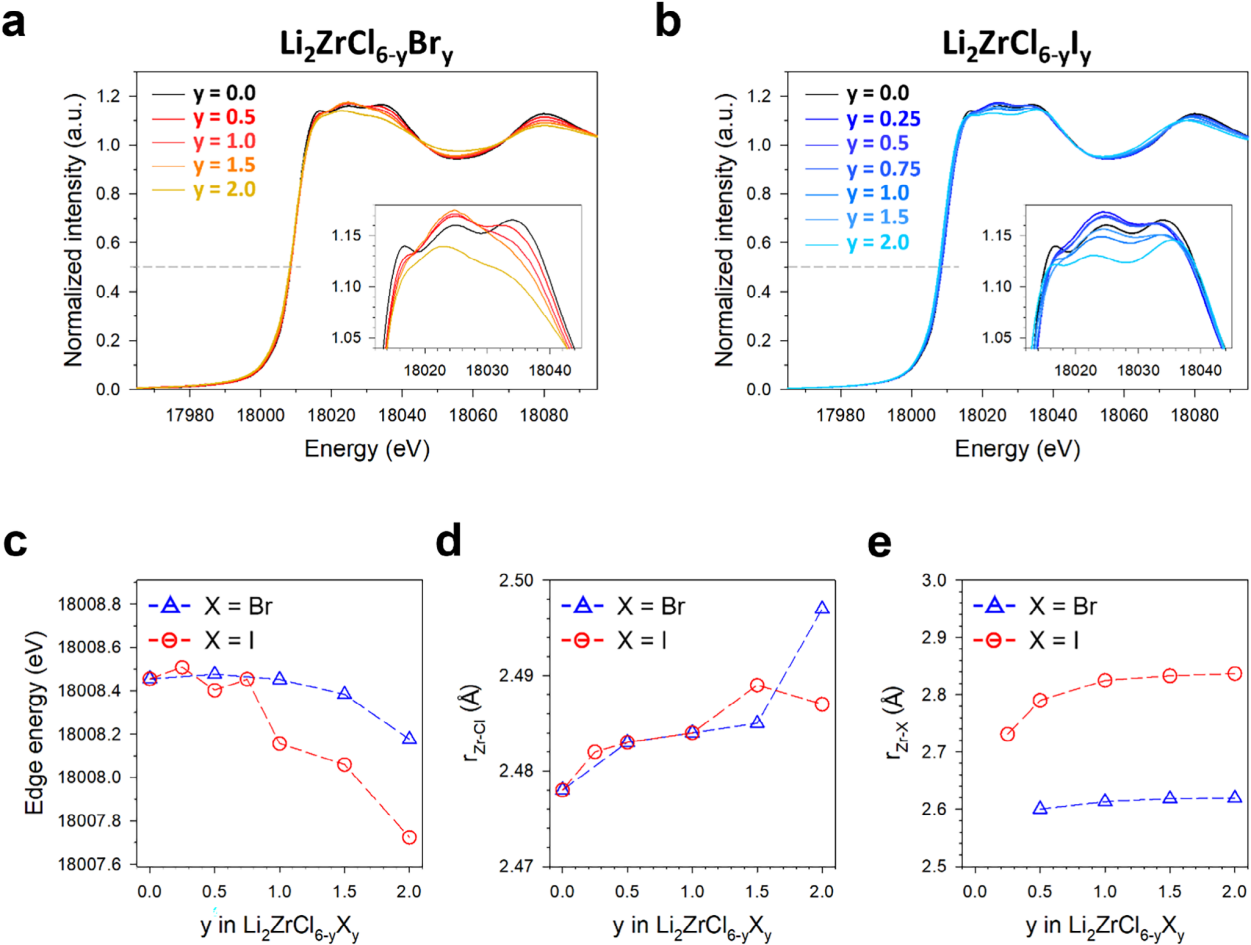
Zr K‐edge XAS results of Li_2_ZrCl_6‐y_X_y_ (X═Br, I). a,b) Zr K‐edge XANES spectra of Li_2_ZrCl_6‐y_Br_y_ (a) and Li_2_ZrCl_6‐y_I_y_ (b). c) Edge energy corresponding to normalized intensity of 0.5 for Li_2_ZrCl_6‐y_X_y_. d,e) Zr─Cl bond length (d) and Zr─X bond lengths (e), as obtained by the EXAFS fitting analysis.

**Figure 3:**
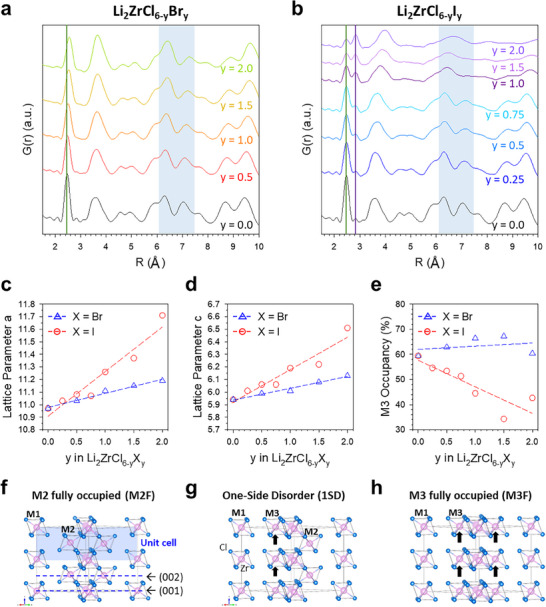
PDF results of Li_2_ZrCl_6‐y_X_y_. a,b) PDF G(r) for Li_2_ZrCl_6‐y_Br_y_ (a) and Li_2_ZrCl_6‐y_I_y_ (b). c,d) Lattice parameters *a* (c) and *c* (d). e) M3 site occupancy of Zr (i.e., M2‐M3 disorder) for Li_2_ZrCl_6‐y_X_y_ (X═Br, I; 0 ≤ y ≤ 2.0). f–h) Crystal structures of Li_2_ZrCl_6_, outlined with the 1 × 1 × 2 supercells for M2 fully occupied (M2F) (f), 1 side disorder (1SD) (g), and M3 fully occupied (M3F) Li_2_ZrCl_6_ (h). Light pink and light blue spheres represent Zr and Cl atoms, respectively.

The local structures of LZCB and LZCI were probed using Zr K‐edge XAS and PDF measurements, as shown in **Figures** **2** and **3**, respectively. These analyses are essential because they provide structural information on both crystalline and amorphous phases.^[^ ^71^, ^72^ ^]^ Figure 2a,b depict the normalized Zr K‐edge X‐ray absorption near‐edge structure (XANES) spectra for LZCB and LZCI, respectively. The main edge position for all samples at ≈18020 eV indicates the tetravalent oxidation state of Zr.^[^ ^26^ ^]^ The edge energy corresponding to a normalized intensity of 0.5, shown in Figure 2c, indicates overall negative shifts with the substitutions. Specifically, the edge energy of LZCB exhibited a gradual decrease, whereas that of LZCI decreased rapidly at y > 0.75. These overall negative shifts are attributed to the substitution of Zr─Cl bonds with Zr─Br or Zr─I bonds.^[^ ^73^ ^]^ The bonding force between Zr and X weakens as the ratio of Zr─Br or Zr─I bonds to Zr─Cl bonds increases,^[^ ^74^, ^75^ ^]^ attributed to the decrease in electronegativity in the order of Cl > Br > I, which correlates with polarizability; Br and I have greater polarizability than Cl.^[^ ^74^, ^76^ ^]^ Additionally, the molar ratio of the Zr─Cl bond to the Zr─Br or Zr─I bond and the bond lengths of each sample were quantitatively determined using Zr K‐edge extended X‐ray absorption fine structure (EXAFS) spectra with first‐shell fitting analysis, as shown in Figure S3 (Supporting Information) along with *k* space data (Figure S4, Supporting Information), and summarized in Figure 2d,e and Table S1 (Supporting Information). The Zr─X bond length increased upon Br and I substitution owing to the larger ionic radii of Br^−^ and I^−^ compared to Cl^−^ (Figure 2d,e). Substituting I significantly increased the average Zr─X bond length from 2.48 Å in LZC to 2.59 Å in Li_2_ZrCl_4_I_2_, larger than the Br substitution case with the increased average Zr─X bond length of 2.54 Å (Table S1, Supporting Information). Furthermore, the best‐fit result of the coordination numbers for the Zr─X bonds confirms that the molar ratio for all samples is in good agreement with the target composition (Table S1, Supporting Information).

PDF refinement was performed to quantify the lattice parameters and M2‐M3 site disorder (Figure 3). More detailed fitting information is provided in the Experimental Section. The PDF fitting results were consistent with those obtained from the EXAFS fitting. The presence of Zr─Cl bonds in LZCB and LZCI is evidenced by a peak ranging from 2.2 to 2.7 Å, with a maximum at ≈2.47 Å, as depicted by the light green line in Figure 3a,b. This peak shifts positively with increasing Br substitution, reaching a maximum at ≈2.53 Å. In contrast, with I substitution, this peak does not shift, but instead, another peak at ≈2.84 Å gradually increases in intensity relative to the Zr─Cl peak (purple line in Figure 3b), indicating an increase in the proportion of longer Zr─I bonds. Additionally, the changes in the lattice parameters are shown in Figure 3c,d. In contrast to the gradual lattice expansion in both *a* (=*b*) and *c* for LZCB, LZCI exhibits a more pronounced increase in lattice parameters.

The M2‐M3 disorder can be defined as a phenomenon in which Zr atoms shift from the (002) plane (i.e., M2 sites) to the (001) plane (i.e., M3 sites). Such M2‐M3 site disorder is known to alter the repulsive force between Li and Zr, thereby influencing the ionic conductivity.^[^ ^24^, ^65^, ^67^ ^]^ The intensity changes of PDF peaks at 6.5 and 7.1 Å, corresponding to the bond distances between M1‐M3 and M1‐M2, respectively, directly confirming the degree of M2‐M3 site disordering (highlighted in blue in Figure 3a,b; Figure S5, Supporting Information).^[^ ^24^, ^26^ ^]^ The occupancy of Zr at the M3 sites in Li_2_ZrCl_6‐y_X_y_ (X═Br, I) (LZCX) obtained from PDF fitting is shown in Figure 3e. Detailed refinement results are presented in Figures S6–S10 and Table S2 (Supporting Information). Unexpectedly, the M3 site occupancy in LZCI gradually decreased from 59.4% for LZC to 42.0% for Li_2_ZrCl_4_I_2_ with enhanced Li^+^ conductivity. In contrast, the M2‐M3 disorder increased up to 74.0% in Li_2_ZrCl_4_Br_2_, which exhibited lower ionic conductivity. The findings in Br^−^ and I^−^ substituted LZC contradict the trend observed in other halide SEs, such as Li_3_YCl_6_ and Li_3_ErCl_6_, where increased ionic conductivities are associated with increased M2‐M3 disorder.^[^ ^24^, ^65^, ^67^ ^]^ Additionally, the distinct preference for M2‐M3 disorder between Br^−^ and I^−^ substituted LZCX was confirmed by evaluating the energy above hull (E_hull_) as a measure of structural stability, with the results summarized in Table S3 (Supporting Information). The underlying origin of this preference can likely be explained by the extent of lattice expansion, which influences the accommodation of metal ions in the intermediate layer (M2 site), depending on the size of the substituted anions and the resulting structural adjustments. Further details are provided in Note S1 (Supporting Information), accompanied by Table S4 (Supporting Information). The effects of M2‐M3 disorder on ionic conductivity will be thoroughly investigated in the following section based on three model structures with varying degrees of M2‐M3 disorder, as depicted in Figure 3f–h: 1) “M2 fully occupied (M2F)‐LZC” with the original hcp structure of Li_2_ZrCl_6_, 2) “one‐side disordered (1SD)‐LZC” in which one of two ZrCl_6_ octahedra in the (002) plane (M2) ascends to the (001) plane (M3), 3) “M3 fully occupied (M3F)‐LZC” in which all ZrCl_6_^2−^ octahedra in the (002) plane are lifted to the (001) plane.

To provide further clarification, we performed high‐resolution powder diffraction (HRPD) measurements on Li_2_ZrCl_6‐y_X_y_ (X═Br, I, 0 ≤ y ≤ 2.0) and analyzed the data using the Rietveld refinements, except for Li_2_ZrCl_4_I_2_, where the refinement was inapplicable due to its highly distorted structure (Figures S11 and S12; Tables S5–S15, Supporting Information). The refinement results corroborated the XRD data, confirming the reliability of our structural analysis. Additionally, both *a* and *c* lattice parameters exhibited elongation with increasing *y* values for both X═Br and I, indicating successful substitution at the bulk level (Table S16, Supporting Information). Furthermore, the trends in M2 and M3 site occupancy were consistent with the PDF fitting results, reinforcing the validity of our quantitative structural analysis and relativity of the refinement results (Table S16, Supporting Information).

The ionic conductivities and structural characterizations of LZC, LZCB, and LZCI demonstrated that the Br^−^ and I^−^ substitutions in LZC resulted in distinct structural and Li^+^ conductivity trends. Despite negative XRD peak shifts in both LZCB and LZCI owing to the larger ionic sizes of Br^−^ and I^−^ than Cl^−^, notable peak broadening was observed only in LZCI, particularly at y = 2.0. The Li^+^ conductivity significantly increased up to y = 0.5 and subsequently decreased for LZCI, whereas lower ionic conductivities were observed for most compositions of LZCB. Significantly, unusual behaviors associated with M2‐M3 disorder were observed: contrasting trends in the M2‐M3 disorder, i.e., increasing M2‐M3 disorder in LZCB and decreasing M2‐M3 disorder in LZCI with substitution. These observations highlight the complex interplay between structural distortions including potential amorphization, anion substitution, structural evolution, and ionic conductivity in these materials—thoroughly discussed in the following section.

### 2.2 Discussions of Ionic Conductivity and Structural Evolution in Li_2_ZrCl_6‐y_X_y_

The key factors influencing the variation in the Li^+^ conduction mechanism include structural distortions, lattice softness owing to anion polarizability, lattice parameters or inter‐slab distances, and M2‐M3 disorder. In this section, we further investigate these effects.

#### 2.2.1 Structural Distortions in Li_2_ZrCl_6‐y_X_y_

We performed melt‐quenching AIMD calculations to predict potential amorphous structures (Figures S13–S17, Supporting Information), with the procedure illustrated in Figure S18 (Supporting Information). The predicted structures revealed a peak ≈4 and 5 Å, corresponding to Zr─Zr bonds, distinct from the discrete peaks at 6 Å characteristic of crystalline Li_2_ZrCl_6_ (M2F, 1SD, M3F). However, Zr K‐edge EXAFS and PDF analyses revealed no such short Zr─Zr bonds (Figure 3a,b; Figure S3, and Tables S1 and S2, Supporting Information), which indicates the absence of distinct amorphous phases. Instead, I substitution induces distortions in the local structure, resulting in peak broadening in the PDF.^[^ ^77^ ^]^

To further clarify the absence of distinct amorphous phases, we performed additional PDF analyses. We constructed a relaxed *P* $\bar{3}$ *m*1 fitting model for LZCI and LZCB based on a trigonal crystalline structure, assuming uniform substitution across all 6i anion sites (e.g., Cl, Br, and I) in the LZCX lattice. To account for local distortions, we freely relaxed two out of three 6i anion sites from the *P* $\bar{3}$ *m*1 symmetry for LZCB and LZCI having higher Br and I compositions. For LZCI with *y* ≤ 0.75, satisfactory fitting results within the range of 1.5 ≤ r ≤ 20 Å were achieved without residual peaks, yielding R_W_ values of up to 14% (Figure S8 and Table S2, Supporting Information), confirming the presence of a single crystalline phase. However, for *y* > 0.75, the R_W_ value sharply increased to 35.6% in Li_2_ZrCl_4_I_2_ using a relaxed trigonal crystalline model structure (Figure S9 and Table S2, Supporting Information). To address this discrepancy, the fitting process was divided into short (1.5 ≤ r ≤ 10 Å) and long (10 ≤ r ≤ 20 Å) ranges (Figure S10 and Table S17, Supporting Information). For y = 1.0 and 1.5 in Li_2_ZrCl_6‐y_I_y_, both fittings yielded low R_W_ values (6.3–12.8%), indicating the coexistence of distorted local structures. These distortions, caused by significantly longer Zr─I bonds than Zr─Cl bonds, lead to polyhedral distortions distributed in multiple directions within the lattice. Consequently, a single model over a wide fitting range fails to represent the structure accurately. Notably, when the fitting ranges were divided, both short‐ and long‐r range structures were well described by a relaxed *P* $\bar{3}$ *m*1 model structure. Collectively, these findings confirm the absence of new amorphous phases in our samples.

Amorphous materials exhibit only short‐range (or local) order, typically spanning a few angstroms, with no long‐range structural coherence.^[^ ^78^ ^]^ The successful fitting of the 10–20 Å range indicates that distorted yet ordered trigonal phases persist at larger length scales. While a single crystalline model cannot fully capture the overall structure due to the coexistence of highly distorted structures, these distortions originate from the parent structure rather than from forming a new amorphous phase. Consequently, assuming that changes within the crystalline phase—specifically within the unit cell—are the primary factors influencing ionic conductivity, we will discuss other contributing factors in the following section in detail.

#### 2.2.2 Li^+^ Conduction in Disordered Li_2_ZrCl_6_

Previous reports have underscored the significance of the cation arrangement within trigonal halide SEs.^[^ ^65^, ^67^ ^]^ Specifically, the M2‐M3 disorder in trigonal Li_3_MCl_6_ (M═Y, Er) leads to a reordering of the Li^+^ distribution, attributed to Coulombic repulsion effects. This reordering induces changes in the bottleneck sizes along the diffusion pathways, particularly in the *c*‐direction.^[^ ^24^ ^]^ However, as described in Section “2.1 Results of Conductivity and Structural Evolution in Li_2_ZrCl_6‐y_X_y_”, the M2‐M3 disorder in trigonal LZC showed different trends in Li^+^ conductivity (Figure 1c): increased conductivity upon I‐substitution along with decreased M2‐M3 disorder and marginal change or decreased conductivity upon Br substitution along with increased M2‐M3 disorder. The mechanochemical synthesis of trigonal LZC introduces an M2‐M3 disorder, thereby establishing a favorable conduction pathway in the *c*‐direction.^[^ ^24^ ^]^ However, this M2‐M3 disordering can impede Li^+^ diffusion along the intra‐plane direction in trigonal LZC.

**Figure 4:**
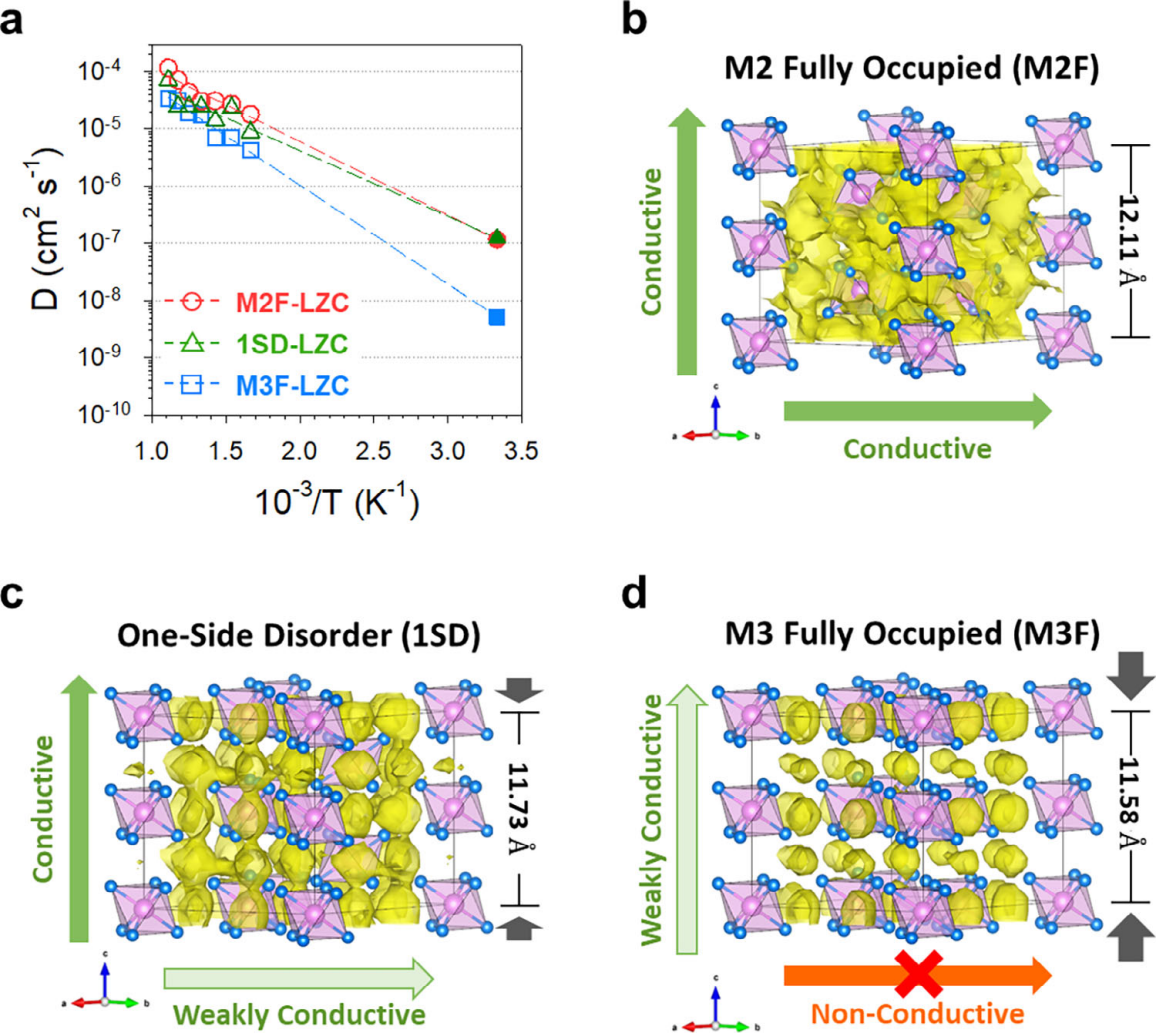
AIMD simulation results of LZC. a) Arrhenius plots of AIMD simulation of M2F‐Li_2_ZrCl_6_, 1SD‐Li_2_ZrCl_6_, and M3F‐Li_2_ZrCl_6_ and extrapolated diffusivity of each structure in 300K. b–d) Probability density (P = P_max_/100) of AIMD simulation in 600K during 300 ps of M2F‐LZC (b), 1SD‐LZC (c), and M3F‐LZC (d). Light pink and light blue spheres represent Zr and Cl atoms, respectively, while yellow surfaces denote Li probability densities in (b,c).

**Figure 5:**
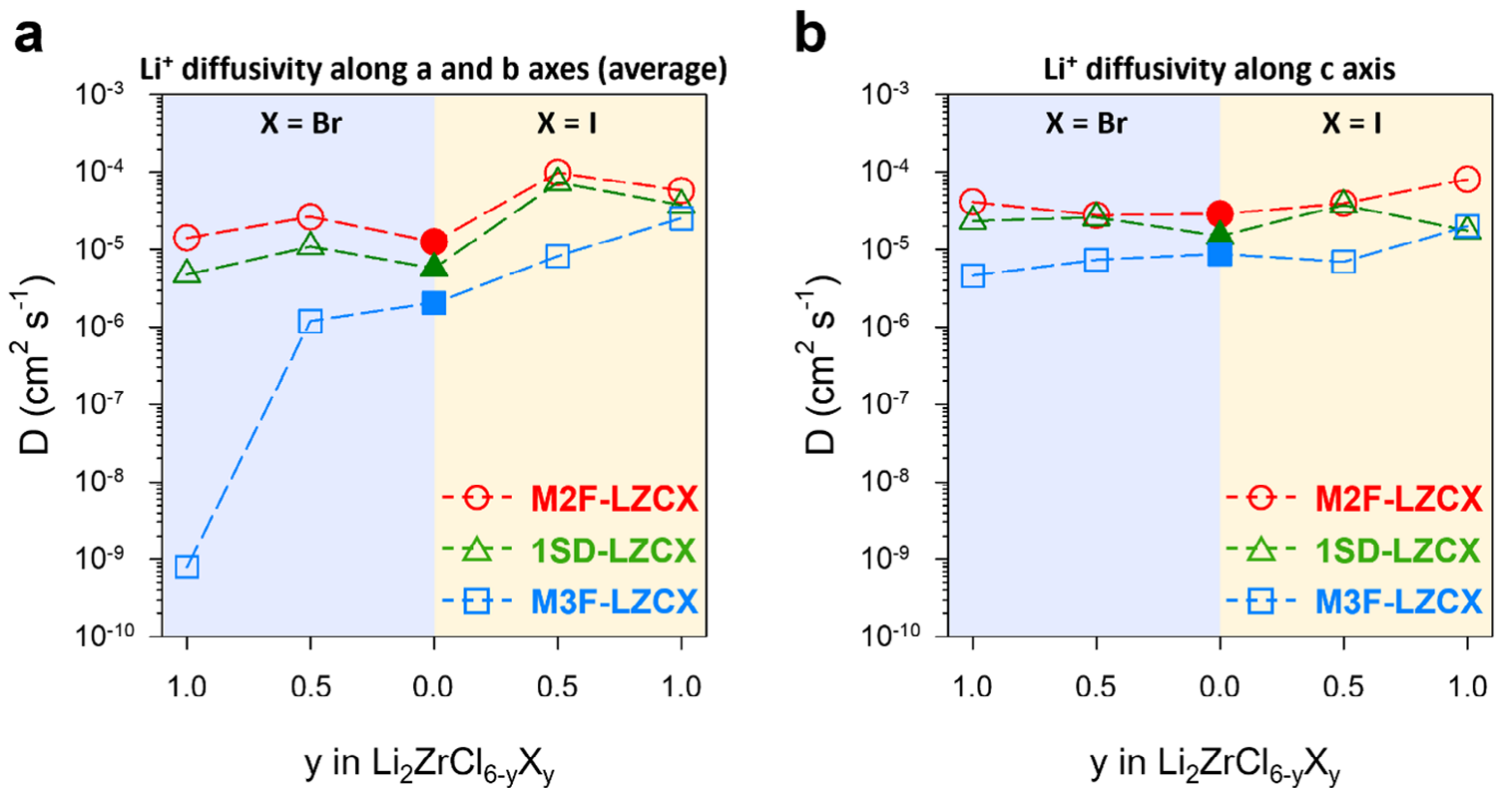
Li^+^ diffusivity at 600 K along *a*, *b*, and *c* axes, calculated by AIMD simulation. a) Root mean square value of Li^+^ diffusivity in the *a* and *b* axes directions. b) Li^+^ diffusivity in the *c*‐axis direction.

To investigate this possibility, we performed AIMD simulations based on three model configurations of the LZC, as shown in Figure 3f–h. The findings revealed that the M3F‐LZC structure exhibited lower diffusivity than M2F‐LZC and 1SD‐LZC (**Figure** **4**a). To explore the underlying effect, we plotted the mean square distance (MSD) of the 600 and 700 K AIMD simulations for three structures: M2F‐LZC, 1SD‐LZC, and M3F‐LZC, as shown in Figure S19 (Supporting Information). Notably, in M3F‐LZC, conduction along the ab intra‐plane was more restricted than that in M2F‐LZC and 1SD‐LZC. To ascertain the impact of each conduction path on Li^+^ diffusion, we depict the isosurfaces of the Li^+^ probability density at 600 K in Figure 4b–d. The Li^+^ probability densities of M2F‐LZC and 1SD‐LZC exhibited percolated Li^+^‐conducting pathways, whereas in M3F‐LZC, these pathways remained disconnected and isolated. In the (001) plane, the Li^+^ conduction pathway does not percolate owing to its complete occupation by the ZrCl_6_^2−^ octahedra, leading to heightened Coulombic repulsion forces within the intra‐plane. The repulsion between Zr^4+^ and Li^+^ increased the activation barrier for intra‐plane Li^+^ conduction. Notably, in the M3F structure, the c‐lattice parameter was reduced (Figure S20 and Table S18, Supporting Information), reflecting an insufficient volume for effective intra‐plane Li^+^ conduction.^[^ ^79^ ^]^ The inter‐slab distance in M3F‐LZC is restricted because the ZrCl_6_ octahedra and Li^+^, which serve as pillars to maintain the inter‐slab distance, are absent in the (002) plane, reducing the distance between the ZrCl_6_ octahedra along the c‐axis direction and consequently impeding intra‐plane conduction (Figure 4b–d; Figure S20, Supporting Information). This phenomenon—impeded ab plane conduction owing to the reduced inter‐slab distance—is more pronounced in LZC than in Li_3_MCl_6_ (M═Y, Er) owing to the smaller ionic radii of Zr^4+^ (0.72 Å) compared to Y^3+^ (0.90 Å), the higher valence state of Zr than that of Y, and the lower concentration of Li in Li_2_ZrCl_6_ compared to Li_3_YCl_6_ (Tables S18 and S19, Supporting Information).^[^ ^68^ ^]^ The Li^+^ diffusivity differences in each direction based on the occurrence of M2‐M3 disorder are presented in Table S20 (Supporting Information), with the results plotted in **Figure** **5**. Comparing the three LZC structures, the diffusivity decreased in the order of M2F > 1SD > M3F. Notably, a significant reduction in the diffusivity was observed along the a‐ and b‐axes directions, with this reduction being ≈1 order of magnitude.

#### 2.2.3 Regulating the Ionic Conductivity of Li_2_ZrCl_6_ by Anion Substitution

**Figure 6:**
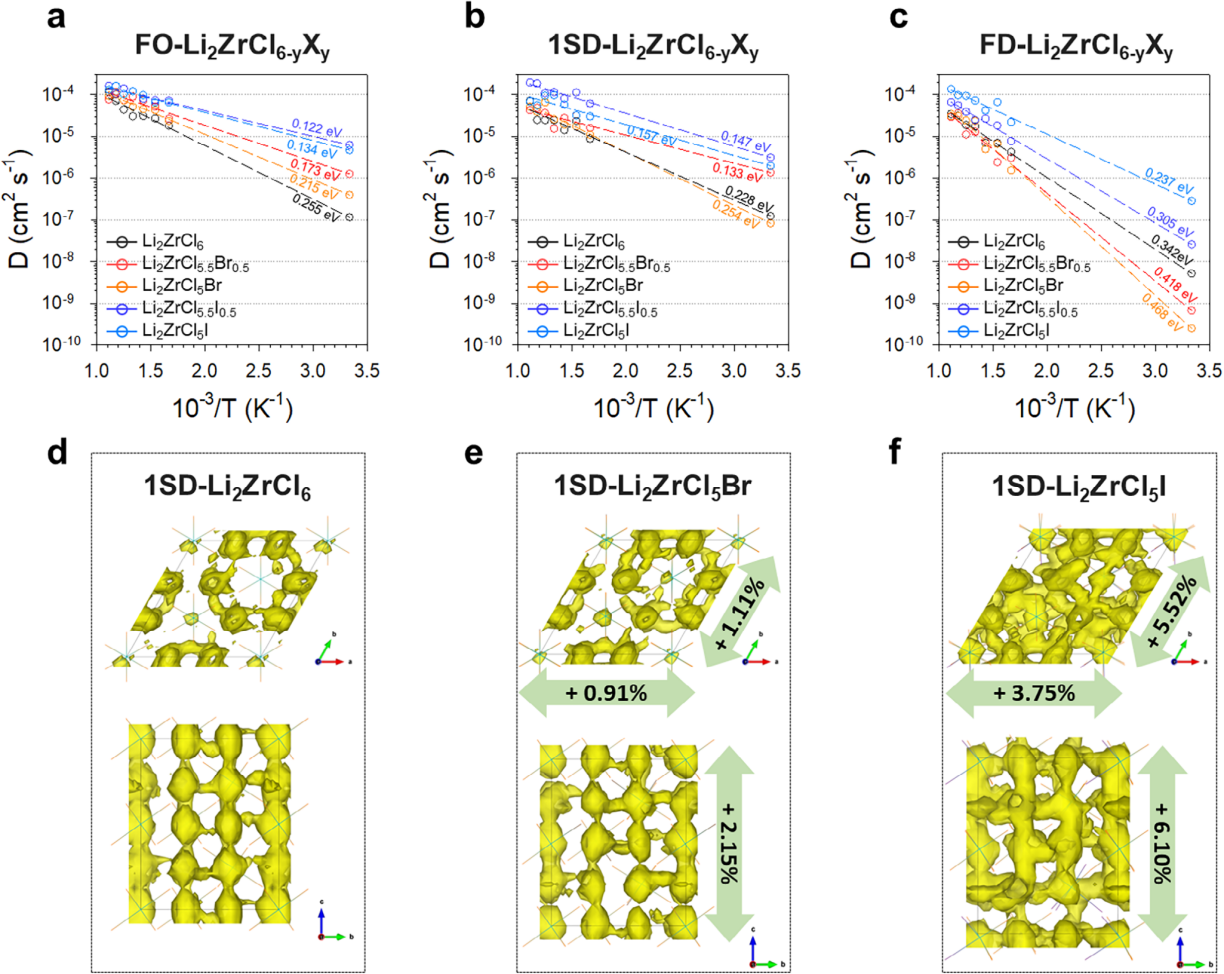
AIMD simulation results of anion‐substituted LZCX. a–c) Arrhenius plots of ionic conductivity for AIMD simulation with extrapolated diffusivity at 300 K for M2F‐LZCX (a), 1SD‐LZCX (b), and M3F‐LZCX (c). The activation energy is also provided in units of eV. d–f) The composition of anion‐substituted structures LZCX is Li_2_ZrCl_5.5_Br_0.5,_ Li_2_ZrCl_5_Br, Li_2_ZrCl_5.5_I_0.5_, and Li_2_ZrCl_5_I. Li Probability density at 600 K during 300 ps (isosurface value P = P_max_/100), as obtained by AIMD simulation of 1SD structured Li_2_ZrCl_6_ (d), Li_2_ZrCl_5_Br (e), and Li_2_ZrCl_5_I (f). The inserted percentage is the lattice parameter elongation ratio in each axis direction. Zr─Cl, Zr─Br, and Zr─I bonds are represented by thin lines, while yellow surfaces denote Li probability densities in (d–f).

To uncover the effects of M2‐M3 disorder on Li^+^ conduction in anion‐substituted LZC, we conducted AIMD simulations for anion‐substituted M2F, M3F, and 1SD structures with the following compositions: Li_2_ZrCl_5.5_Br_0.5,_ Li_2_ZrCl_5_Br, Li_2_ZrCl_5.5_I_0.5_, and Li_2_ZrCl_5_I. The resulting Arrhenius plots of the ionic conductivities are shown in **Figure** **6**a–c. In M2F‐type structures, anion‐substituted LZC exhibited increased diffusivities compared to those of the LZC (Figure 6a), attributed to the enlarged lattice volume, which creates expanded Li^+^ transport channels (Figure S21 and Table S4, Supporting Information).^[^ ^30^ ^]^ The MSD and Li^+^ probability density of the M2F‐type structures (Figure S22, Supporting Information) reveal that the Li^+^ conduction pathways are well‐percolated in 3D across all M2F‐type structures. Additionally, the enhanced softness of the lattice owing to the introduction of more polarizable anions could be another contributing factor to the enhanced diffusivity.^[^ ^34^, ^35^, ^36^ ^]^

However, the 1SD and M3F structures displayed distinct tendencies compared to the M2F structures (Figure 6b–f; Figures S23 and S24, Supporting Information). As depicted in Figure 6b, while 1SD‐Li_2_ZrCl_5.5_Br_0.5_ exhibits increased diffusivity compared to 1SD‐LZC, the diffusivity of 1SD‐Li_2_ZrCl_5_Br decreases despite the expansion of the lattice volume and an increase in the lattice parameter *c* (Table S4, Supporting Information). This tendency is even more pronounced in the M3F structures (Figure 6c; Table S4, Supporting Information), where both M3F‐Li_2_ZrCl_5.5_Br_0.5_ and M3F‐Li_2_ZrCl_5_Br exhibited lower diffusivity compared to M3F‐LZC. In stark contrast, both 1SD and M3F structures of I‐substituted LZC (Li_2_ZrCl_5.5_I_0.5_ and Li_2_ZrCl_5_I) showed higher diffusivity than LZC (Figure 6b,c), consistent with the experimental results (Figure 1). The MSD plots of the 600 K AIMD simulations for all 1SD and M3F LZCX confirmed the aforementioned trends (Figures S23 and S24, Supporting Information).

1SD‐Li_2_ZrCl_5.5_I_0.5_ and 1SD‐Li_2_ZrCl_5_I exhibited lattice volume expansions of ≈14–20% (Table S4, Supporting Information), resulting in larger channel sizes compared to the 1SD‐LZC structure and active diffusion within the intra‐planes. In contrast, 1SD‐Li_2_ZrCl_5_Br shows only a 3.6% expansion in the lattice volume, particularly insufficient in the *c* direction. Consequently, the conduction path and channel size did not significantly change (Figures S21–S23, Supporting Information). In the M3F structures, this change in diffusivity and the difference in the degree of expansion between LZCB and LZCI were more noticeable (Figure 6c; Figure S25, Supporting Information). Both M3F‐Li_2_ZrCl_5.5_Br_0.5_ and M3F‐Li_2_ZrCl_5_Br exhibited lower diffusivities than M3F‐LZC. Conversely, the I‐substituted M3F structures showed reinforced diffusion compared to M3F‐LZC (Figure 6c). The reduced diffusivity of the Br‐substituted M3F‐LZC is attributed to its failure to induce significant expansion along the *c*‐axis, which in turn impedes Li^+^ conduction within the *ab*‐plane. This indicates that large anions (Br^−^) in the narrow conduction path hinder conduction more than the increased polarizability would facilitate it. In contrast, I^−^ substitution leads to sufficient expansion of the conduction channel and enhanced lattice polarizability, both of which contribute to improved Li^+^ conductivity.

To further elucidate the role of lattice polarization on ionic conductivity, we performed electrostatic potential and electron localization function (ELF) analyses, which revealed that heavier halide substitution (Br^−^, I^−^) induces electron delocalization, structural asymmetry, and local potential flattening – contributing to lower Li^+^ migration barriers. These effects were more pronounced for I^−^ than Br^−^. Further detailed discussion can be found in Notes S2 and S3 (Supporting Information), accompanied by Figures S19–S24 (Supporting Information), S33–S36, and Tables S4–S21 (Supporting Information). This hindrance effect is reflected in the decreased Li^+^ conductivity or diffusivity observed in Br^−^ substituted compositions in both experimental and theoretical calculations. Furthermore, substitution with Br^−^ induces M2‐M3 disorder, i.e., an increase in M3 site occupancy (Figure 3e), exacerbating the intra‐plane blocking effect. However, the much larger I^−^ anions lead to substantial expansion along the *c*‐axis in disordered structures, facilitating overall Li^+^ conduction and opening the *ab*‐plane conduction path without the hindrance effect of larger anions. Interestingly, even in the presence of M2‐M3 disorder, the diffusivities in the *a*‐ and *b*‐axes directions of the 1SD and M3F structures of LZCI were comparable to or exceeded those of M2F‐LZC (Figure 5; Tables S4–S20, Supporting Information). This result is because the increase in the inter‐slab distance of LZCI induced by I substitution is sufficiently large to effectively offset the decrease in the inter‐slab distance caused by the M2‐M3 disorder, unlike the case of LZCB.

This is also supported by the activation energy values obtained from both experiments and first‐principles simulations (Figure S27, Supporting Information). Br substitution intrinsically increases the occupancy at the M3 site, thereby promoting the formation of M3F configurations. This structural evolution leads to a reduction in inter‐slab distance and consequently results in higher activation energies, as observed in both experimental measurements and first‐principles simulations, which are detrimental to Li^+^ conduction. In contrast, I substitution favors increased occupancy at the M2 site, thereby stabilizing configurations closer to 1SD or M2F. Even in cases where M3F configurations are present, the significant lattice expansion induced by I incorporation mitigates the transport barrier, resulting in lower activation energies and improved ionic conductivity.

The anionic hindrance effects also explain the occurrence of optimal Li^+^ conductivity at a specific substitution level (y = 0.5) in both Br^−^ and I^−^ substituted LZC (Li_2_ZrCl_6‐y_X_y_) (Figure 1c). Further details are provided in Note S4 (Supporting Information), along with Figures S19–S26, and Tables S3–S18 (Supporting Information).

The discussions thus far offer insights into the complex interplay between structural factors (lattice size and M2‐M3 disorder) and Li^+^ conduction behavior in anion‐substituted LZC compounds. The following structural‐transport correlations can be inferred:

- Impeded intra‐plane diffusion in Li_2_ZrCl_6_: This phenomenon is attributed to the heightened Coulombic repulsion forces between Zr^4+^ and Li^+^ within the (001) plane, coupled with a reduction in the inter‐slab distance in the (002) plane, caused by M2‐M3 site disorder. The smaller radius and tetravalency of Zr^4+^, along with the lower Li^+^ concentration in the LZC, exacerbate these effects, leading to less effective intra‐plane conduction compared to Li_3_MCl_6_ (where M = Y^3+^ or Er^3+^ with larger ionic radii).
- Increased conductivity in anion‐substituted M2F structures: This phenomenon is attributed to the enlarged lattice volume, which results in larger Li+ transport channels, particularly in the ab‐plane, thereby improving Li+ conductivity.
- Various tendencies in anion‐substituted LZC with M2‐M3 site disordered structures: Despite lattice expansion, disordered Li_2_ZrCl_5_Br exhibited decreased diffusivity, whereas increased diffusivity was observed in I‐substituted LZC. The underlying mechanism is the sufficiently enlarged channel size in the I‐substituted LZC, which is not significantly affected by the M2‐M3 disorder. The enlarged channel size facilitates active diffusion within the intra‐plane. In contrast, Br substitution in the LZC did not lead to a sufficiently enlarged intra‐plane conduction channel. Additionally, the increased M2‐M3 disorder caused by Br substitution reduces the channel size, further impeding Li^+^ conduction.

Anion substitution in LZC can effectively promote Li^+^ diffusion by increasing the size and polarizability of the lattice structures. However, when anion substitution fails to sufficiently expand the lattice volume or induces unfavorable changes in the conduction path, it can narrow the diffusion channels for Li^+^, thereby impeding conductivity.

### 2.3 Electrochemical Performance of Li_2_ZrCl_6‐y_I_y_

**Figure 7:**
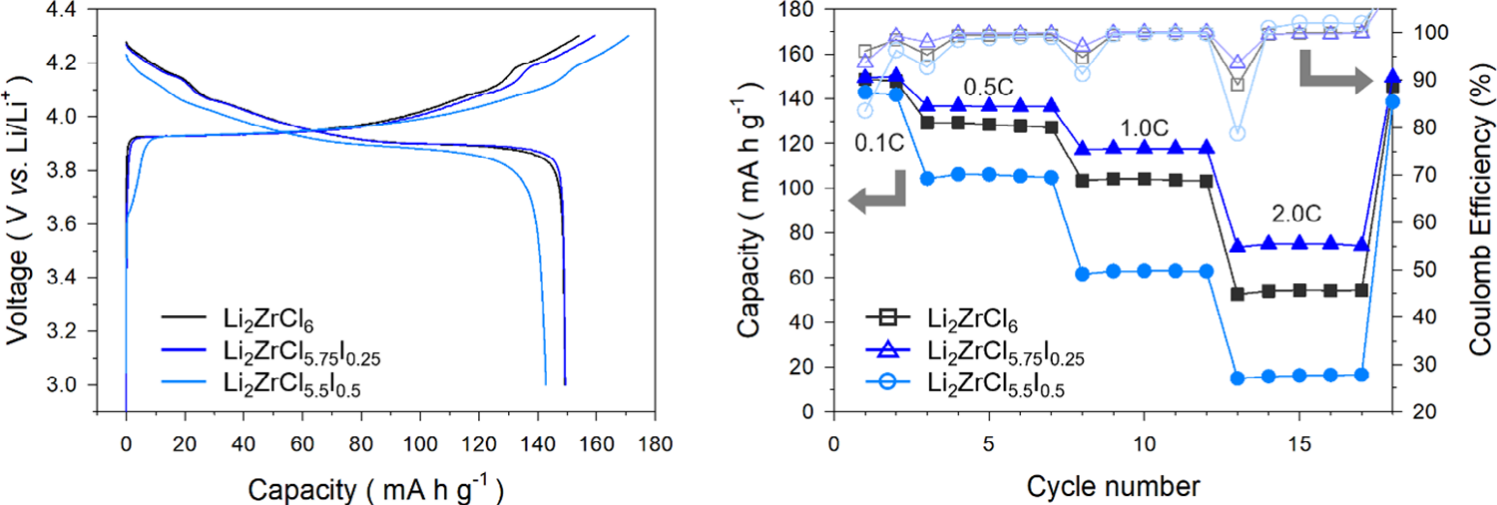
Electrochemical performance of LiCoO_2_||Li‐In cells at 30 °C, employing LZC and I‐substituted LZC as catholyte. a,b) First‐cycle charge–discharge voltage profiles at 0.1C (16.1 mA g^−1^) (a) and corresponding rate capabilities with Coulombic efficiencies (b).

To The applicability of the mechanochemically prepared Li_2_ZrCl_6_, Li_2_ZrCl_5.5_I_0.5_, and Li_2_ZrCl_5.75_I_0.25_ for ASSBs was assessed by implementing them as catholytes in LiCoO_2_|Li_6_PS_5_Cl|Li‐In cells at 30 °C (**Figure** **7**) using uncoated LiCoO_2_. Figure 7a shows the first‐cycle charge–discharge voltage profiles at 0.1C (16.1 mA g^−1^) for the LiCoO_2_ electrodes with three different halide SEs. Cells with Li_2_ZrCl_5.5_I_0.5_ showed distinctly different features at the beginning of the first charge compared with others: a sloping voltage profile starting at ≈3.5 V (vs Li/Li^+^), indicating poor electrochemical oxidation stability. Moreover, substantial polarization was observed in the subsequent discharge voltage profile, resulting in a low discharge capacity of 134 mA h g^−1^ and poor initial Coulombic efficiency (ICE) of 79.5%. These values were lower compared to using Li_2_ZrCl_6_ (discharge capacity of 155 mA h g^−1^ and ICE of 97.7%)—an expected outcome given the reduced electrochemical oxidative stability of iodide compounds compared to that of chlorides.^[^ ^19^ ^]^ The same trend was observed for rate capability (Figure 7b). In contrast, LiCoO_2_ electrodes employing a composition with a lower amount of I, Li_2_ZrCl_5.75_I_0.25_, exhibited a much higher initial discharge capacity and ICE of 155 mA h g^−1^ and 93.0%, respectively. This behavior is consistent with cyclic voltammetry (CV) results (Figure S28, Supporting Information), where Li_2_ZrCl_5.5_I_0.5_ exhibits an onset potential near 3.5 V (vs Li/Li^+^), corroborating the sloping charge profile of early oxidative decomposition (Figure 7a). In contrast, Li_2_ZrCl_5.75_I_0.25_ displays a significantly lower oxidative current density with a gradual rise, resembling the response of Li_2_ZrCl_6_, albeit with slightly higher current levels.

The superior performance of cells with Li_2_ZrCl_5.75_I_0.25_ over those with Li_2_ZrCl_5.50_I_0.50_ is noteworthy, given the lower Li^+^ conductivity (0.65 mS cm^−1^) compared to that of Li_2_ZrCl_5.5_I_0.5_ (0.91 mS cm^−1^), thus underscoring the importance of the electrochemical stability. Despite a lower ICE for Li_2_ZrCl_5.75_I_0.25_ (93.0%) compared to Li_2_ZrCl_6_ (97.7%), the discharge capacity value at 0.1C is comparable to that using Li_2_ZrCl_6_. Notably, cells employing Li_2_ZrCl_5.75_I_0.25_ exhibited a superior rate capability compared to those using Li_2_ZrCl_6_, particularly at the high rate of 2C (Figure 7b). These electrochemical performance results highlight the significance of balancing ionic conductivity and electrochemical stability. Ex situ X‐ray photo electron spectroscopy (XPS) analysis revealed that I substitution in Li_2_ZrCl_6_ induces more pronounced side reactions (Figure S29, Supporting Information), which accounts for the slightly inferior cycling performance of cells using Li_2_ZrCl_5.75_I_0.25_ catholyte compared to those with Li_2_ZrCl_6_ (Figure S30, Supporting Information). In both compositions, ZrO_2_ was identified as a major degradation product. Notably, ZrI_4_ evolution was additionally observed when LZCI was used as the catholyte, suggesting more severe oxidative decomposition. The presence of ZrO_2_ may arise from interfacial reactions involving lattice oxygen released from LiCoO_2_.^[^ ^80^, ^81^ ^]^ A comparison of reported halide SEs in terms of cell performance, ionic conductivity, and estimated precursor cost is provided in Table S22 (Supporting Information).

## 3 Conclusion

In summary, we systematically investigated the structural effects on the ion conduction mechanisms in a series of anion‐substituted Li_2_ZrCl_6_ and Li_2_ZrCl_6‐y_X_y_ (X═Br, I). Combined local structure analysis based on synchrotron X‐rays and AIMD calculations revealed that increased M2‐M3 disorder (i.e., increased M3 site occupancy) leads to a contraction in the inter‐slab distance, attributed to the reduced amounts of Zr (at the M2 site) in the slab, which acts as a structural pillar. The constricted inter‐slab distance impedes intra‐plane Li^+^ conduction, a mechanism particularly pronounced in the LZC owing to the heightened Coulombic repulsion forces in the (001) plane, coupled with a reduction in the inter‐slab distances in the (002) plane. This effect originates from the smaller size and tetravalency of Zr^4+^, along with the lower Li^+^ concentration, compared with other disordered Li_3_MCl_6_ (M═Y^3+^ or Er^3+^ with larger ionic radii). While Br and I substitution in LZC can enlarge the lattice volume, each substitution resulted in contrasting changes in Li^+^ conductivity: increased Li^+^ conductivity for LZCI from 0.40 to 0.91 mS cm^−1^ (y = 0.50) but comparable or decreased Li^+^ conductivity for LZCB. The underlying mechanism—I‐substitution led to sufficient lattice expansion and significantly increased the inter‐slab spacing—was revealed. This expansion also benefits from reduced M2‐M3 disorder. In contrast, Br substitution causes insufficient lattice expansion, aggravated by increased M2‐M3 disorder. Additionally, the best rate capability of LiCoO_2_||Li‐In ASSB cells was demonstrated by balancing the ionic conductivity and electrochemical stability by selecting an optimal substitution level (Li_2_ZrCl_5.75_I_0.25_). These results provide valuable insights into the design of new halide SEs in terms of their structural and chemical complexities.

## 4 Experimental Section

### Preparation of Materials

Li_2_ZrCl_6‐y_Br_y_ (LZCB) and Li_2_ZrCl_6‐y_I_y_ (LZCI) were prepared through ball‐milling of LiCl, LiX (X═Br, I), and ZrCl_4_ for y ≤ 2 and LiCl, LiX (X═Br, I), ZrX for y > 2. The stoichiometric mixture of ZrCl_4_ (99.99%, Sigma Aldrich), LiCl (99.99%, Sigma Aldrich), LiBr (99.99%, Alfa Aesar) or LiI (99.95%, Alfa Aesar), and ZrBr_4_ (99%, Alfa Aesar) or ZrI_4_ (99.5%, Stream) was ball‐milled at 600 rpm for 10 h in a ZrO_2_ vial with ZrO_2_ balls using Pulverisette 7PL (Fritsch GmbH). To prepare Li_6_PS_5_Cl, a stoichiometric mixture of Li_2_S (99.9%, Alfa Aesar), P_2_S_5_ (99%, Sigma Aldrich), and LiCl (99.99%, Sigma Aldrich) was ball‐milled under the same conditions as for Li_2_ZrCl_6‐y_X_y_ (X═Br, I), followed by annealing at 550 °C for 12 h under an Ar atmosphere. The resulting Li^+^ conductivity, measured by the AC impedance method using Ti|SE|Ti Li^+^‐blocking symmetric cells was 2.34 mS cm^−1^ at 30 °C.

### Material Characterization

Powder XRD patterns were collected using a Rigaku MiniFlex600 diffractometer with Cu K_α_ radiation (λ = 1.5406 Å). XRD cells containing hermetically sealed SE samples with a Be window were mounted on an XRD diffractometer and analyzed at 40 kV and 15 mA. X‐ray total‐scattering data were collected at beamline 28‐ID‐1 PDF at the National Synchrotron Light Source II (NSLSII) of Brookhaven National Laboratory, using an X‐ray energy of 74.5 keV (λ = 0.1665 Å). The prepared samples were loaded into polyimide tubes (Kapton) and hermetically sealed with epoxy resin. The 2D images were reduced to a 1D pattern with a CeO_2_ calibrant using Dioptas software.^[^ ^82^ ^]^ The PDF G(r) values were obtained from the Fourier transformation over a Q range of 0.1−20 Å^−1^ using xPDFsuite.^[^ ^83^ ^]^ Real‐space refinements were performed using a *P* $\bar{3}$ *m*1 structural model by adjusting the scale factor, lattice parameter, and atomic displacement parameters. A relaxed *P* $\bar{3}$ *m*1 fitting model was constructed for LZCI and LZCB based on a trigonal crystalline structure, assuming uniform substitution across all 6i anion sites (e.g., Cl, Br, and I) in the LZCX lattice. To account for local distortions, two out of three 6i anion sites were freely relaxed from the *P* $\bar{3}$ *m*1 symmetry for LZCB and LZCI having higher Br and I compositions. A strict fitting model was also employed for LZCB that shares the same atomic sites and atomic displacement parameters (ADPs) for Br and Cl atoms based on their similar ionic radii and the absence of first shell (Zr─Br and Zr─Cl) peak separations in PDF G(r). The low reliability for the Li_2_ZrCl_4_Br_2_ fitting result arises from weighted bond length differences between Zr─Cl and Zr─Br, which induce polyhedral distortions. Consequently, the short‐range PDF (1.5–10 Å) fitting results for Li_2_ZrCl_4_Br_2_ were more reliable, as they were less affected by lattice distortions. In contrast, as the LZCI shows distinct peak separation due to significant bond length difference between Zr─Cl and Zr─I, independent fitting parameters were used for Cl and I, except for Li_2_ZrCl_5.75_I_0.25_ where the same fitting parameters were shared owing to marginal amounts of I substitution. Zr K‐edge XAS measurements were conducted at the 7D, 8C, and 10C beamlines of the Pohang Accelerator Laboratory (PAL) using a Si (111) double‐crystal monochromator in the transmission and fluorescence modes. Energy calibration was performed using the reference spectra of the Zr metal foils. The XANES and EXAFS data were processed using the Demeter software package.^[^ ^84^ ^]^ The extracted EXAFS signal, k^3^χ(k), was Fourier transformed in the k‐range of 3.2–12.0 Å^−1^ and fitted in the R‐range of 1.25–3.50 Å. HRPD patterns were measured at the 9B beamline of the PAL using Si (111) double‐crystal monochromator (λ = 1.546 Å). The Rietveld refinement was performed to refine HRPD patterns using the FullProf software, excluding regions under 28.5 ° to avoid the influence of peaks for the air‐tight holders used to prevent air exposure.^[^ ^85^ ^]^ Ex situ XPS measurements were performed with a monochromatic Al Kα source (1486.6 eV) at 12 kV and 3 mA using K‐Alpha (Thermo Fisher Scientific). The samples were mounted on a sample holder in an Ar‐filled glove box and transferred to an XPS instrument without exposure to ambient air. Ar ion etching was performed at 200 eV for 60 s to remove surface contaminants.

### Electrochemical Characterization

Ionic conductivities were measured using the AC impedance method with ion‐blocking Ti*|*SE*|*Ti symmetric cells. Cold‐pressed pellets with diameters of 6 mm were prepared at 370 MPa. The EIS data of the cells were recorded under an external pressure of ≈70 MPa at an open‐circuit voltage with an amplitude of 10 mV and a frequency range of 10 mHz to 7 MHz using a VSP‐300 (Bio‐Logic); ten data points were recorded in each decade. For the all‐solid‐state half‐cells, Li‐In was used as the counter and reference electrodes. Li‐In powders with a nominal composition of Li_0.5_In prepared by ball‐milling In (Aldrich, 99%) and Li (FMC Lithium Corp.) were mixed with Li_6_PS_5_Cl powders in a weight ratio of 8:2. The all‐solid‐state cells were fabricated as follows. To prepare the LPSCl layer, LPSCl powder (150 mg, ≈600 µm) was pelletized at 100 MPa, and then a thinner layer of Li_2_ZrCl_6‐y_X_y_ (X═Br, I) (50 mg) was placed on the LPSCl layer and pelletized at 100 MPa. The composite working electrodes were prepared from a mixture of LiCoO_2_, Li_2_ZrCl_6‐y_X_y_, and Super C65 powders in a weight ratio of 70:30:3. Finally, the LiCoO_2_ electrodes (40–50 µm) and the Li‐In electrodes (≈130 µm) were attached on either side of the SE layers, and the whole assembly was pressed at 370 MPa. The all‐solid‐state cells were tested under an external pressure of ≈70 MPa. Glove box (MSIT‐24‐0100) at Institute for Battery Research Innovation (RS‐2023‐00261543) was used.

### Theoretical Calculations

First‐principles calculations were conducted using the Vienna Ab initio Simulation Package (VASP).^[^ ^86^ ^]^ The generalized gradient approximation (GGA) exchange correlation with the Perdew–Burke–Ernzerhof (PBE) functional^[^ ^87^ ^]^ was adopted, along with the projector‐augmented wave (PAW) method. A plane‐wave cut‐off energy of 520 eV was used, with the cell shape, cell volume, and atomic positions of each structure fully relaxed until the forces on each atom were below 0.05 eV Å^−1^. Ab initio molecular dynamics (AIMD) simulations were performed to calculate the Li^+^ diffusivity and reveal the migration mechanism. These simulations used an NVT ensemble with a Nose–Hoover thermostat having a period of 80 fs.^[^ ^88^ ^]^ The 1 × 1 × 2 supercells of Li_2_ZrCl_6_ and other structures have lattice parameters larger than 10 Å in each direction, and a Γ‐centered 1 × 1 × 1 k‐point grid was used. The heating process was executed for each supercell by increasing the temperature from 100 K to each holding temperature (550–750 K) over 2 ps. AIMD simulations were conducted with a 2 fs interval time step for 200 ps at different holding temperatures, and diffusivities (D) were determined by linear fitting of the mean square displacement (MSD) of lithium ions using the following Equations (1and 2):

(1) $$\mathrm{MSD}=\frac{1}{N}\sum_{i=1}^{N}{\left|r_{i}\left(t+\Delta t\right)-r_{i}\left(t\right)\right|}^{2}$$

(2) $$D=\frac{1}{2\mathrm{dtN}}\sum_{i=1}^{N}{\left|r_{i}\left(t+\Delta t\right)-r_{i}\left(t\right)\right|}^{2}$$

where r_i_ is the position of the i^th^ ion at time t, Δt is the time step, N is the number of Li atoms in the supercell structure, and d is the dimensionality factor. The MSD and diffusivities were analyzed using a diffusion analyzer module^[^ ^89^ ^]^ in PyMatgen.^[^ ^90^ ^]^ Diffusion channel size analysis based on the Li‐ion trajectories generated from AIMD simulations was performed using the topological analysis package Zeo^++^.^[^ ^91^ ^]^ Initial and final configurations in AIMD simulations for Li^+^ diffusion of all structures are presented in Figures S31 and S32 (Supporting Information). For plane‐wave‐based DFT calculations, it is essential to clearly define the structure, particularly when dealing with partial occupations. Detailed explanations of the structure generation methodology and simulation results were provided, with a schematic of the overall process presented in Figure S33 (Supporting Information). Initially, the crystal structure of Li_3_ErCl_6_ was obtained from the Materials Project database (mp‐676361). Er was substituted with Zr, along with partial occupancy of Li (Li_18_Er_6_Cl_36_ [Li_3_ErCl_6_] → Li_12_Zr_6_Cl_36_ [Li_2_ZrCl_6_]). A 1 × 1 × 2 supercell (a = b = 11.00960 Å, c = 12.04801 Å) was employed for all DFT simulations. For partially occupied structures (Li: 12/18), the enumeration method was used to systematically generate all possible atomic arrangements by exploring various combinations of partially occupied sites. This approach leverages symmetry to minimize the number of unique configurations, ensuring only symmetrically distinct arrangements wre evaluated. The Python package “Python Materials Genomics (pymatgen)” enables efficient enumeration, reducing computational expense by ensuring only distinct atomic configurations. Thirty configurations with the lowest Ewald summation energies were initially generated, representing possible atomic arrangements based on partial occupancies. These candidate structures were then subjected to full structural relaxation using the consistent DFT parameters, with the lowest total energy configuration selected as the most stable structure. Finally, the M2F structure of Li_2_ZrCl_6_ structure was constructed. In the M2F Li_2_ZrCl_6_ structure, Zr metal atoms at M2 site were moved to M3 site (one side for 1SD and both side for M3F), and anion substitutions were performed according to the desired substitution level (3 of 36 for Li_2_ZrCl_5.5_X_0.5_ and 6 of 36 for Li_2_ZrCl_5_X). For Br and I substitutions, an enumeration were conducted by selecting a specific number of sites based on the substitution ratio among the total 36 Cl anion sites. Since the stable lithium sites could vary depending on the positions of the metal atoms and anions, enumeration was repeated to identify potential lithium insertion sites. To model additional lithium sites in each of the fifteen Li_2_ZrX_6_ structures (3 crystal structures (M2F, 1SD, M3F) and 5 compositions (LZC, LZCB0.5, LZCB1, LZCI0.5, LZCI1)), the TopographyAnalyzer code from the pymatgen package were employed. This allowed us to preselect topologically favorable sites for lithium insertion. From this preselection, 12 Li atoms out of were enumerated 30 plausible Li octahedral sites for M2F, 12 out of 28 Li octahedral sites for 1SD, and 12 out of 26 Li octahedral sites for M3F in each of the 5 compositions.

## Conflict of Interest

The authors declare no conflict of interest.

## Supporting information



Supporting Information

## Data Availability

The data that support the findings of this study are available in the supplementary material of this article.
