# Supplementary material for: Mechanism of Contrasting Ionic Conductivities in Li2ZrCl6 via I and Br Substitution
Source: Small. 2025 Sep 2;21(41):e05926. doi: 10.1002/smll.202505926 (PMC12530019; doi:10.1002/smll.202505926)
Supplement: Supplementary file 1 — Supporting Information [file SMLL-21-e05926-s001.docx]

**Mechanism of Contrasting Ionic Conductivities in Li_2_ZrCl_6_ via I and Br Substitution**

**Table of Contents**

**1. Supplementary Notes**

**Note S1: Distinct M2-M3 disorder between Br- and I-substituted LZCX**

**Note S2. Anionic hinderance effect**

**Note S3: Electronic Structure Analysis of Lattice Polarization and Its Influence on Li^+^ Conductivity**

**Note S4: The occurrence of optimal Li^+^ conductivity at specific substitution levels**

**2. Supplementary Figures S1−S37**

**3. Supplementary Tables S1−S22**

**4. References**

**1. Supplementary Notes**

**Note S1: Distinct M2-M3 disorder between Br- and I-substituted LZCX**

To investigate the origin of the distinct M2-M3 disorder between Br- and I-substituted LZCX, we calculated the energy above hull (E_hull_) to computationally evaluate the structural stability influenced by M2-M3 site disorder. These results are summarized in Table S3. For the Br-substituted structures, the 1SD configuration exhibited the highest stability, while the configurations with M2F and M3F sites showed comparable E_hull_ values. The preference of the 1SD configuration can be attributed to the limited lattice expansion relative to the size of the anion. This constrained expansion allows Zr atoms to simultaneously occupy M2 and M3 sites, effectively alleviating lattice strain and enhancing structural stability.

While the 1SD configuration is preferred in LZCB, in the I-substituted structures, M2F-Li_2_ZrCl_5.5_I_0.5_ and 1SD-Li_2_ZrCl_5_I were identified as the most favorable structures, with marginal energy differences between M2F and 1SD for Li_2_ZrCl_5_I. Notably, all M3F configurations in the I-substituted structures were significantly less stable compared to the M2F and 1SD configurations. In the M2F structure, the M1 and M3 sites share the same layer, and the M2 site supports the intermediate layer. The incorporation of the large I^-^ anion induces considerable structural expansion; however, a completely vacant M2 site leaves the intermediate layer unsupported by metal elements. When a metal occupies the intermediate layer, as in the M2F structure, it generates electrostatic repulsion with adjacent Zr atoms along the *c*-axis, serving as a pillar to sustain the inter-slab distance. Conversely, the M3F configuration suffers from increased asymmetric stress distribution, resulting in reduced structural stability. Additionally, when a metal element is present in the intermediate layer, it interacts with surrounding ions to minimize energy, thereby enhancing structural stability. These interactions help maintain overall system stability during volumetric expansion, a balance that the M3F structure fails to achieve.

Compared to Br-substituted structures, I-substituted structures experience more pronounced volume expansion (**Figure 6** and Table S4). This greater expansion, combined with the absence of a stabilizing framework in the M3F structure to support the intermediate layer (M2 site), accounts for the distinct stabilities observed among the three structural forms.

**Note S2. Anionic hinderance effect**

The AIMD simulation results at 600 K for up to 300 ps indicate that Br-substituted M3F structures show significantly reduced conduction along the *ab*-plane compared to the unsubstituted structure (M3F-LZC) (Figure 5a, Figure S19c, S24a, and S24b). Particularly, for M3F-Li_2_ZrCl_5_Br, no conduction is observed along the *ab*-plane. Although the lattice of the Br-substituted structures exhibits clear expansion (Table S4), channel size analysis reveals a lower median value and an increased number of smaller channels (Figure S21c). This indicates that the conduction hindrance effect from the large anion outweighs the benefits of the lattice expansion, resulting in a reduction in the channel size for lithium conduction (Figure S26a and b). Specifically, the *c*-axis increases by only 0.06 Å in M3F-Li_2_ZrCl_5.5_Br_0.5_ and by 0.13 Å in M3F-Li_2_ZrCl_5_Br compared to the M3F-Li_2_ZrCl_6_, despite the anion size increasing by 0.15 Å (Cl^-^: 1.81 Å and Br^-^: 1.96 Å) (Table S4). This mismatch results in blockage of the *ab*-plane. In other words, the elongation of Zr-X bond caused by the larger anion has a greater impact on obstructing conduction channels than on increasing the lattice volume, indicating that Br substitution inherently deteriorates ion conduction within the M3F structure.

Similarly, in the 1SD structure, Br substitution increases lattice volume without significantly altering channel size, suggesting a comparable effect to that observed in the M3F structure. (Table S4 and Figure S21b) A comparable anionic hindrance effect is also evident in the I-substituted M2F structures (Figure S26c and d). Despite the larger lattice volume of the M2F Li_2_ZrCl_5_I_1_ structure (Table S4), its channel size distribution is narrower than in Li_2_ZrCl_5.5_I_0.5_ (Figure S21a). Furthermore, the Li probability density in I-substituted M2F structures at 600 K reveals a discontinuity near the I anion (Figure S26c, and d), providing further evidence of the hindrance to lithium conduction by the larger anions.

**Note S3: Electronic Structure Analysis of Lattice Polarization and Its Influence on Li^+^ Conductivity**

To address the potential role of lattice polarization in influencing local disorder and ionic conductivity, we performed additional electronic structure analyses, including electrostatic local potential (LOCPOT) and electron localization function (ELF) evaluations. A highly polarizable lattice is expected to exhibit a significant breakdown in the symmetry of the electron cloud distribution, leading to overall electron delocalization. This effect can be visualized through three-dimensional (3D) ELF iso-surfaces, where less localized electron densities reflect enhanced polarizability. From the electrostatic potential perspective, a lattice exhibiting large variations in the local potential landscape indicates a higher degree of polarizability, as it reflects the ease with which the electronic environment responds to structural perturbations or ion displacements.

In Figure S34, 3D ELF maps (isosurface value = 0.60) show increasing charge delocalization around halide ions with higher Br/I content. Also, planar-averaged electrostatic potential profiles extracted from local potential along the crystallographic a axis and c axis, illustrating the progressive flattening of potential wells and enhanced lattice polarizability upon halide substitution. For LZC, the local potential profile displays two distinct peaks, whereas increased polarization upon Br⁻ and I⁻ substitution leads to the merging and normalization of these peaks, supporting the presence of stronger lattice polarization effects. With increasing substitution of Br⁻ and I⁻, the electrostatic potential profile exhibits noticeable asymmetric features with delocalization by increased difference of potential minimum, indicating the formation of a more polarizable lattice. This is consistent with the increased spatial spread of the ELF iso-surface around halide anions, reflecting enhanced electron cloud distortion under the local field. Such delocalization implies softening of the local lattice environment, which could introduce local strain or lattice asymmetry. Notably, this behavior is consistently observed regardless of the type of structural disorder, whether in the form of M2/M3 disorder (1SD or M3F), which highlights that halide substitution effectively amplifies lattice polarization across different configurations (Figure S35).

While Br^-^ exhibits moderate polarizability, I^-^ induces significantly enhanced local potential distortion and electron delocalization. This behavior originates from the larger ionic radius and more diffuse electron cloud of I^-^, arising from its higher principal quantum number and the associated loosely bound outer electron shell. As a result, the lattice environment surrounding I^-^ becomes more susceptible to polarization under local electrostatic fields, thereby amplifying structural asymmetry and potential fluctuations. To further assess local strain induced by lattice polarization, we analyzed the variation in local bond lengths with distortion index of ZrX_6_ polyhedra (Figure S36). The iodine substitution induces greater lattice distortion compared to bromine substitution, and the extent of distortion increases with higher substitution ratios. This trend is consistent with enhanced lattice polarizability, suggesting a direct correlation between increased polarizability and structural asymmetry.

Furthermore, simulated radial distribution (RDF) analysis supports this interpretation, showing broadened Li–Cl peak profiles as the Br⁻/I⁻ content increases, suggesting increased local structural disorder (Figure S37). The broadening of the Li–Cl RDF peak reflects increased variability in Li–X distances, which arises from the more asymmetric and strained local environments due to enhanced polarizability. The flattened electrostatic potential landscape, induced by halide polarization, reduces the energy barriers for Li⁺ hopping between adjacent sites. This trend aligns with the increased ionic conductivity observed in our results. Collectively, our theoretical results support the hypothesis that lattice polarization induced by heavier halide substitution contributes not only to local strain but also to improved ionic conductivity.

Importantly, we note that when the overall host structure is retained, the substitution of larger and more polarizable halide anions can lead to enhanced ionic conductivity. This is attributed to the induced lattice polarization, which effectively lowers Li^+^ migration barriers without altering the space group (Table S22).

In summary, our combined electrostatic potential analysis, ELF visualization, polyhedral distortion metrics, and RDF evaluation collectively demonstrate that halide substitution⎯particularly with I⁻⎯induces significant lattice polarization. This enhanced polarizability gives rise to local structural asymmetry, electron delocalization, and potential flattening, which together reduce Li⁺ migration barriers. These findings highlight lattice polarization as a key physical origin underlying the observed improvement in ionic conductivity upon Br⁻ and I⁻ incorporation and confirm that this mechanism is consistently active across different structural configurations.

**Note S4: The occurrence of optimal Li^+^ conductivity at specific substitution levels**

As shown in Figure S21a for the M2F structure and Figure S21b for the 1SD structure, the channel size distribution in Li_2_ZrCl_5.5_I_0.5_ is broader than that in Li_2_ZrCl_5_I_1_. The narrower channel size distribution in the I-richer composition (Li_2_ZrCl_5_I_1_), despite its larger lattice volume (M2F structure, Table S18), is driven by spatial hindrance caused by the larger anion in the conduction pathways. Additionally, the Li probability density in I-substituted M2F structures at 600 K shows a discontinuity near the I anion (Figure S26c and d). These results suggest the existence of an optimal balance between the trade-off effects of anionic conduction blocking and lattice expansion. Specifically, the hindrance effect is more pronounced in Li_2_ZrCl_5_I_1_ compared to Li_2_ZrCl_5.5_I_0.5_. Furthermore, the M3 site occupancy decreases with increasing I substitution (**Figure 3e and** Table S16), which is favorable for Li conduction. Notably, LZCI exhibits greater structural instability in the M3F structure compared to Br-substituted and bare LZC structures (Table S3). This reduced M3 site occupancy in LZCI, contrasting with LZCB, likely also contributes to the increased ionic conductivity.

From these perspectives, the substitution of up to 0.5 equivalents of I is likely the optimal point for balancing lattice expansion (i.e., the combined effects of large I incorporation and reduced M3 occupancy) and anionic hindrance. In our AIMD simulations (**Figure 6a** and **6b**), both the M2F and 1SD structures of Li_2_ZrCl_5.5_I_0.5_ exhibit higher diffusivity compared with Li_2_ZrCl_5.5_I. This trend aligns with the observed decrease in experimental conductivity for Li_2_ZrCl_5_I_1_ (Figure 1c).

For Br-substitution, as the substitution ratio increases, the occupancy of M3 increases. Therefore, it is necessary to investigate the phenomena occurring within the M3F structure. Indeed, the Br-substituted M3F structures have significantly lower diffusivity than that of M3F-LZC structure (**Figure 6c**). The AIMD simulation results at 600 K for up to 300 ps indicate that Br-substituted M3F structures show significantly reduced conduction along the *ab*-plane compared to the unsubstituted structure (M3F-LZC) (Figure S19c, S24a, S24b). Particularly, for M3F-Li_2_ZrCl_5_Br, no conduction is observed along the *ab*-plane. Although the lattice of the Br-substituted structures exhibits clear expansion (Table S4), channel size analysis reveals a lower median value and an increased number of smaller channels (Figure S21c). This indicates that the conduction hindrance effect from the large anion outweighs the benefits of the lattice expansion, resulting in a reduction in the channel size for lithium conduction (Figure S26a and b). Specifically, the *c*-axis increases by only 0.06 Å in Li_2_ZrCl_5.5_Br_0.5_ and by 0.13 Å in Li_2_ZrCl_5_Br compared to the Li_2_ZrCl_6_, despite the anion size increasing by 0.15 Å (Cl^-^: 1.81 Å and Br^-^: 1.96 Å) (Table S4). This mismatch results in blockage of the *ab*-plane. In other words, the elongation of Zr-X bond caused by the larger anion has a greater impact on obstructing conduction channels than on increasing the lattice volume, indicating that Br substitution inherently deteriorates ion conduction within the M3F structure.

The decreased conductivity in Br-substituted structures is influenced not only by the aforementioned anionic hinderance effect but also by the increased M3F occupancy. Experimentally, higher Br substitution ratio leads to greater M3 occupancy, shifting the structure close to the M3F configuration (**Figure 3e** and Table S16). Notably, all M3F structures exhibit significantly lower ionic conductivity compared to their 1SD and M2F counterparts. (**Figure 6c**) For Li_2_ZrCl_5.5_Br_0.5_, its comparable conductivity relative to LZC can thus be attributed to comparatively lower M3 occupancy compared to Li_2_ZrCl_5_Br. Additionally, the lower concentration of Br atoms helps to mitigate the anionic hindrance effect.

**2. Supplementary Figures**

**
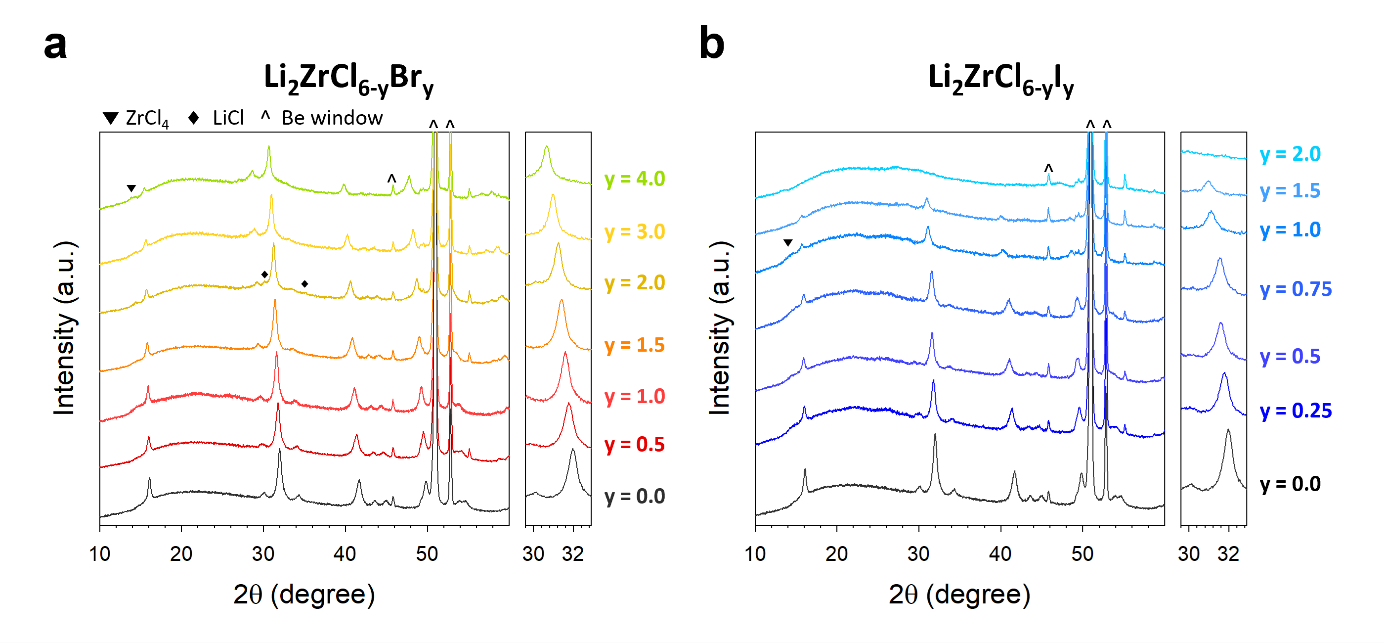
**

**Figure S1. a, b,** XRD patterns of Li_2_ZrCl_6-y_Br_y_ (**a**) and Li_2_ZrCl_6-y_I_y_ (**b**) (y = 0–2.0).

**
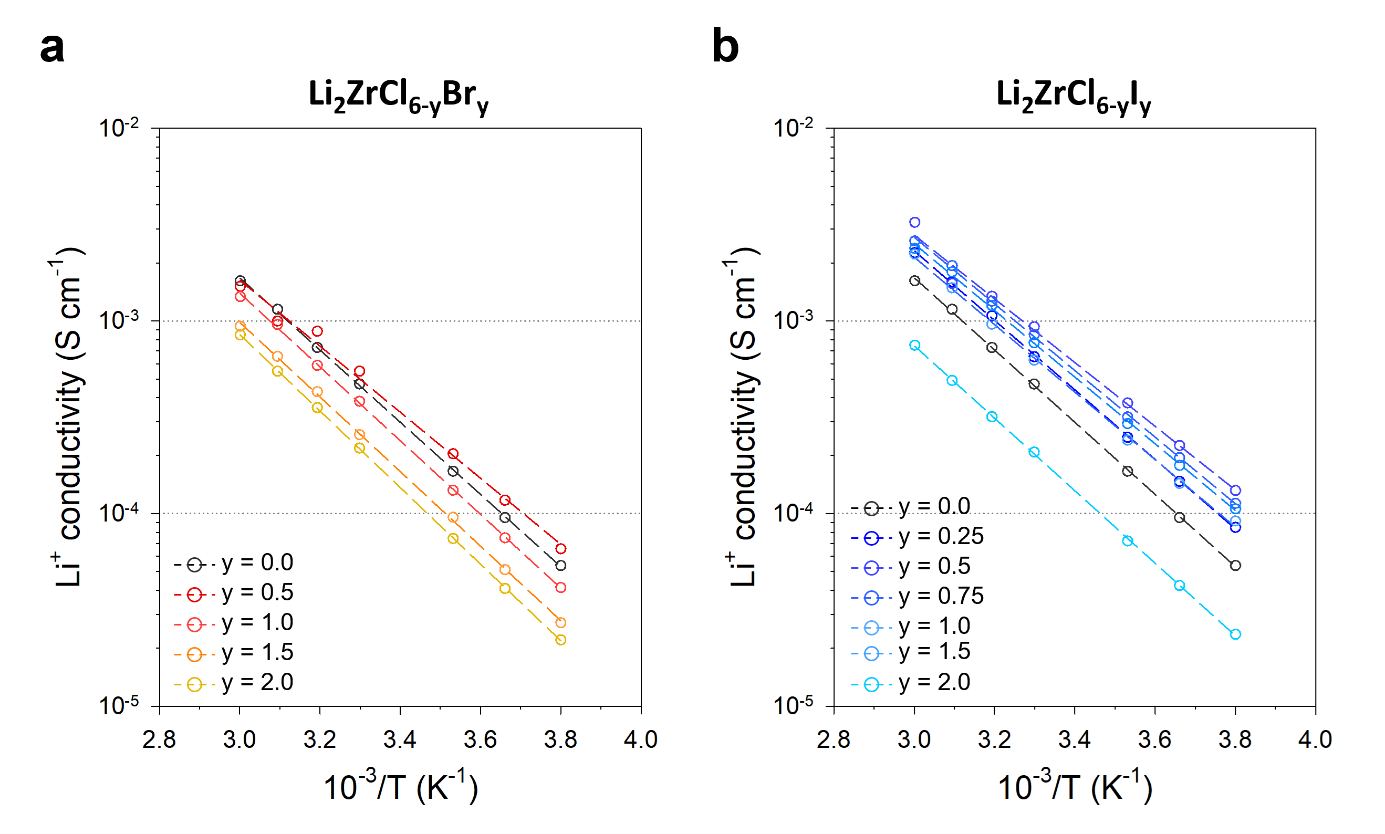
**

**Figure S2. a, b,** Arrhenius plots of ionic conductivities for Li_2_ZrCl_6-y_Br_y_ (**a**) and Li_2_ZrCl_6-y_I_y_ (**b**) (y = 0–2.0).

**
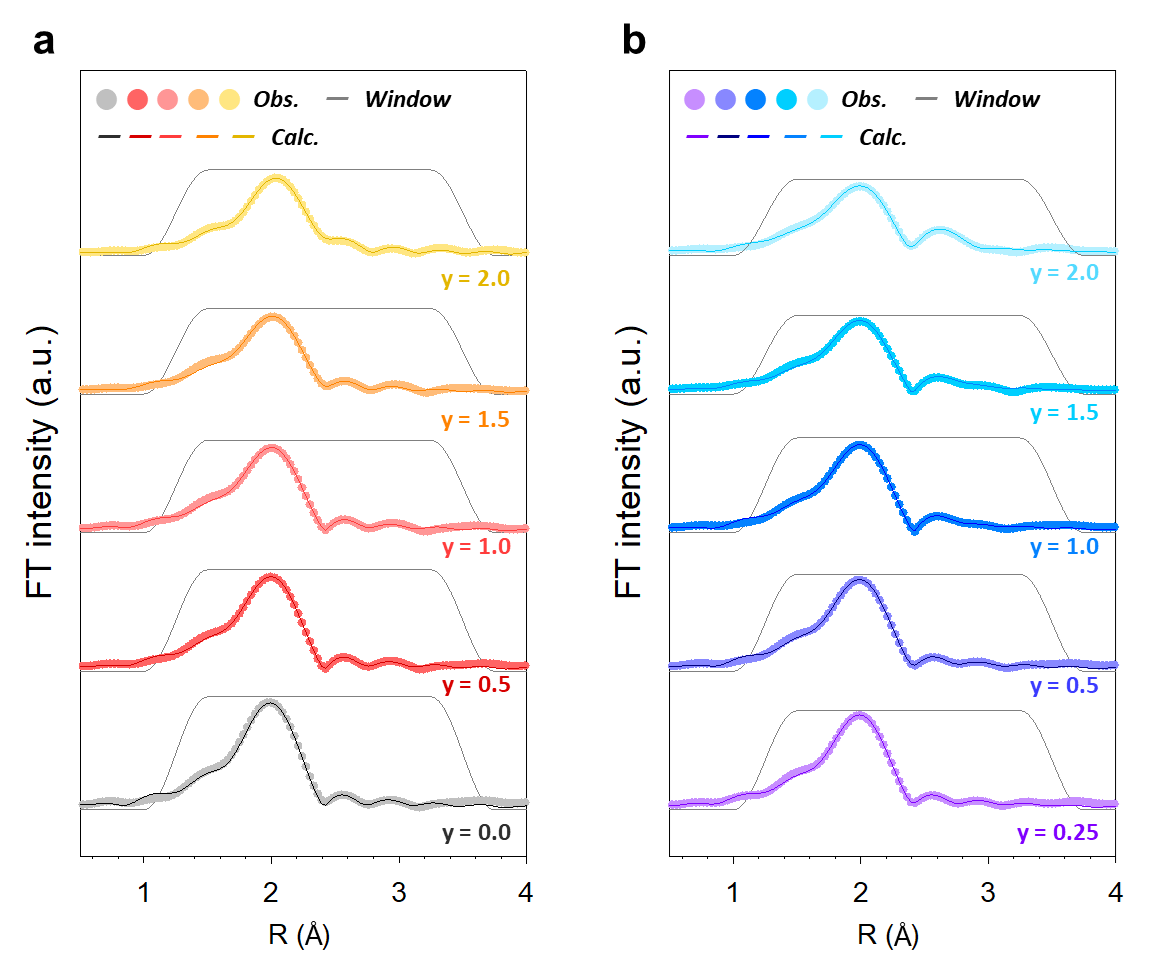
**

**Figure S3. a, b,** Zr K-edge EXAFS results and first-shell fitting curves of Li_2_ZrCl_6-y_Br_y_ (**a**) and Li_2_ZrCl_6-y_I_y_ (**b**).

**
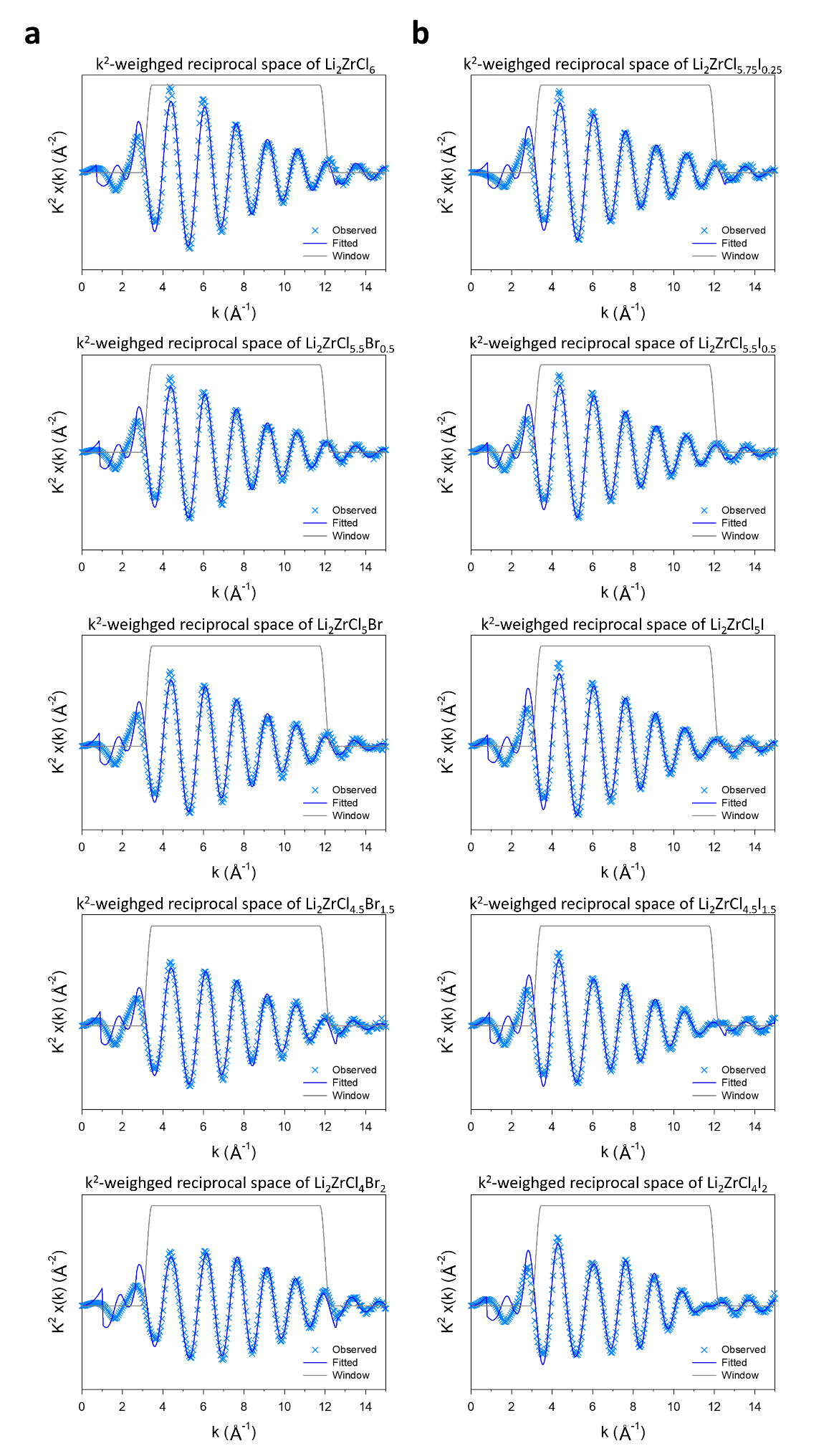
**

**Figure S4. a, b,** Fitted k^2^-weighged reciprocal space data of LZC, LZCB (**a**) and LZCI (**b**).


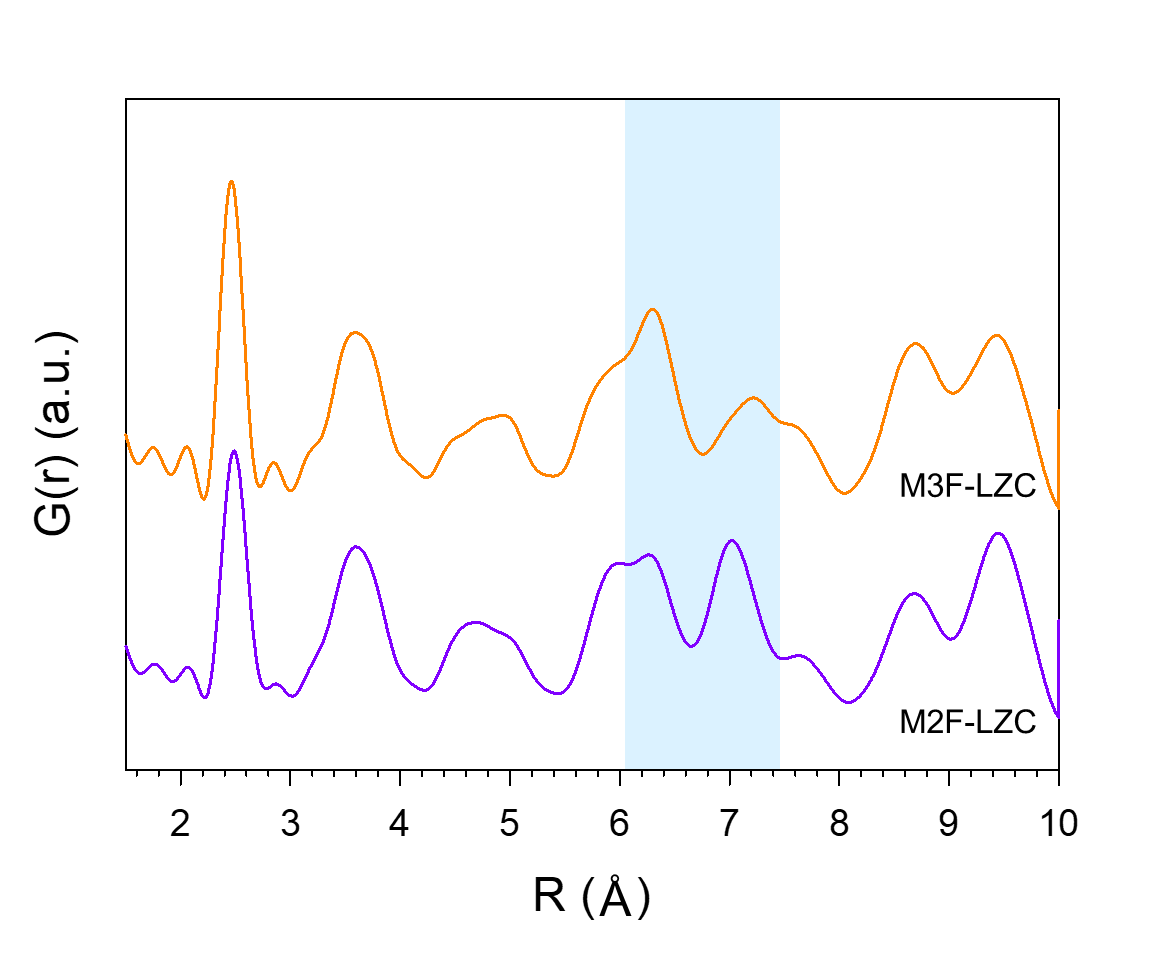


**Figure S5.** PDF calculation results for M2-fully occupied Li_2_ZrCl_6_ (M2F-LZC) and M3-fully occupied Li_2_ZrCl_6_ (M3F-LZC).

**
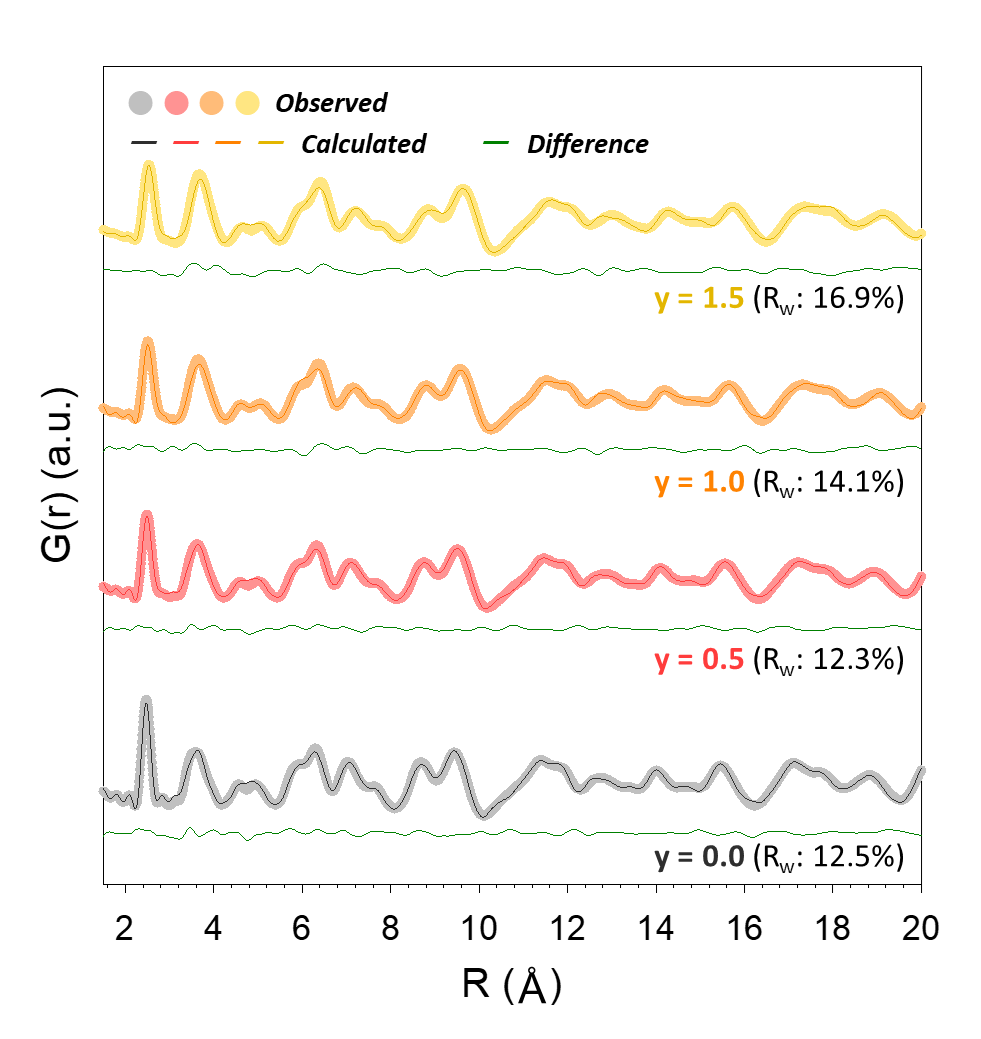
**

**Figure S6.** PDF G(r) fitting results for Li_2_ZrCl_6-y_Br_y_ (0 ≤ y ≤ 1.5) in the R range of 1.5 – 20 Å.

**
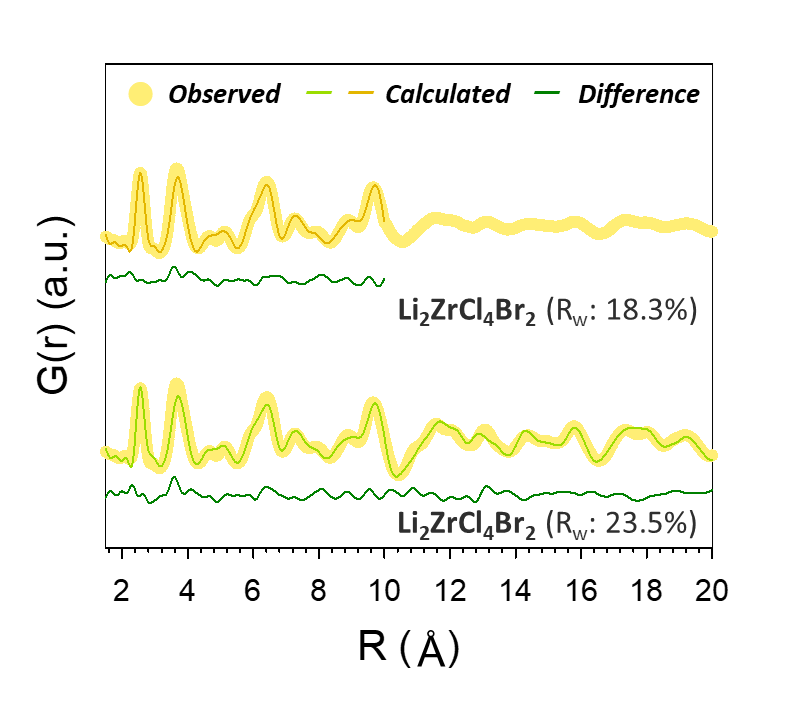
**

**Figure S7.** PDF G(r) fitting results for Li_2_ZrCl_4_Br_2_ in the R range of 1.5–10 Å and 1.5–20 Å.

**
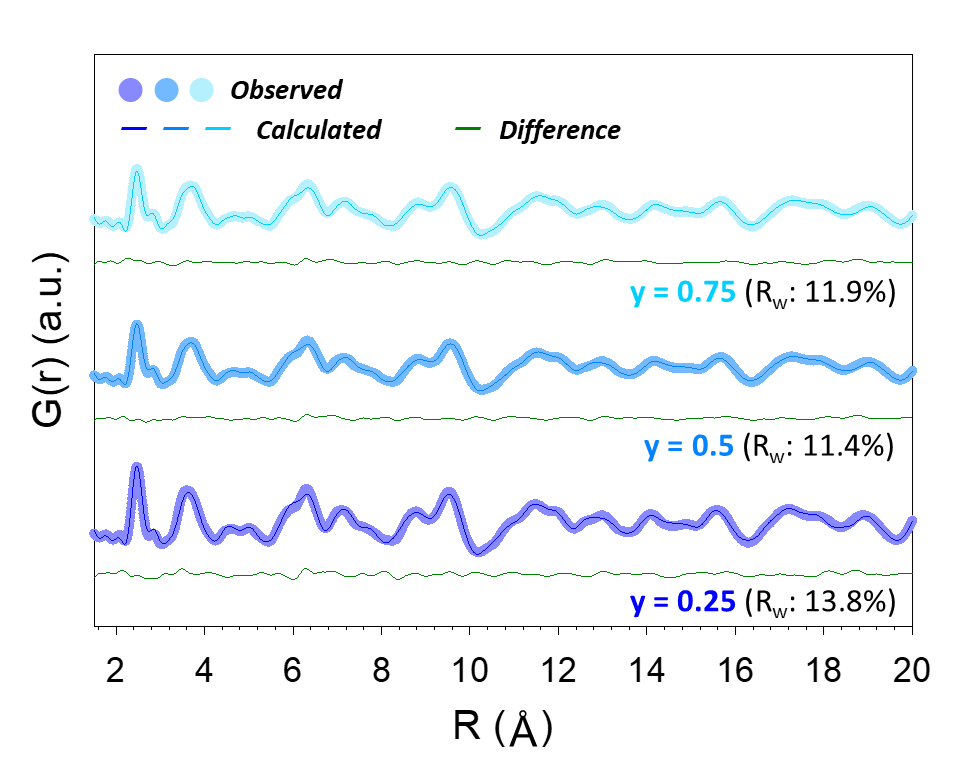
**

**Figure S8.** PDF G(r) fitting results for Li_2_ZrCl_6-y_I_y_ (0.25 ≤ y ≤ 0.75) in the R range of 1.5–20 Å.

**
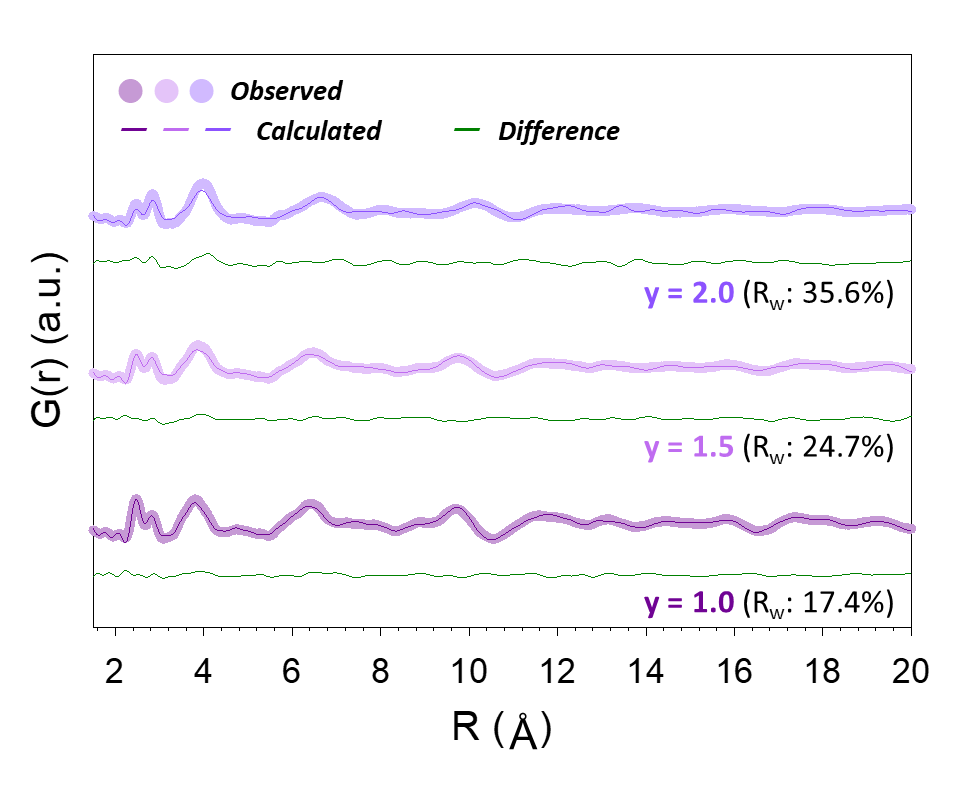
**

**Figure S9.** PDF G(r) fitting results for Li_2_ZrCl_6-y_I_y_ (1.0 ≤ y ≤ 2.0) in the R range of 1.5–20 Å.

**
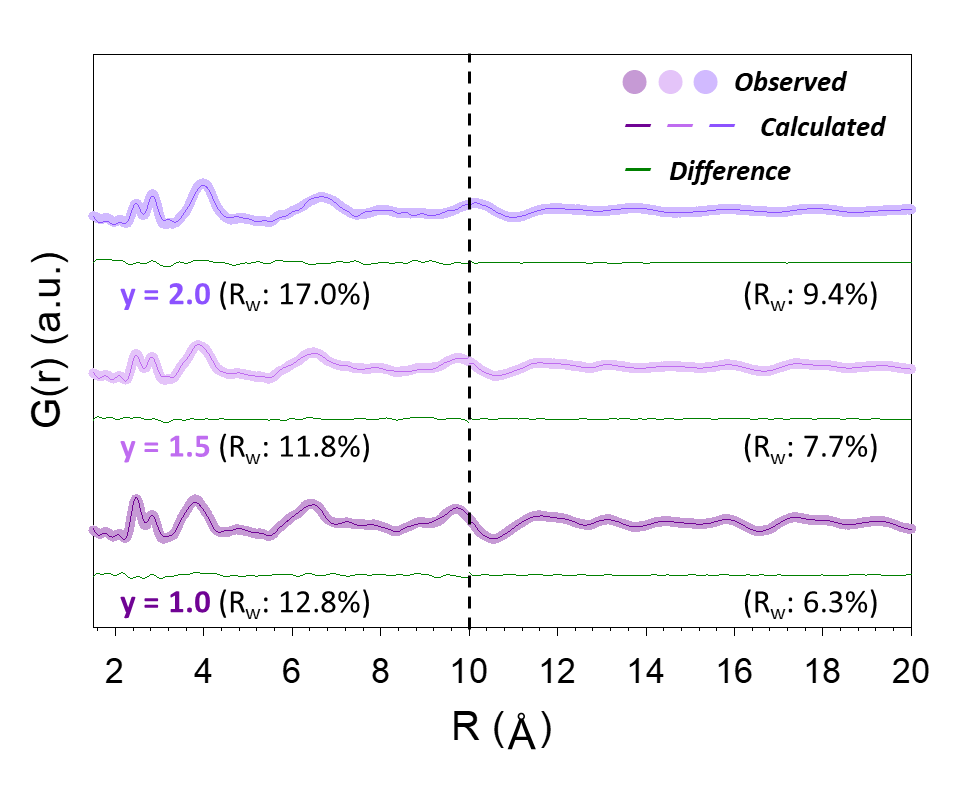
**

**Figure S10.** PDF G(r) fitting results for Li_2_ZrCl_6-y_I_y_ (1.0 ≤ y ≤ 2.0) in the R range of 1.5–10 Å and 10–20 Å.


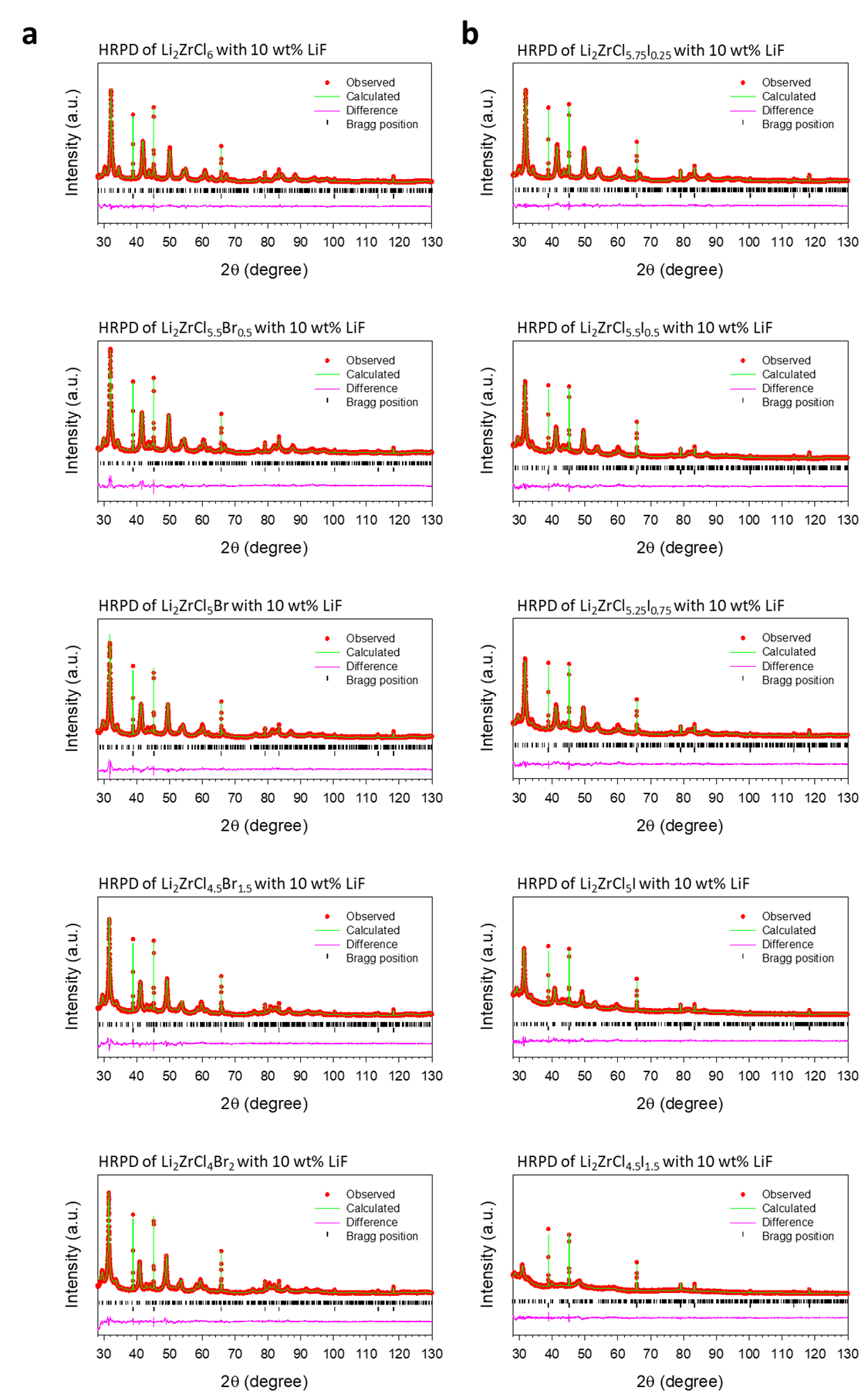


**Figure S11.** **a, b,** Observed and calculated HRPD Rietveld refinement profiles for the mixtures of SE sample and LiF in a weight ratio of 10:1 for Li_2_ZrCl_6-y_Br_y_ (0 ≤ y ≤ 2.0) (**a**) and Li_2_ZrCl_6-y_I_y_ (0.25 ≤ y ≤ 1.5) (**b**). The fitting results are presented in Table S5−14.

**Figure S12**. Observed and calculated HRPD Rietveld refinement profiles for the mixtures of SE sample and LiF in a weight ratio of 10:1 for Li_2_ZrCl_4_I_2_. The fitting results are presented in Table S15.

**
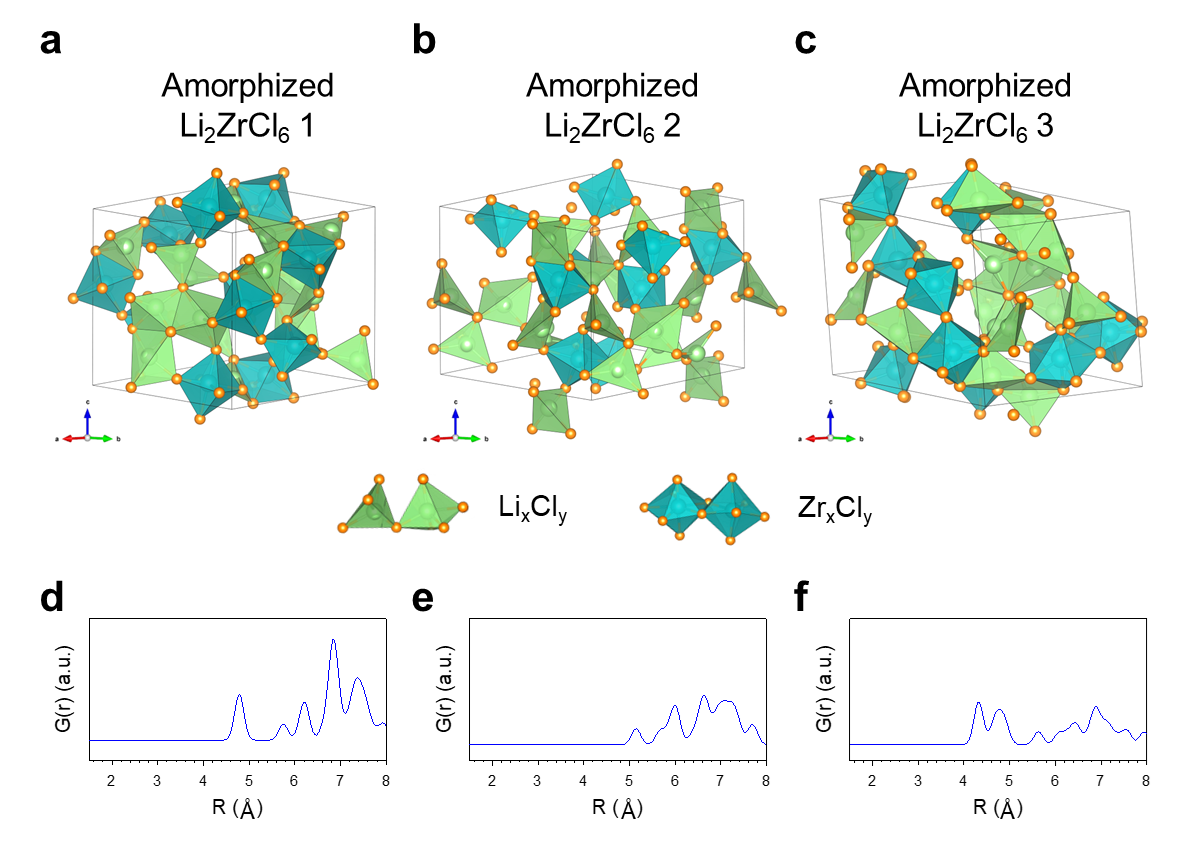
**

**Figure S13. a-c**, Amorphous structures of Li_2_ZrCl_6_ generated by melt-quenching (MQ) AIMD method. **d-f**, Simulated RDF of Zr-Zr distance in amorphized Li_2_ZrCl_6_.

**
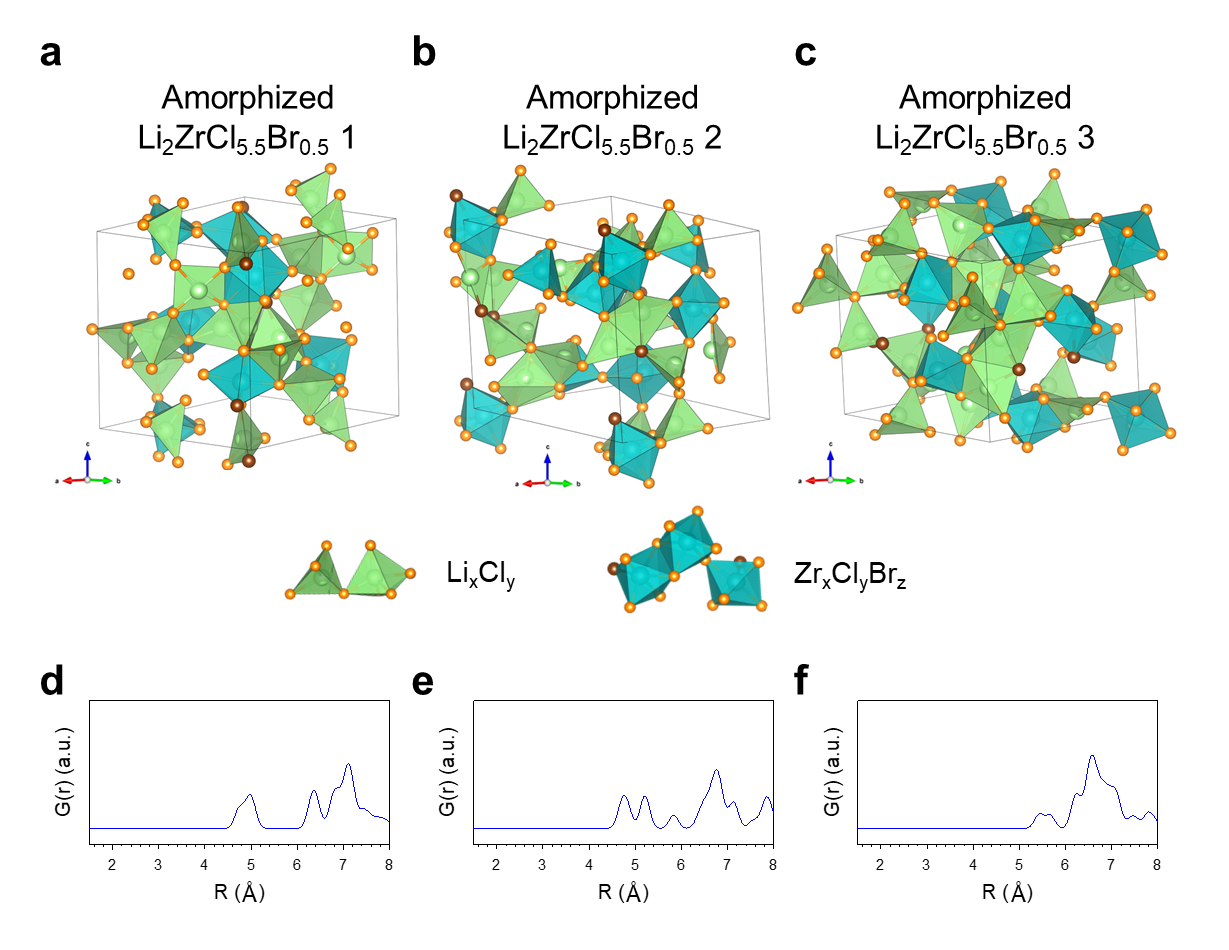
**

**Figure S14. a-c**, Amorphous structures of Li_2_ZrCl_5.5_Br_0.5_ generated by Melt-quenching (MQ) AIMD method. **d-f**, Simulated RDF of Zr-Zr distance in amorphized Li_2_ZrCl_5.5_Br_0.5_.


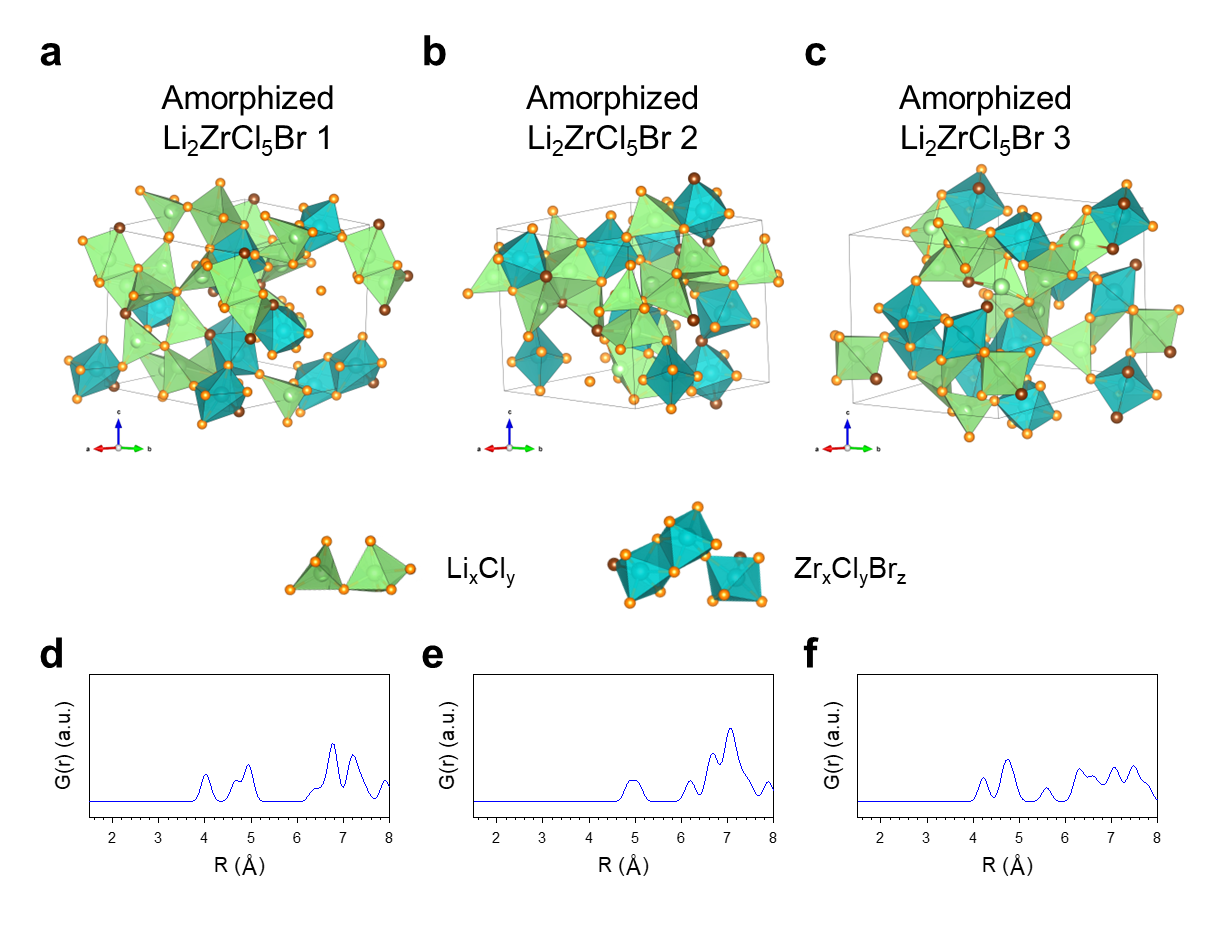


**Figure S15. a-c**, Amorphous structures of Li_2_ZrCl_5_Br generated by Melt-quenching (MQ) AIMD method. **d-f**, Simulated RDF of Zr-Zr distance in amorphized Li_2_ZrCl_5_Br.

**
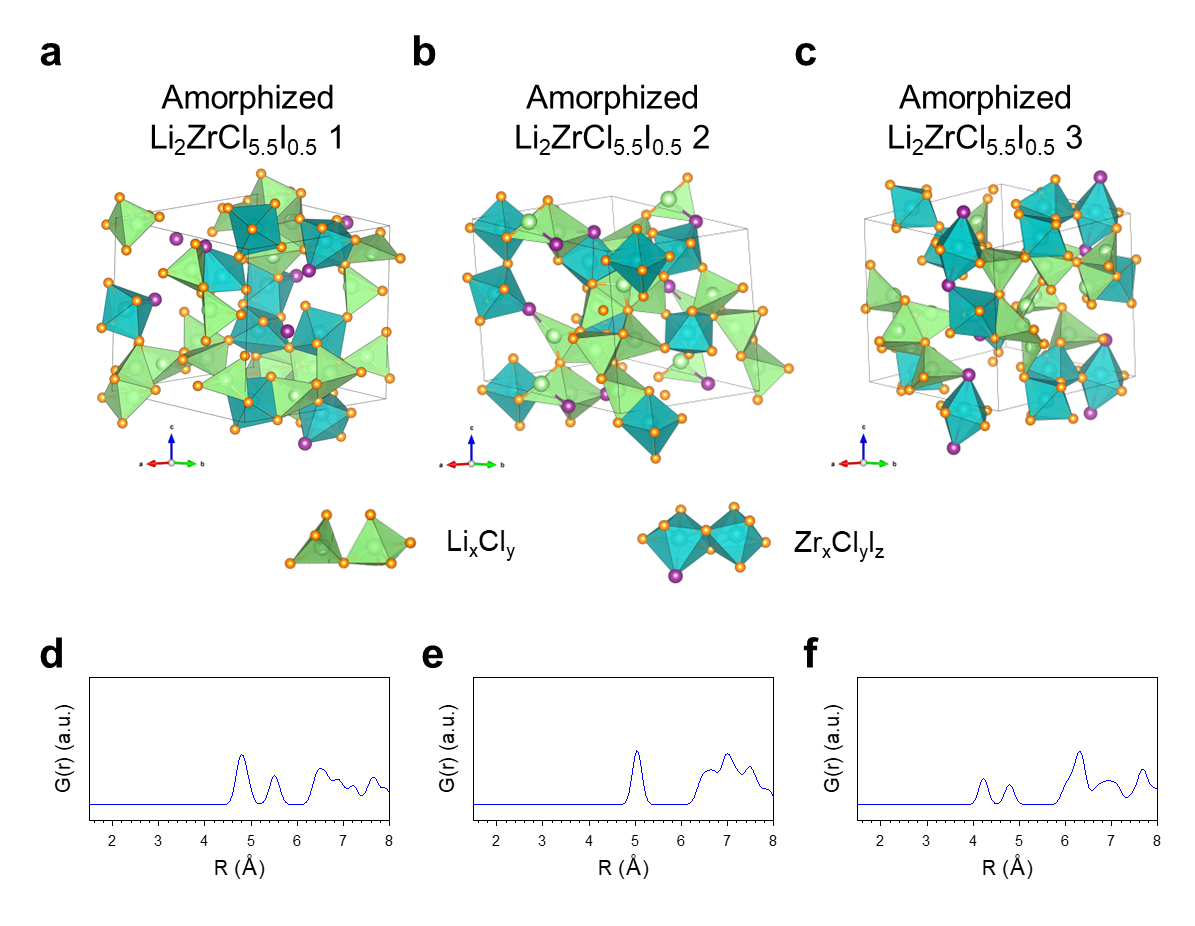
**

**Figure S16. a-c**, Amorphous structures of Li_2_ZrCl_5.5_I_0.5_ generated by Melt-quenching (MQ) AIMD method. **d-f**, Simulated RDF of Zr-Zr distance in amorphized Li_2_ZrCl_5.5_I_0.5_.


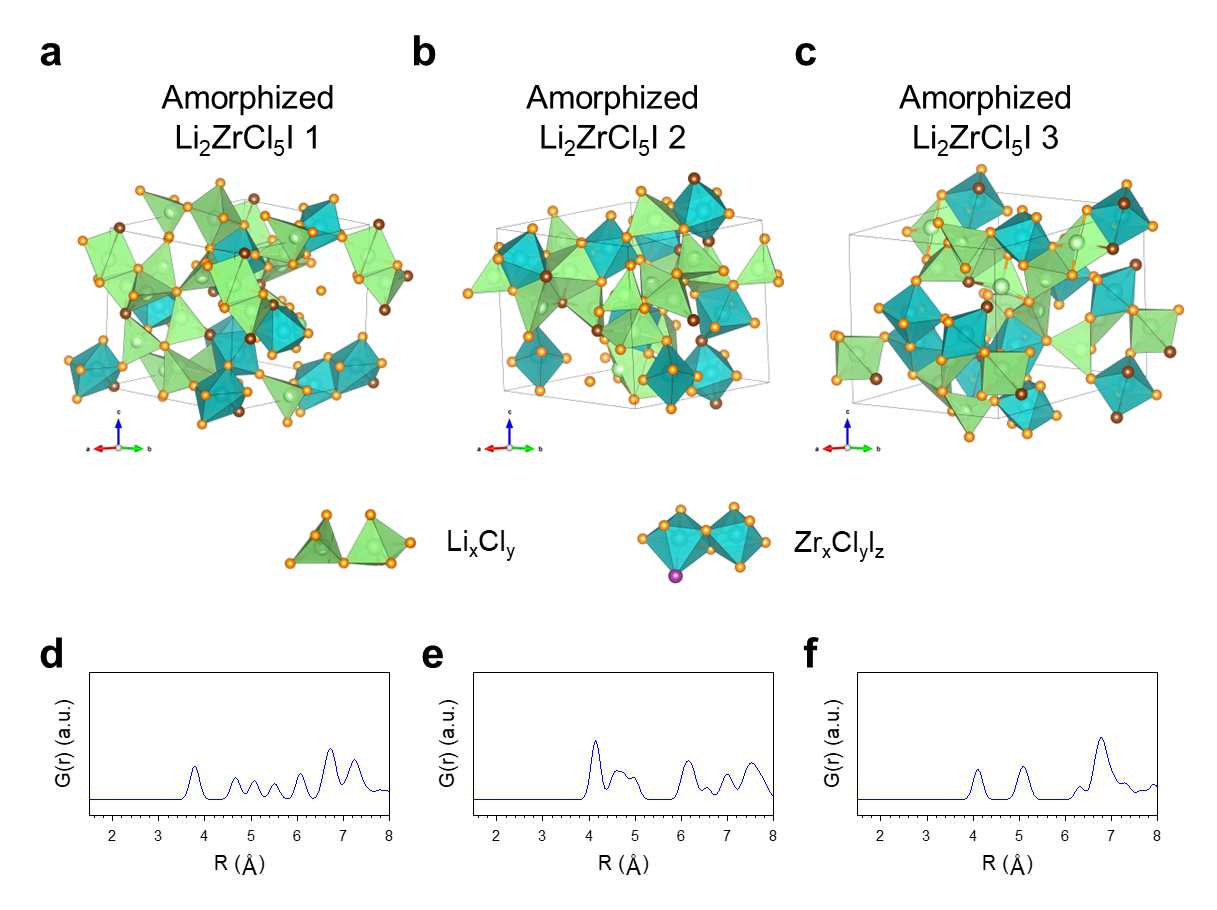


**Figure S17. a-c**, Amorphous structures of Li_2_ZrCl_5_I generated by Melt-quenching (MQ) AIMD method. **d-f**, Simulated RDF of Zr-Zr distance in amorphized Li_2_ZrCl_5_I.


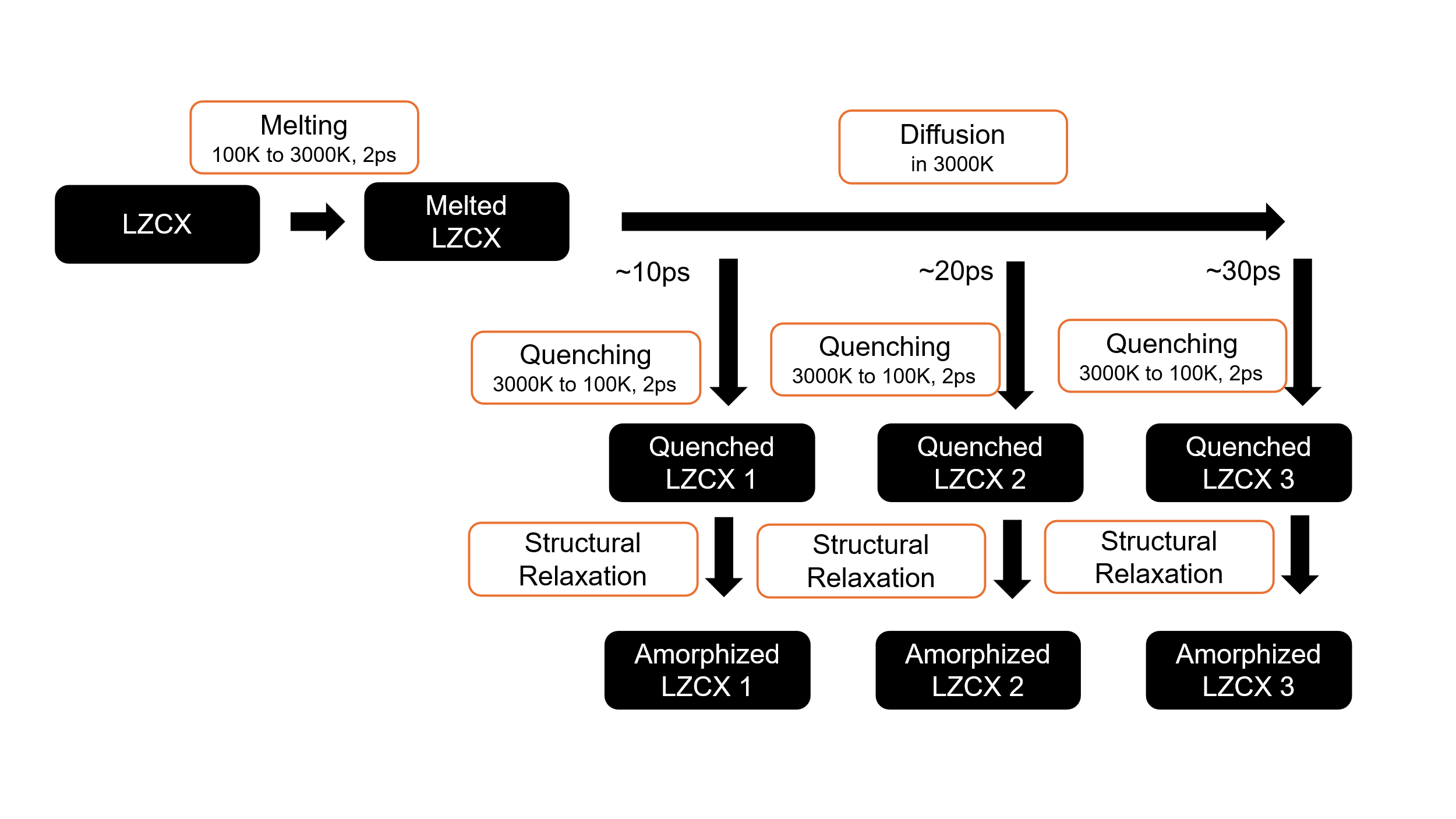


**Figure S18.** Workflow of melt-quenching AIMD method.

**
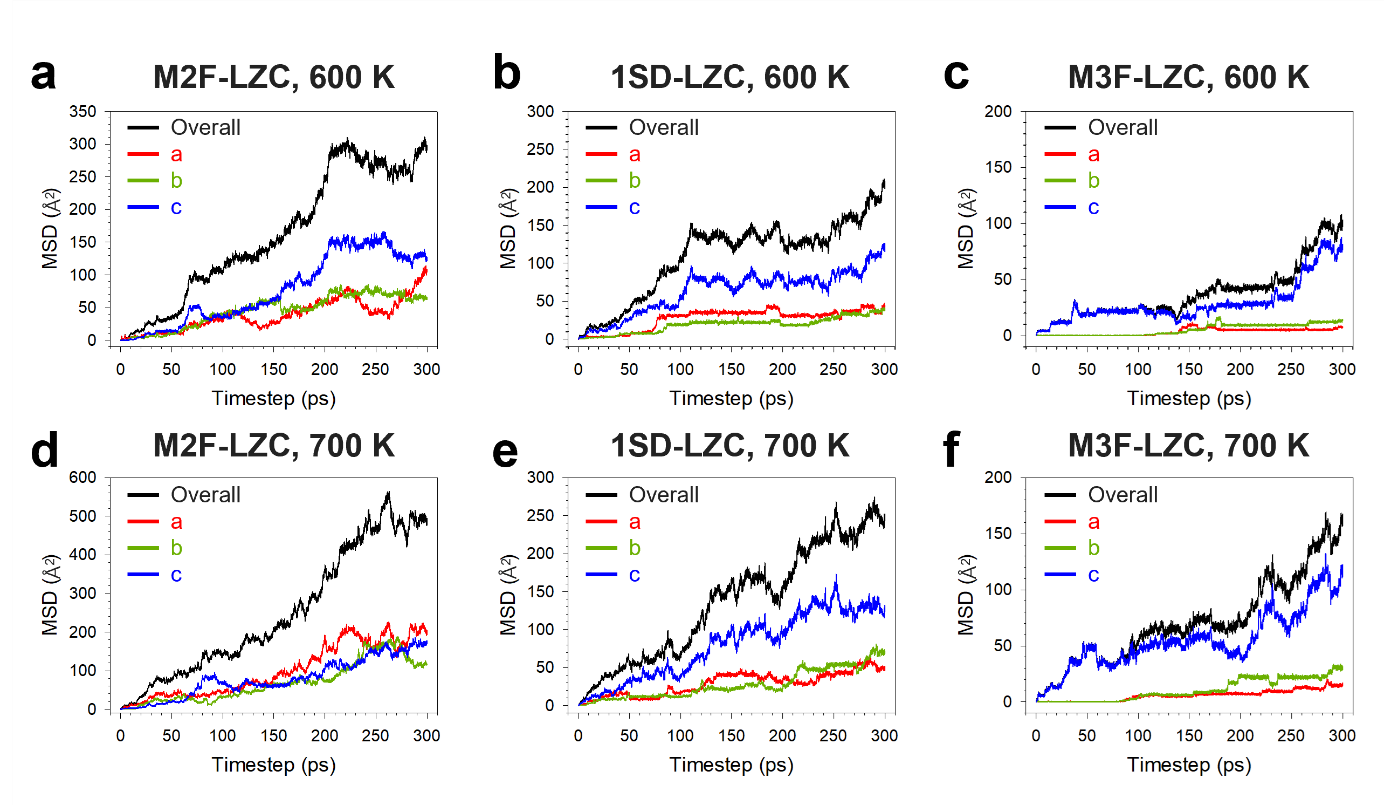
**

**Figure S19. a−f,** Mean square distance (MSD) obtained by AIMD simulation at 600 K (**a–c**) and 700 K (**d–f**) during 300 ps of M2F-Li_2_ZrCl_6_ (**a, d**), 1SD-Li_2_ZrCl_6_ (**b, e**), and M3F-Li_2_ZrCl_6_ (**c, f**).

**
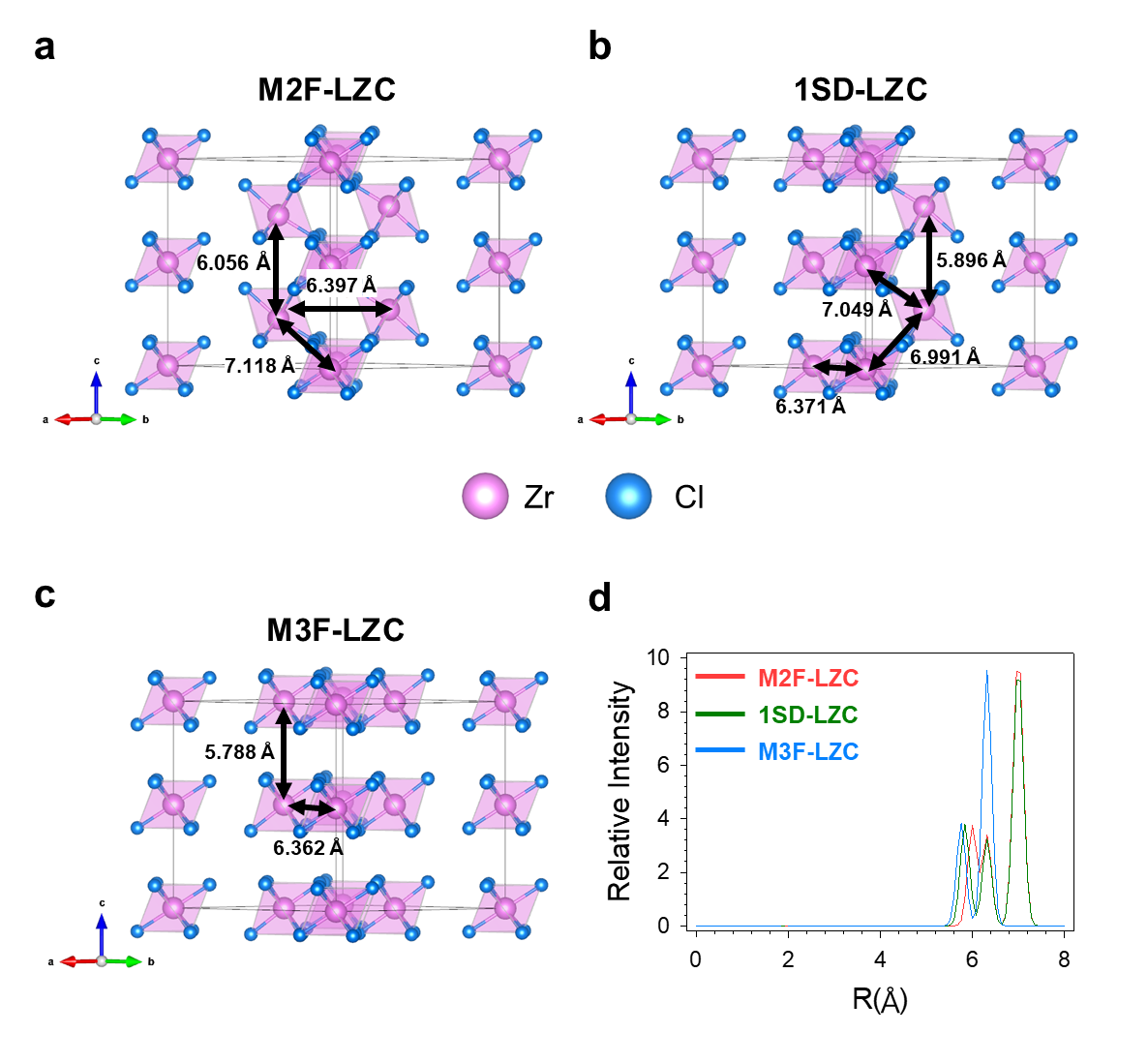
**

**Figure S20.** **a−d,** Crystal structures and atomic distances between Zr-Zr of M2F-Li_2_ZrCl_6_ (**a**), 1SD-Li_2_ZrCl_6_ (**b**), and M3F-Li_2_ZrCl_6_ (**c**), with simulated Zr-Zr RDF spectra (**d**). The arrows and numbers represent the distance between Zr atoms.

**
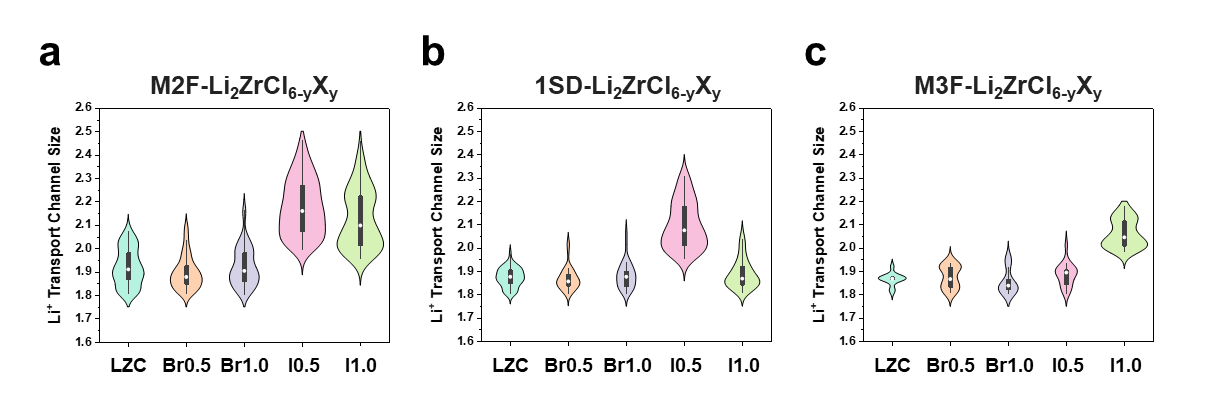
**

**Figure S21. a−c,** Topological analysis and Li^+^ transport channel size in M2F (**a**), 1SD (**b**), and M3F structures (**c**) of Li_2_ZrCl_6_, Li_2_ZrCl_5.5_Br_0.5,_ Li_2_ZrCl_5_Br, Li_2_ZrCl_5.5_I_0.5_, and Li_2_ZrCl_5_I, represented as LZC, Br0.5, Br1.0, I0.5, I1.0, respectively. The white circles, boxes, and whiskers indicate the median value, values from 25% to 75% percentiles, and values from 10% to 90% percentiles, respectively.

**
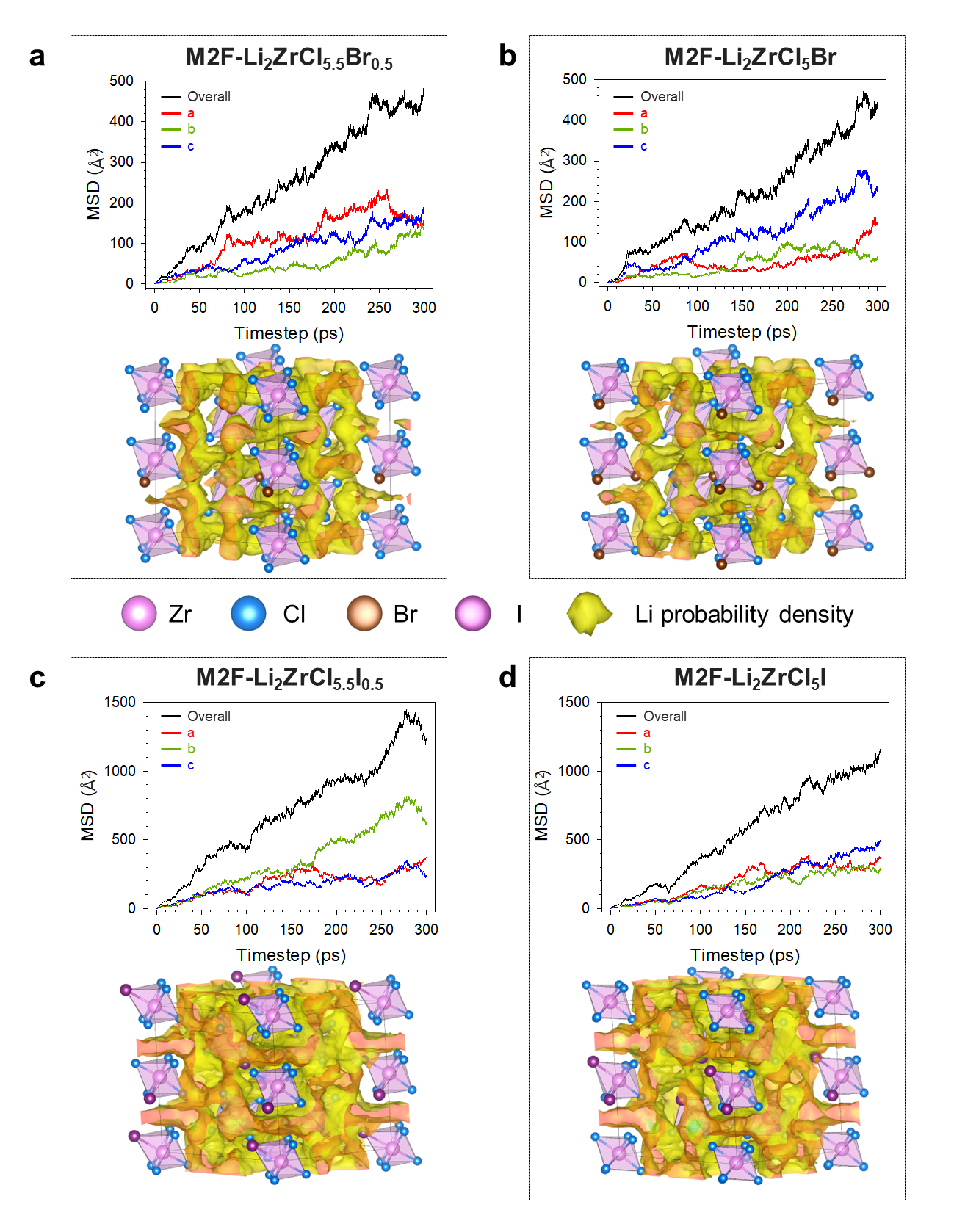
**

**Figure S22. a−d,** Mean square distance (MSD) and Li probability density at 600 K during 300 ps (isosurface value P = P_max_/100), as obtained by AIMD simulation of M2F-structured Li_2_ZrCl_5.5_Br_0.5_ (**a**), Li_2_ZrCl_5_Br (**b**), Li_2_ZrCl_5.5_I_0.5_ (**c**), and Li_2_ZrCl_5_I (**d**).

**
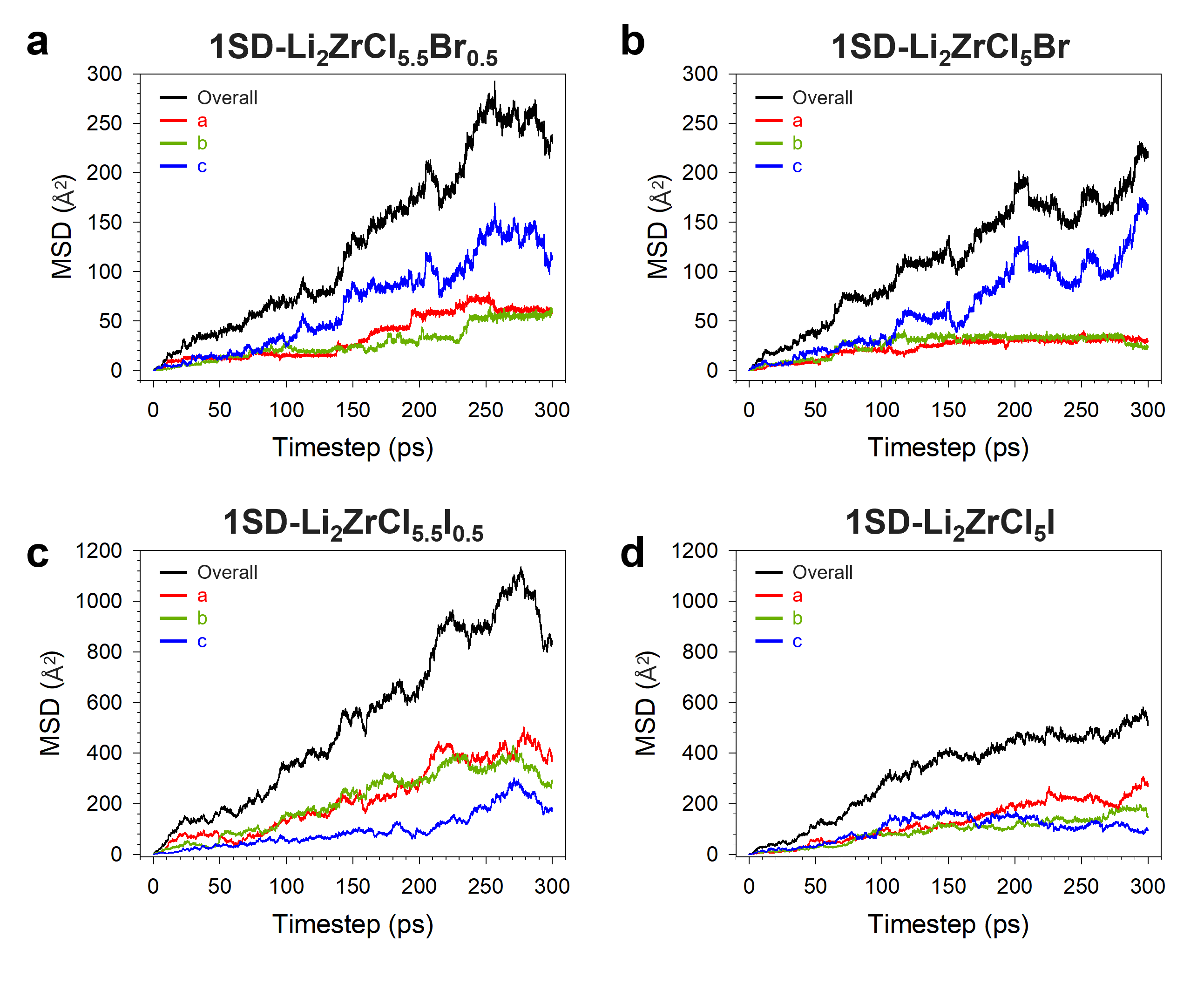
**

**Figure S23. a−d,** Mean square distance (MSD) at 600 K during 300 ps, as obtained by AIMD simulation of 1SD-structured Li_2_ZrCl_5.5_Br_0.5_ (**a**), Li_2_ZrCl_5_Br (**b**), Li_2_ZrCl_5.5_I_0.5_ (**c**), and Li_2_ZrCl_5_I (**d**).

**
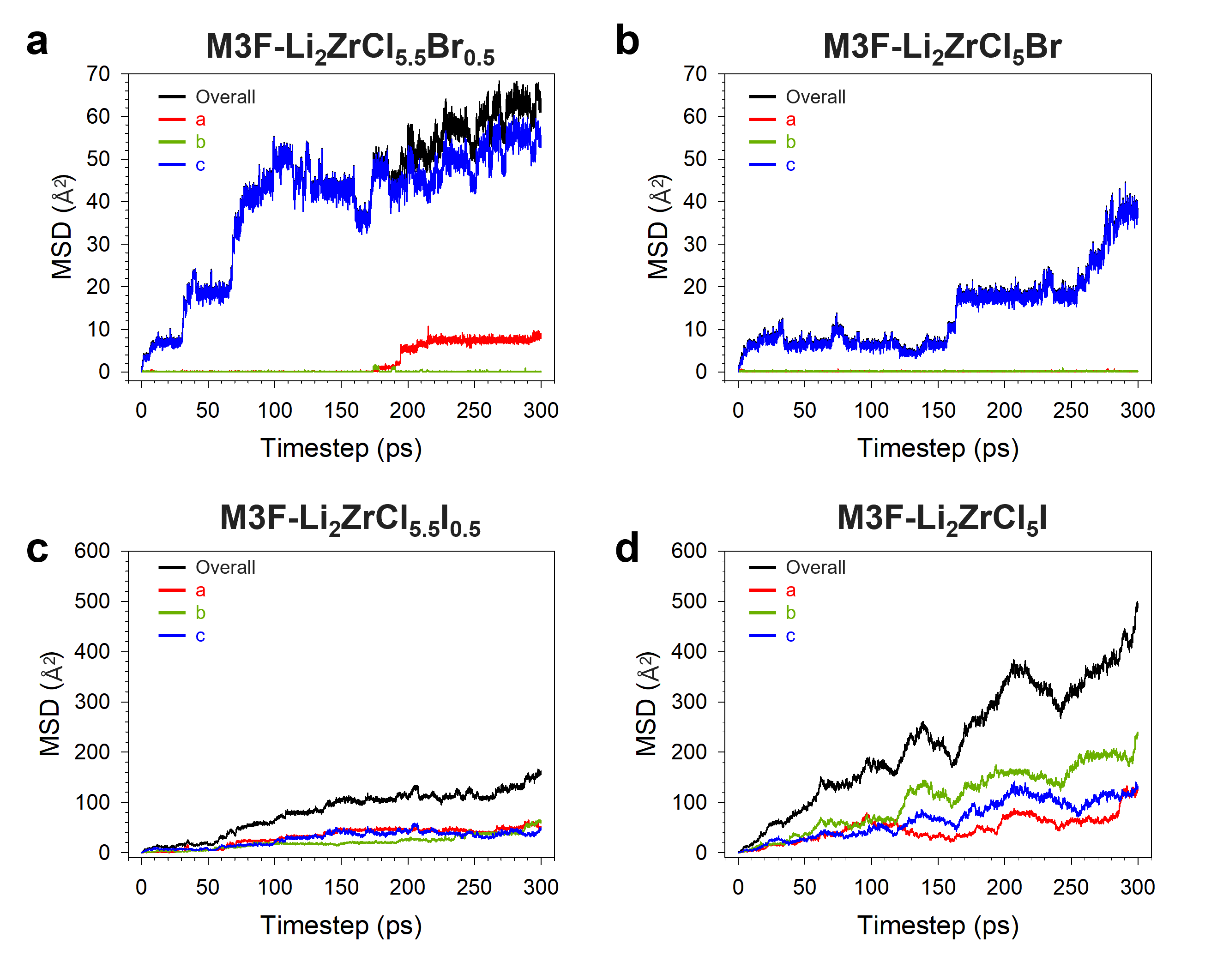
**

**Figure S24. a−d,** Mean square distance (MSD) at 600 K during 300 ps, as obtained by AIMD simulation of M3F-structured Li_2_ZrCl_5.5_Br_0.5_ (**a**), Li_2_ZrCl_5_Br (**b**), Li_2_ZrCl_5.5_I_0.5_ (**c**), and Li_2_ZrCl_5_I (**d**)

**
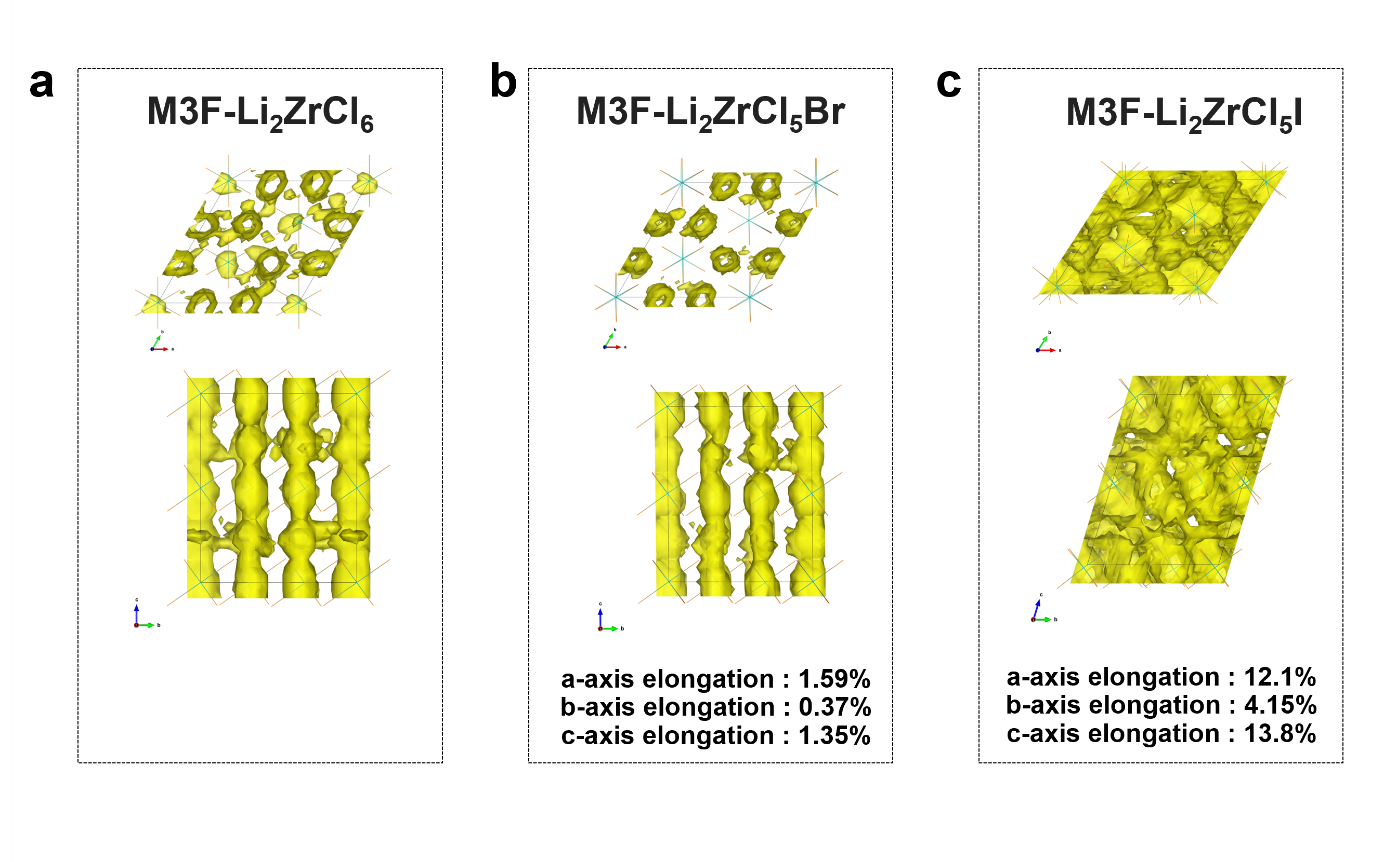
**

**Figure S25. a−c,** Li probability density at 600 K during 300 ps (isosurface value P = P_max_/100), as obtained by AIMD simulation of M3F–structured Li_2_ZrCl_6_ (**a**), Li_2_ZrCl_5_Br (**b**), and Li_2_ZrCl_5_I (**c**). Zr-Cl, Zr-Br, and Zr-I bonds are represented by thin lines, while yellow surfaces denote Li probability densities.

**
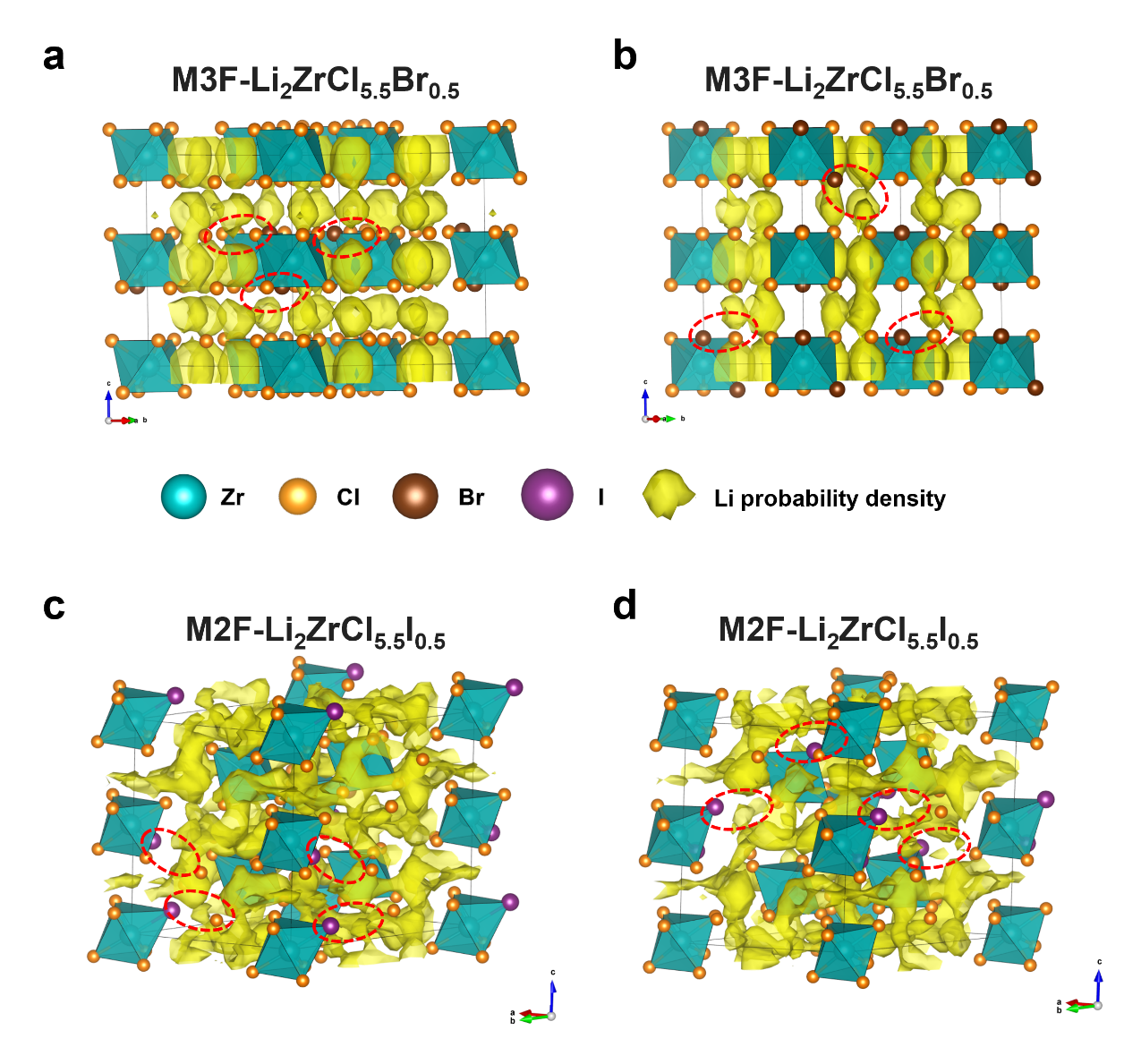
**

**Figure S26. a-d,** Li probability density of AIMD simulations at 600 K (~300 ps) of M3F-Li_2_ZrCl_5.5_Br_0.5_ (P = P_max_/200) (**a**), M3F-Li_2_ZrCl_5_Br (P=P_max_/200) (**b**), M2F-Li_2_ZrCl_5.5_I_0.5_ (P = P_max_/15) (**c**), and M2F-Li_2_ZrCl_5_I (P = P_max_/15) (**d**). The red circle shows disconnected Li density points near the Br/I anions and yellow surfaces denote Li probability densities.


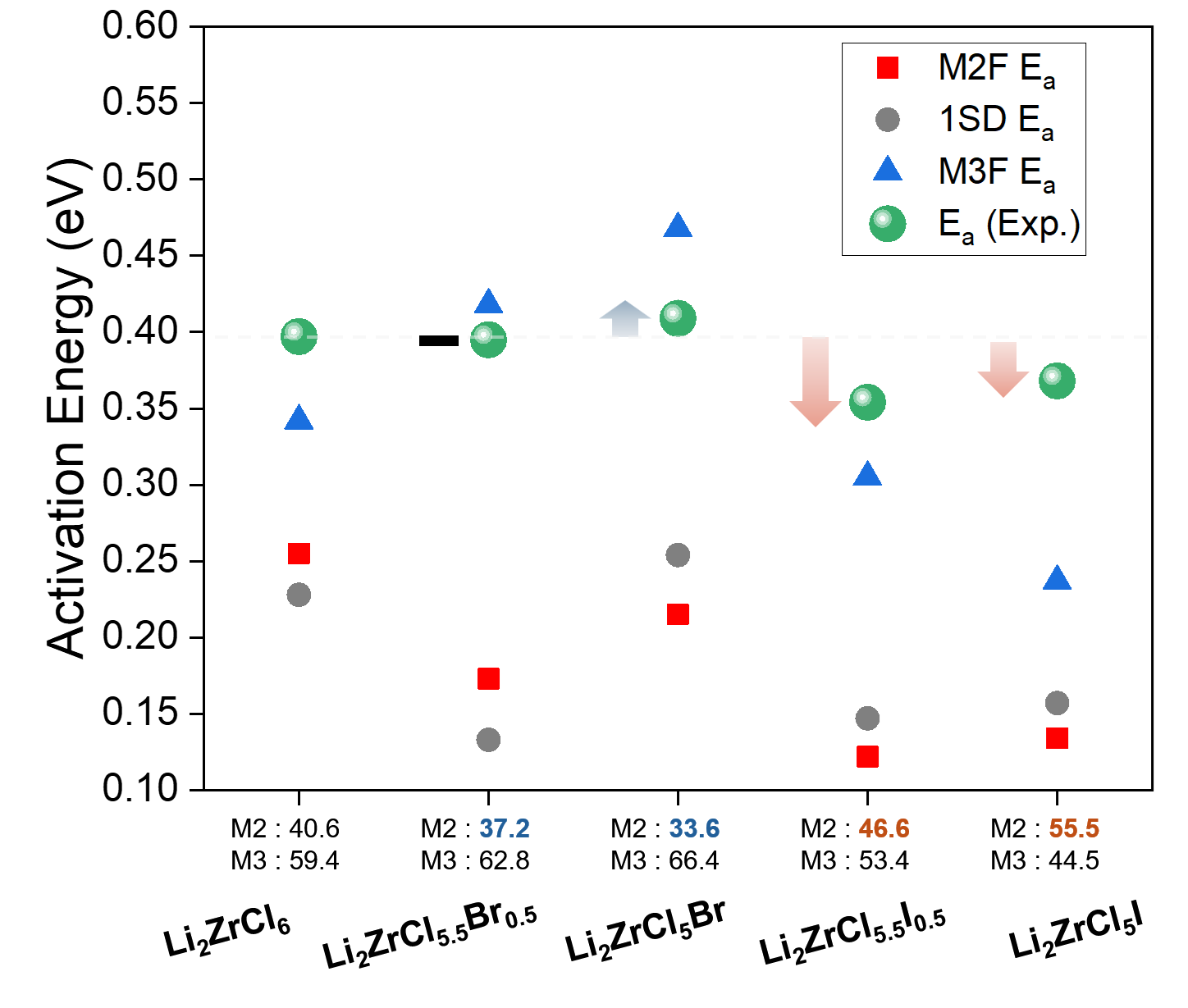


**Figure S27.** Activation energy and M2/M3 site occupancy of M2F, 1SD and M3F structure of LZCX and experimental activation energy of LZCX.


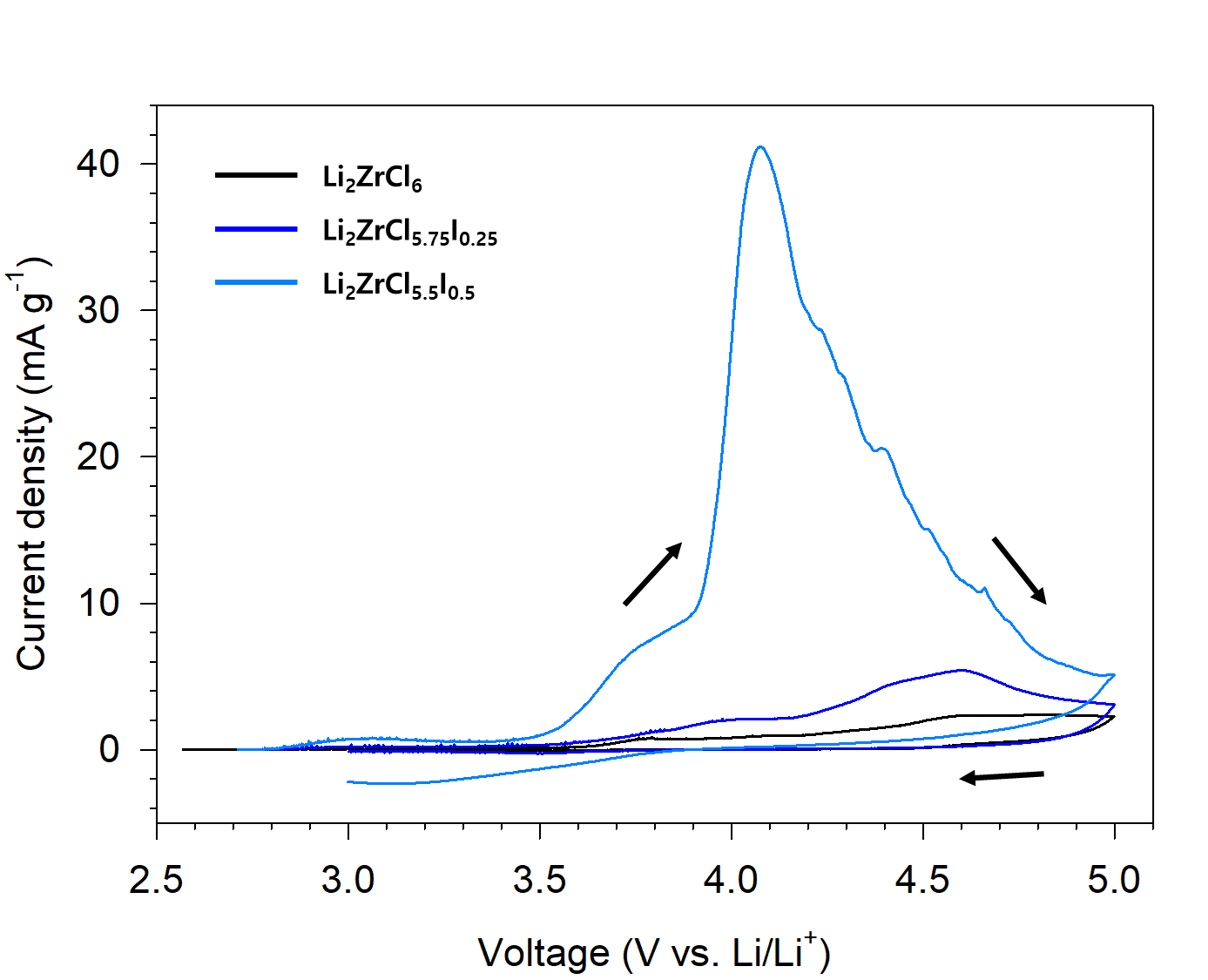


**Figure S28**. Cyclic voltammetry (CV) curves of Li_2_ZrCl_6_, Li_2_ZrCl_5.75_I_0.25_, and Li_2_ZrCl_5.5_I_0.5_ composite electrodes (SE : Super C65 = 10:1, w/w) measured at a scan rate of 0.1 mV s^-1^.


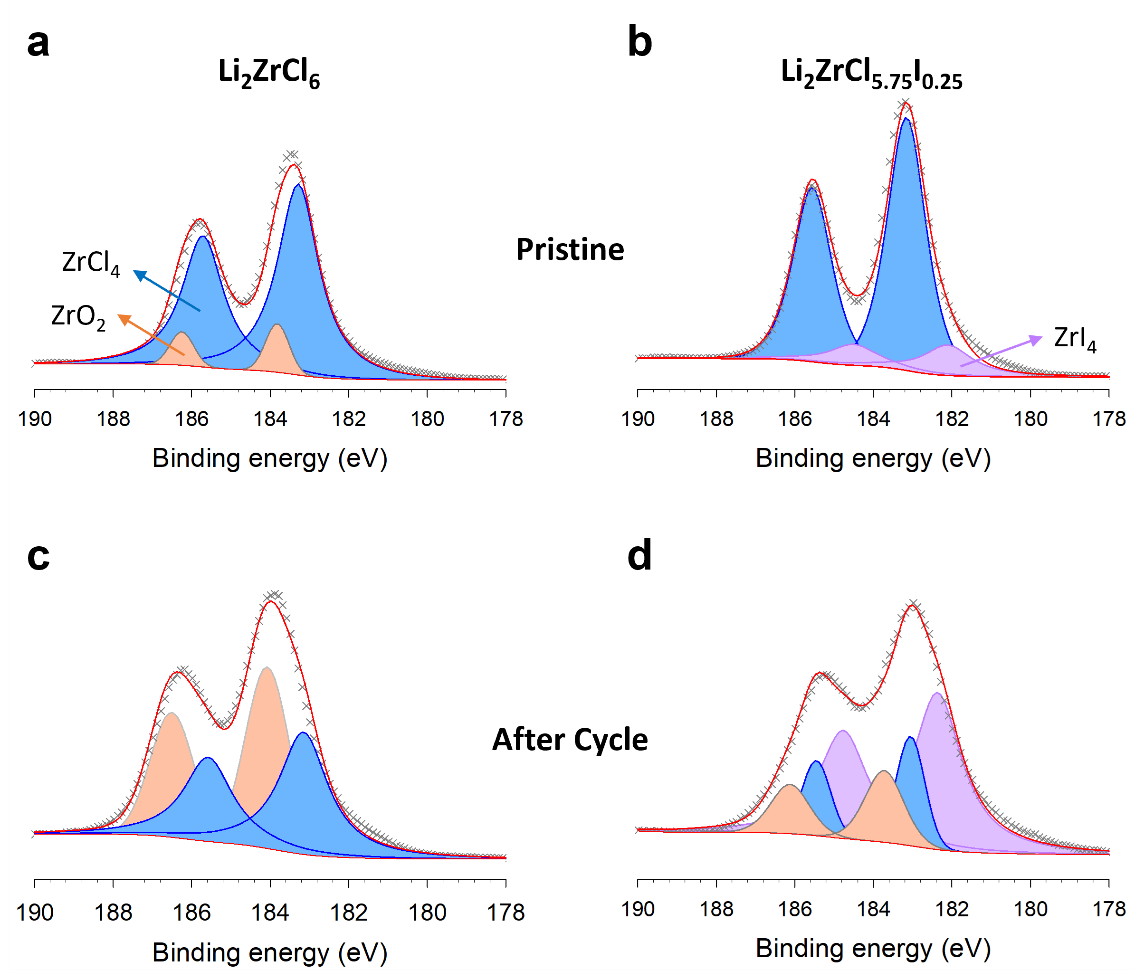


**Figure S29.** **a**−**d**, Ex situ Zr 3d XPS results of LCO electrodes with Li_2_ZrCl_6_ (**a**, **c**) or Li_2_ZrCl_5.75_I_0.25_ (**b**, **d**) before cycling (**a**, **b**) and after cycling (**c**, **d**). An increase in ZrO_2_ (for Li_2_ZrCl_6_) or both ZrO_2_ and ZrI_4_ (for Li_2_ZrCl_5.75_I_0.25_) was observed after cycling, indicating oxidative decomposition.

**
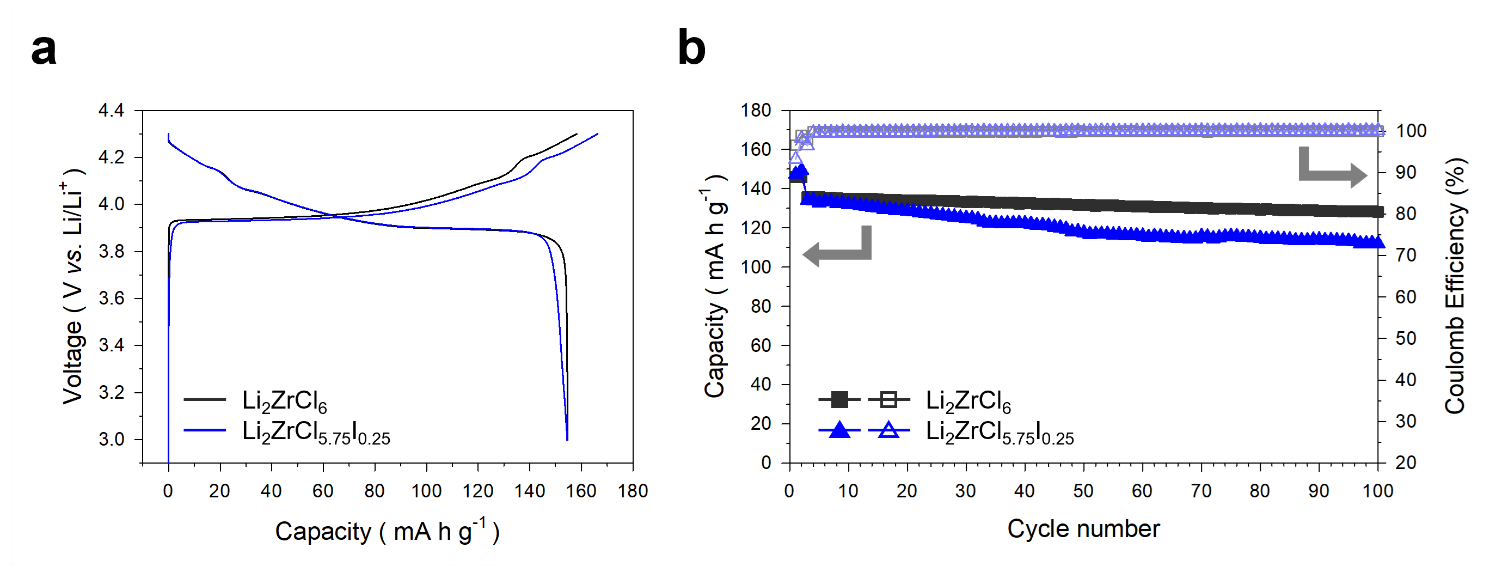
**

**Figure S30. Li–In||LiCoO2 ASSB cells at 30 °C.** **a**, First-cycle charge–discharge voltage profiles at 16.4 mA g^-1^ for LCO electrodes using LZC and Li_2_ZrCl_5.75_I_0.25_. **b**, Corresponding cycling performances with Coulombic efficiency at 82.0 mA g^-1^. The first two cycles were tested at 16.4 mA g^-1^. The specific current and capacity were determined based on the mass of active material (10.2 mg). All the cells were cycled under a pressure of 70 MPa.

**
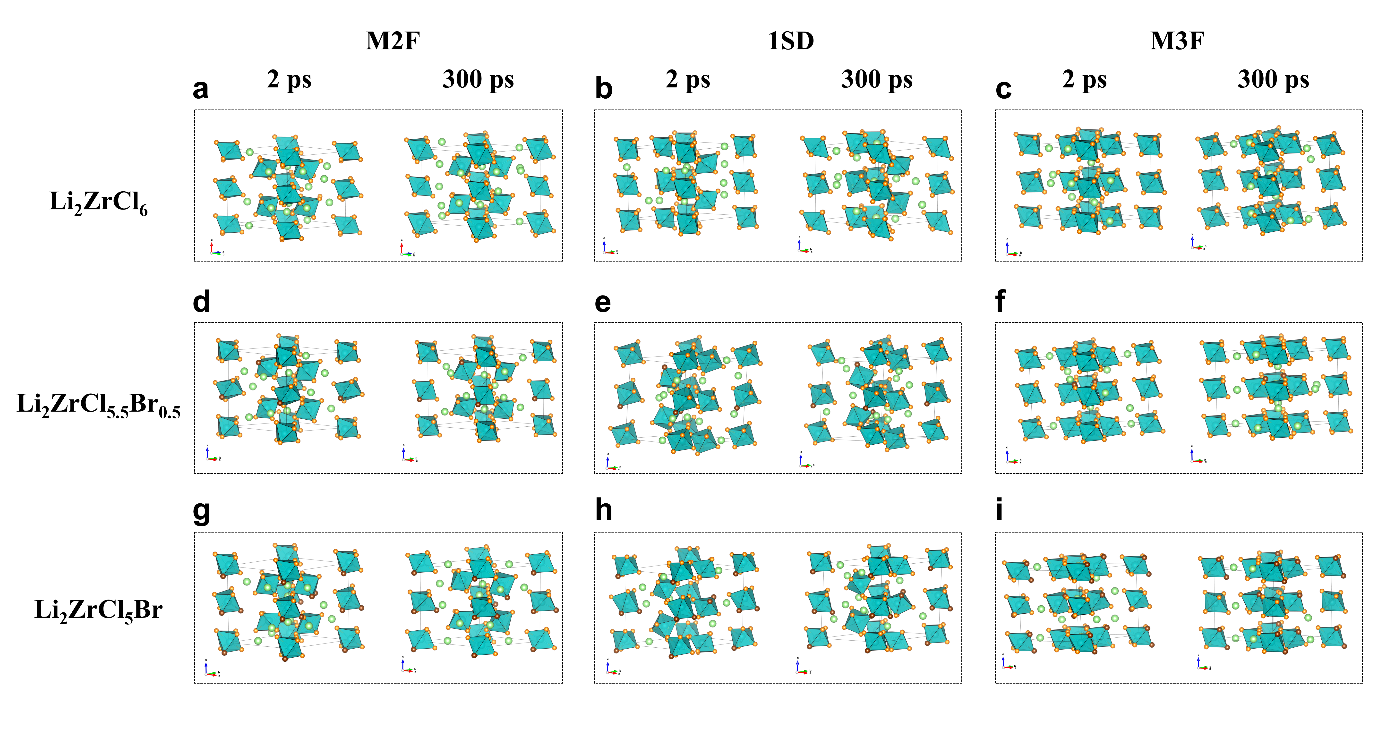
**

**Figure S31. a−i,** Initial and final configurations in AIMD simulations for Li^+^ diffusion of Li_2_ZrCl_6_ (**a−c**), Li_2_ZrCl_5.5_Br_0.5_ (**d−f**), and Li_2_ZrCl_5_Br (**g−i**).

**
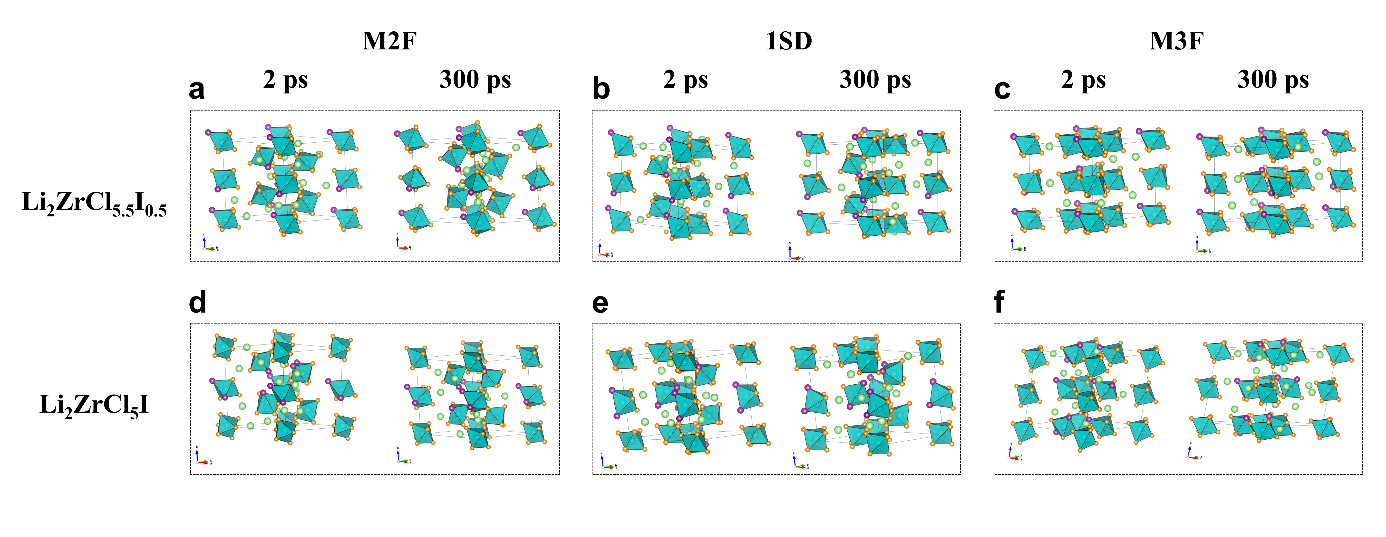
**

**Figure S32. a−f,** Initial and final configurations in AIMD simulations for Li^+^ diffusion of Li_2_ZrCl_5.5_I_0.5_ (**a−c**) and Li_2_ZrCl_5_I (**d−f**).

**
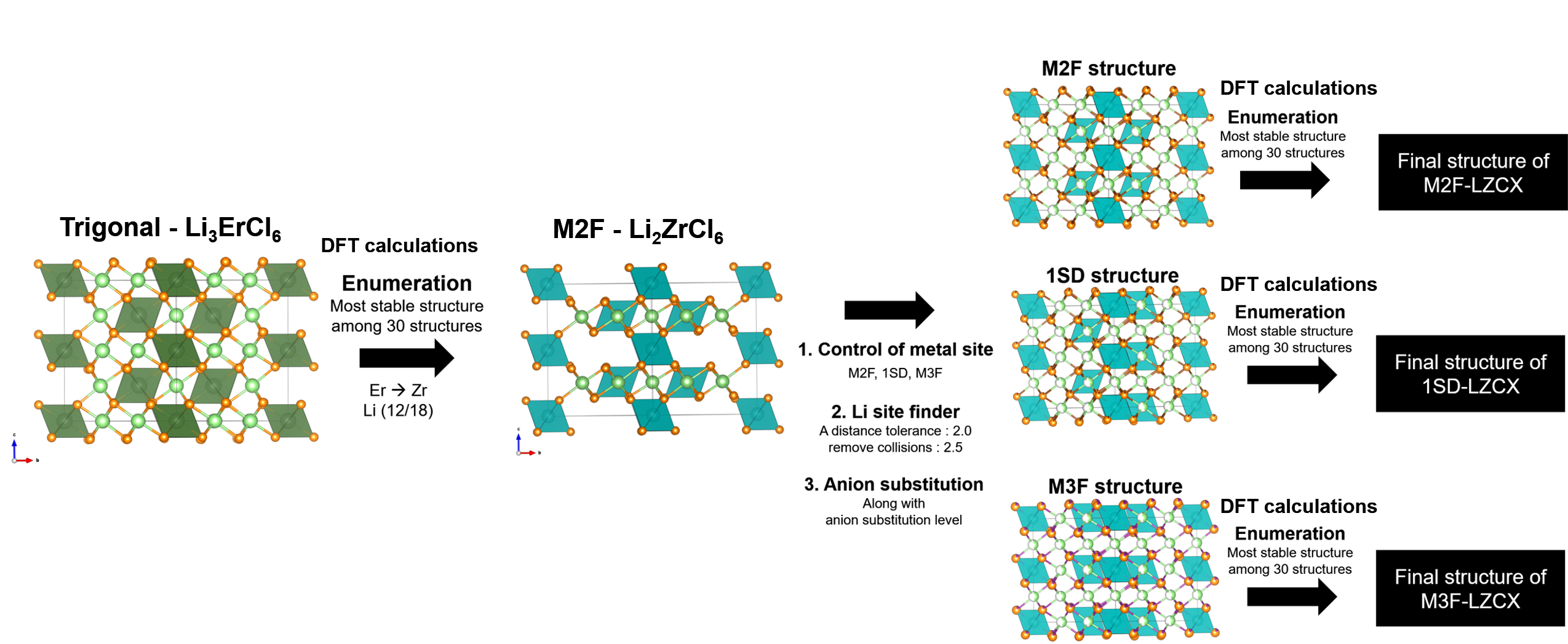
**

**Figure S33.** Schematic of the overall process for finding the most stable structures of M2F, 1SD and M3F LZCX structures.


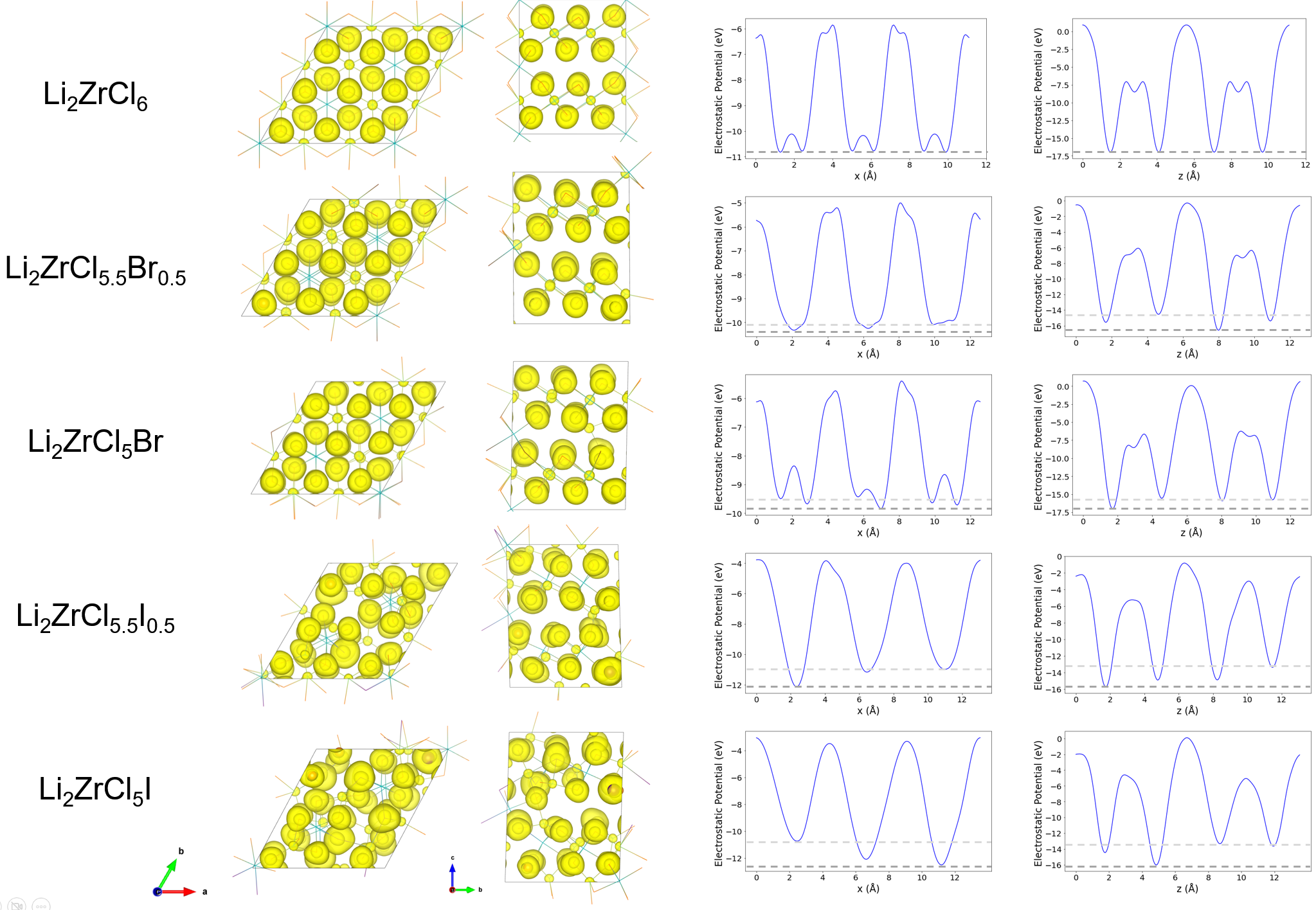


**Figure S34.** 3D ELF map (left) and electrostatic local potential analyses (right) for Li_2_ZrCl_6_ and its Br^-^ and I^-^substituted structures.


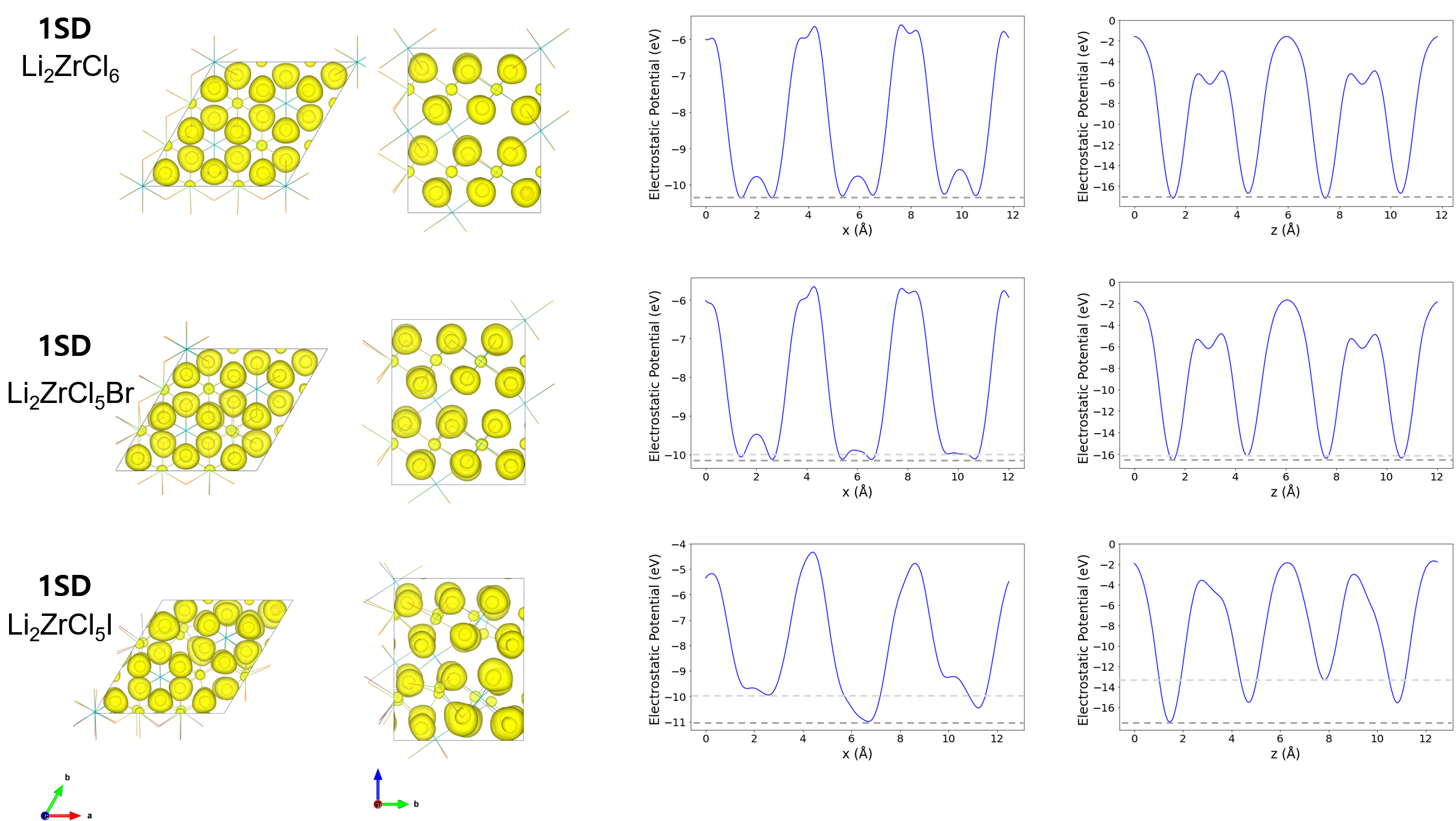


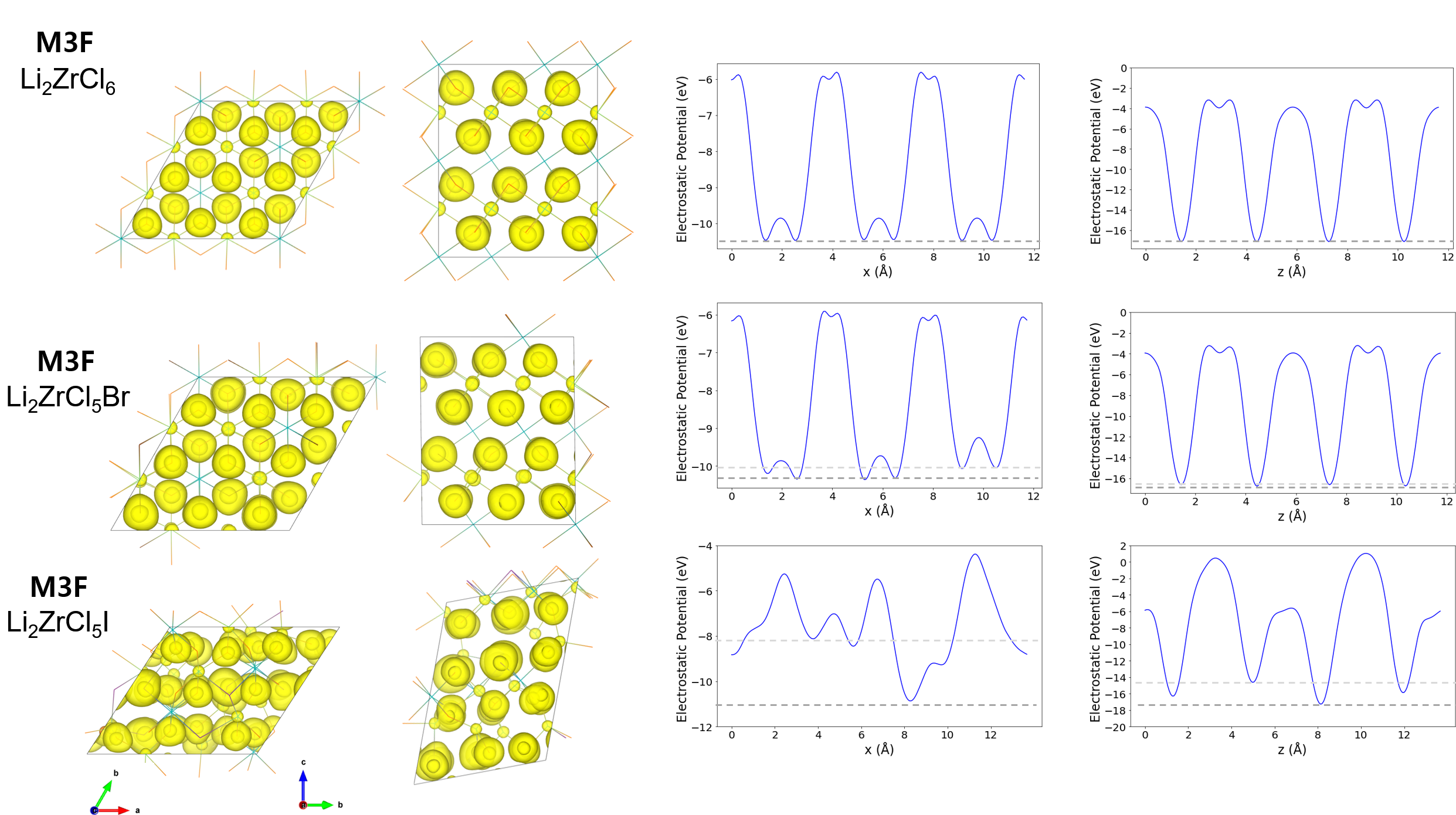


**Figure S35.** Electron localization and electrostatic potential analyses for Li_2_ZrCl_6_ and its Br^-^ and I^-^substituted disordered (1SD and M3F) structures.


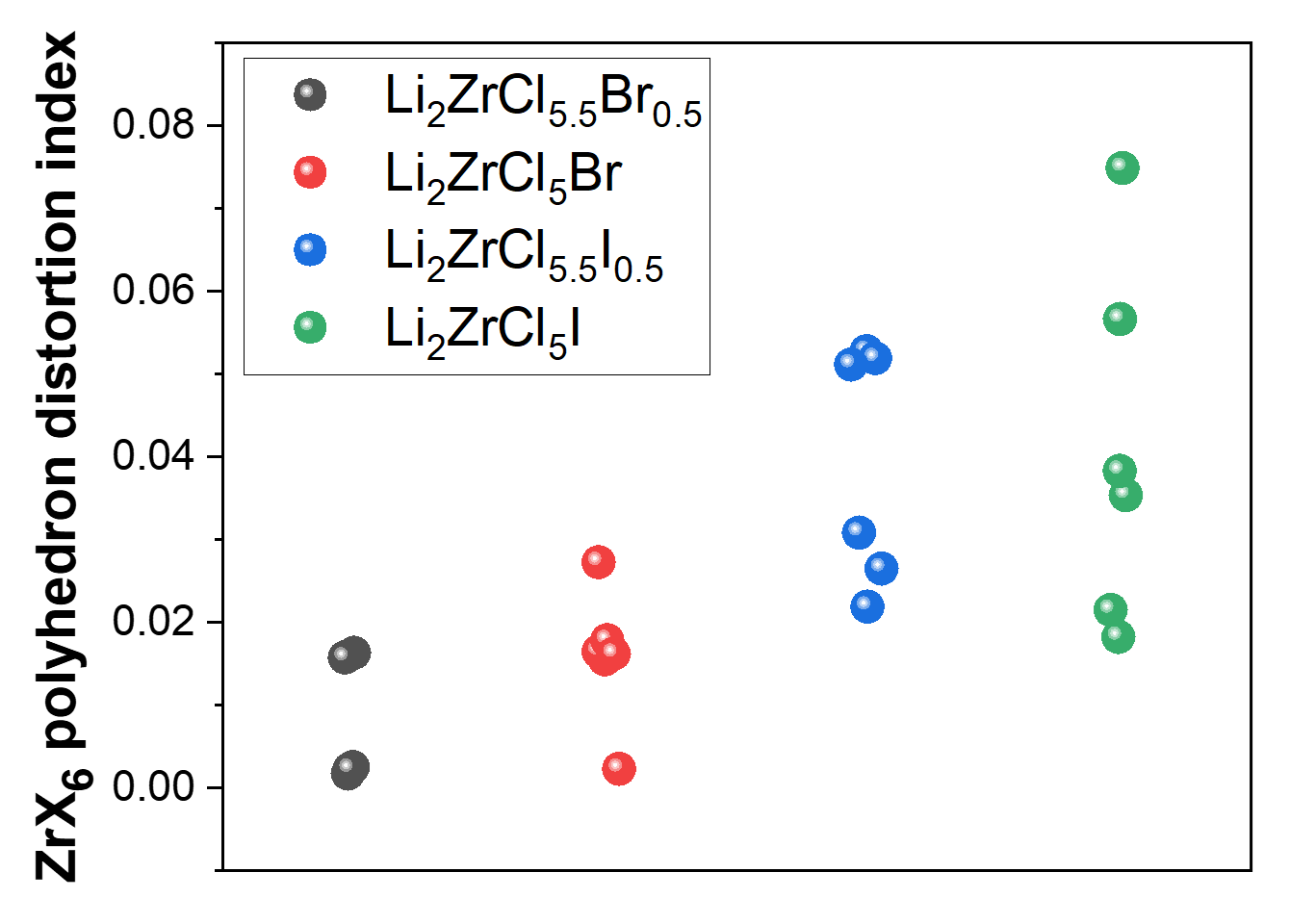


**Figure S36.** Distortion index in bond length of ZrX_6_ (X=Cl, Br, I) polyhedral unit in Li_2_ZrCl_5.5_Br_0.5_, Li_2_ZrCl_5.5_I_0.5_ Li_2_ZrCl_5_Br and Li_2_ZrCl_5_I ($Distortion index=\frac{1}{n}\sum_{i=1}^{n} \frac{\left| d_{i}-d_{avg} \right|}{d_{avg}}$). Distortion index of polyhedral in Li_2_ZrCl_6_ is all 0.


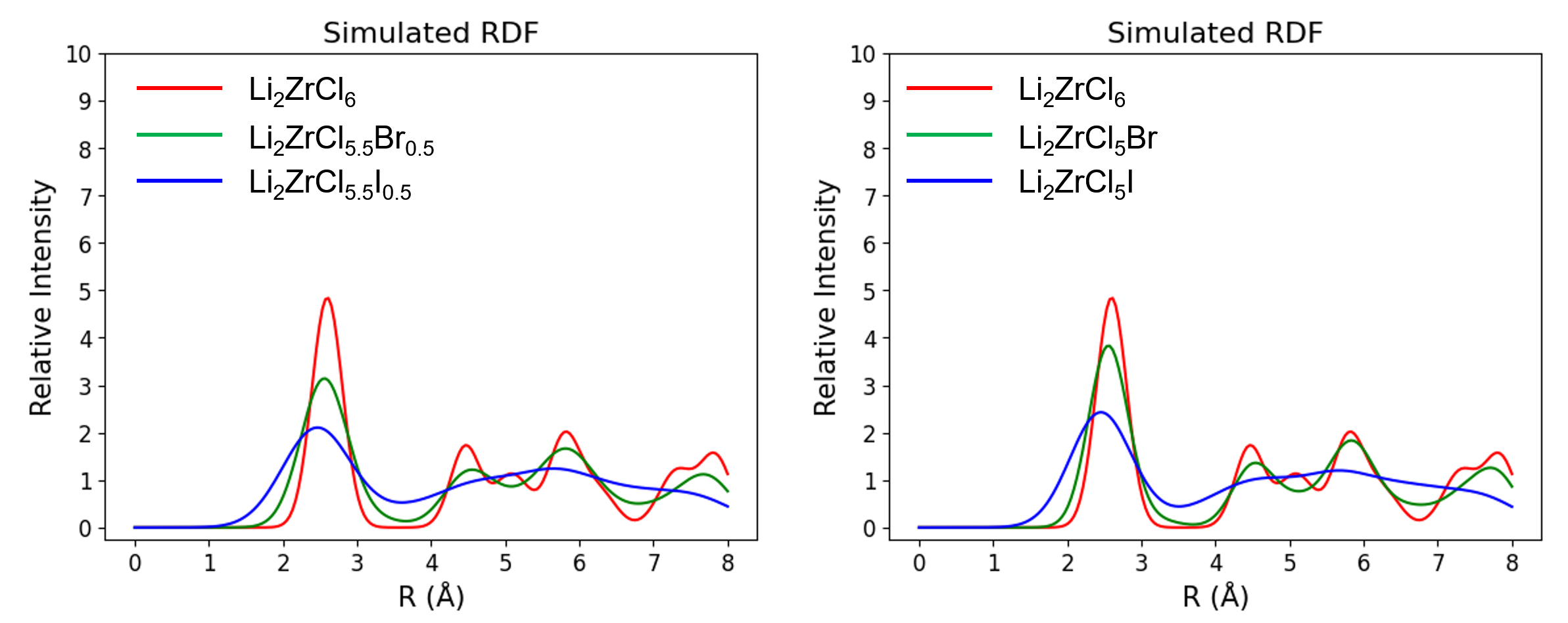


**Figure S37.** Simulated radial distribution (RDF) analysis of Li-Cl in Li_2_ZrCl_6_, Li_2_ZrCl_5.5_Br_0.5_, Li_2_ZrCl_5.5_I_0.5_ Li_2_ZrCl_5_Br and Li_2_ZrCl_5_I (sigma = 0.2).

**3. Supplementary Tables**

**Table S1.** Summary of Zr K-edge EXAFS spectra data.

| Li_2_ZrCl_6-y_X_y_ | | Bond | N^a^ | Range of error | r (Å)^b^ | Range of error  (Å) | Average r (Å)^b^ | D/W (Å^2^)^c^ | Range of error  (Å^2^) | R-factor (%)^d^ |
| --- | --- | --- | --- | --- | --- | --- | --- | --- | --- | --- |
| X | y |  |  |  |  |  |  |  |  |  |
| Li_2_ZrCl_6_ | | Zr-Cl | 6 | - | 2.478 | 0.0042 | - | 0.00473 | 0.0003 | 0.590 |
| Br | 0.50 | Zr-Cl | 5.64 | 0.1389 | 2.483 | 0.0066 | 2.49002 | 0.00598 | 0.0003 | 0.348 |
|  |  | Zr-Br | 0.36 |  | 2.600 | 0.0419 |  | 0.00598 | 0.0003 |  |
|  | 1.00 | Zr-Cl | 5.13 | 0.1475 | 2.484 | 0.0076 | 2.50271 | 0.00667 | 0.0003 | 0.422 |
|  |  | Zr-Br | 0.87 |  | 2.613 | 0.0174 |  | 0.00667 | 0.0003 |  |
|  | 1.50 | Zr-Cl | 4.60 | 0.1936 | 2.485 | 0.0110 | 2.51603 | 0.00709 | 0.0004 | 0.786 |
|  |  | Zr-Br | 1.40 |  | 2.618 | 0.0131 |  | 0.00709 | 0.0004 |  |
|  | 2.00 | Zr-Cl | 3.97 | 0.1357 | 2.497 | 0.0084 | 2.53828 | 0.00641 | 0.0003 | 0.472 |
|  |  | Zr-Br | 2.03 |  | 2.619 | 0.0057 |  | 0.00641 | 0.0003 |  |
| I | 0.25 | Zr-Cl | 5.73 | 0.4716 | 2.482 | 0.0043 | 2.49321 | 0.00593 | 0.0009 | 0.212 |
|  |  | Zr-I | 0.27 |  | 2.731 | 0.0864 |  | 0.01272 | 0.0235 |  |
|  | 0.50 | Zr-Cl | 5.70 | 0.3704 | 2.483 | 0.0041 | 2.49835 | 0.00622 | 0.0007 | 0.338 |
|  |  | Zr-I | 0.30 |  | 2.790 | 0.0722 |  | 0.01144 | 0.0161 |  |
|  | 1.00 | Zr-Cl | 5.37 | 0.2544 | 2.484 | 0.0046 | 2.51981 | 0.00590 | 0.0005 | 0.384 |
|  |  | Zr-I | 0.63 |  | 2.825 | 0.0198 |  | 0.00627 | 0.0032 |  |
|  | 1.50 | Zr-Cl | 4.69 | 0.3153 | 2.489 | 0.0051 | 2.56411 | 0.00657 | 0.0008 | 0.523 |
|  |  | Zr-I | 1.31 |  | 2.833 | 0.0173 |  | 0.01047 | 0.0029 |  |
|  | 2.00 | Zr-Cl | 4.30 | 0.2318 | 2.487 | 0.0046 | 2.58617 | 0.00601 | 0.0006 | 0.432 |
|  |  | Zr-I | 1.70 |  | 2.837 | 0.0081 |  | 0.00669 | 0.0011 |  |

^a^Coordination number; ^b^Bond length; ^c^Debye-Waller factor; ^d^EXAFS R-factor

***** E_0_ (eV) = 18008.45, S_0_^2^ = 0.93. All spectra were Fourier transformed in k-range of 3.2 to 12 Å^-1^ and fitted in R range of 1.25 to 3.5 Å

**Table S2.** PDF refinement results of LZCX in range of 1.5−20 Å. As Li_2_ZrCl_4_Br_2_ shows high R_w_ factor (low reliability), PDF refinement result of Li_2_ZrCl_4_Br_2_ in range of 1.5−10 Å is also included.

| Li_2_ZrCl_6-y_X_y_ | | Lattice parameter | | M2 site occupancy | M3 site occupancy | R_w_ (%) |
| --- | --- | --- | --- | --- | --- | --- |
| X | y | a = b (Å) | c (Å) |  |  |  |
| Li_2_ZrCl_6_ | | 10.97 | 5.94 | 40.6 | 59.4 | 12.5 |
| Br | 0.50 | 11.03 | 5.99 | 37.2 | 62.9 | 12.3 |
|  | 1.00 | 11.11 | 6.01 | 33.6 | 66.4 | 14.1 |
|  | 1.50 | 11.15 | 6.08 | 32.8 | 67.2 | 16.9 |
|  | 2.00 | 11.20 | 6.13 | 39.6 | 60.4 | 23.5 |
|  | 2.00  (1.5−10 Å) | 11.19 | 6.13 | 26.0 | 74.0 | 18.3 |
| I | 0.25 | 11.03 | 6.01 | 45.4 | 54.6 | 13.8 |
|  | 0.50 | 11.08 | 6.06 | 46.6 | 53.4 | 11.4 |
|  | 0.75 | 11.07 | 6.06 | 48.7 | 51.3 | 11.9 |
|  | 1.0 | 11.26 | 6.19 | 55.5 | 44.5 | 17.4 |
|  | 1.5 | 11.37 | 6.22 | 65.7 | 34.3 | 24.7 |
|  | 2.0 | 11.71 | 6.51 | 57.3 | 42.7 | 35.6 |

**Table S3.** Energy above hull (meV/atom) of M2F, 1SD, M3F configurations of Li_2_ZrCl_5.5_Br_0.5,_ Li_2_ZrCl_5_Br, Li_2_ZrCl_5.5_I_0.5_, and Li_2_ZrCl_5_I.

| Composition | E_hull_ (meV/atom) | | |
| --- | --- | --- | --- |
|  | M2F | 1SD | M3F |
| Li_2_ZrCl_6_ | 7 | 8 | 23 |
| Li_2_ZrCl_5.5_Br_0.5_ | 26 | 15 | 25 |
| Li_2_ZrCl_5_Br | 25 | 12 | 24 |
| Li_2_ZrCl_5.5_I_0.5_ | 12 | 22 | 43 |
| Li_2_ZrCl_5_I | 19 | 18 | 32 |

**Table S4** Lattice parameters and lattice volume of M2F-, 1SD-, and M3F-Li_2_ZrCl_6_, Li_2_ZrCl_5.5_Br_0.5,_ Li_2_ZrCl_5_Br, Li_2_ZrCl_5.5_I_0.5_, and Li_2_ZrCl_5_I. All structures are fully relaxed by the same parameters of DFT calculations.

| Structure | Sample | a (Å) | b (Å) | c (Å) | α (°) | β (°) | γ (°) | Lattice volume (Å^3^) |
| --- | --- | --- | --- | --- | --- | --- | --- | --- |
| M2F | Li_2_ZrCl_6_ | 11.07438 | 11.07438 | 12.11263 | 90 | 90 | 60 | 1286.49477 |
|  | Li_2_ZrCl_5.5_Br_0.5_ | 11.15692 | 11.10746 | 12.50937 | 90.3439 | 90.1299 | 59.6927 | 1338.333641 |
|  | Li_2_ZrCl_5_Br | 11.14501 | 11.19488 | 12.53555 | 89.2637 | 90.435 | 60.0543 | 1354.936047 |
|  | Li_2_ZrCl_5.5_I_0.5_ | 11.39283 | 11.79989 | 13.01265 | 92.7325 | 92.9096 | 59.567 | 1505.889563 |
|  | Li_2_ZrCl_5_I | 11.381 | 11.61662 | 13.48996 | 90.6683 | 94.3568 | 61.9478 | 1568.828652 |
| 1SD | Li_2_ZrCl_6_ | 11.03501 | 11.03501 | 11.73204 | 90 | 90 | 60 | 1243.55412 |
|  | Li_2_ZrCl_5.5_Br_0.5_ | 11.05453 | 11.0962 | 11.92741 | 90.0627 | 90.2852 | 60.005 | 1267.089574 |
|  | Li_2_ZrCl_5_Br | 11.13506 | 11.15707 | 11.98479 | 89.9289 | 89.9353 | 59.8705 | 1287.761586 |
|  | Li_2_ZrCl_5.5_I_0.5_ | 11.45276 | 11.55193 | 13.10119 | 89.2471 | 89.5363 | 60.0997 | 1492.221837 |
|  | Li_2_ZrCl_5_I | 11.44864 | 11.64463 | 12.44715 | 90.538 | 91.3028 | 58.7035 | 1417.564449 |
| M3F | Li_2_ZrCl_6_ | 11.01928 | 11.01928 | 11.57555 | 90 | 90 | 60 | 1217.24706 |
|  | Li_2_ZrCl_5.5_Br_0.5_ | 11.10005 | 11.0459 | 11.63641 | 90.1722 | 89.8614 | 60.0087 | 1235.685379 |
|  | Li_2_ZrCl_5_Br | 11.19466 | 11.06009 | 11.70159 | 90.204 | 89.3848 | 60.2178 | 1257.321065 |
|  | Li_2_ZrCl_5.5_I_0.5_ | 11.40573 | 11.26624 | 11.87629 | 87.8639 | 89.1007 | 59.1131 | 1308.751313 |
|  | Li_2_ZrCl_5_I | 12.34755 | 11.47679 | 13.1673 | 75.3624 | 89.1007 | 59.1131 | 1512.63714 |

**Table S5.** HRPD Rietveld refinement results of Li_2_ZrCl_6_ in 10 wt% LiF–90 wt% Li_2_ZrCl_6_.

| **Crystal system** | | | Trigonal | | |
| --- | --- | --- | --- | --- | --- |
| **Space group** | | | *P*$\bar{3}$*m*1 (164) | | |
| **Lattice parameter** | | | a = b = 10.96773(20), c = 5.93249(19) | | |
| **Atom** | **x** | **y** | **z** | **B_iso_ (Å^2^)** | **Occ.** |
| Li1 | 0.33920 | 0 | 0 | 4 | 0.75 |
| Li2 | 0.32520 | 0 | 0.5 | 4 | 0.25 |
| Zr1 | 0 | 0 | 0 | 2.488(21) | 1 |
| Zr2 | 0.33333 | 0.66667 | 0.48318(524) | 2.488(21) | 0.452(5) |
| Zr3 | 0.33333 | 0.66667 | -0.00127(464) | 2.488(21) | 0.548(5) |
| Cl1 | 0.10512(80) | -0.10512(80) | 0.76674(140) | 2.488(21) | 0.75 |
| Cl2 | 0.22305(124) | -0.22305(124) | 0.26658(117) | 2.488(21) | 0.75 |
| Cl3 | 0.43658(97) | -0.43658(97) | 0.74709(124) | 2.488(21) | 0.75 |
| R_p_ = 4.53 %, R_wp_ = 5.87 %, R_exp_ = 3.99, R_bragg_ = 4.99 %, R_f_ = 4.60 % | | | | | |

**Table S6.** HRPD Rietveld refinement results of Li_2_ZrCl_5.5_Br_0.5_ in 10 wt% LiF–90 wt% Li_2_ZrCl_5.5_Br_0.5_.

| **Crystal system** | | | Trigonal | | |
| --- | --- | --- | --- | --- | --- |
| **Space group** | | | *P*$\bar{3}$*m*1 (164) | | |
| **Lattice parameter** | | | a = b = 11.03337(32), c = 5.98142(31) | | |
| **Atom** | **x** | **y** | **z** | **B_iso_ (Å^2^)** | **Occ.** |
| Li1 | 0.33920 | 0 | 0 | 4 | 0.75 |
| Li2 | 0.32520 | 0 | 0.5 | 4 | 0.25 |
| Zr1 | 0 | 0 | 0 | 2.573(30) | 1 |
| Zr2 | 0.33333 | 0.66667 | 0.50721(719) | 2.573(30) | 0.407(7) |
| Zr3 | 0.33333 | 0.66667 | -0.01352(772) | 2.573(30) | 0.593(7) |
| Cl1 | 0.10854(134) | -0.10854(134) | 0.74516(135) | 2.573(30) | 0.08333 |
| Cl2 | 0.22642(168) | -0.22642(168) | 0.2608(143) | 2.573(30) | 0.08333 |
| Cl3 | 0.44269(83) | -0.44269(83) | 0.75300(172) | 2.573(30) | 0.08333 |
| Br1 | 0.10854(134) | -0.10854(134) | 0.74516(135) | 2.573(30) | 0.91667 |
| Br2 | 0.22642(168) | -0.22642(168) | 0.2608(143) | 2.573(30) | 0.91667 |
| Br3 | 0.44269(83) | -0.44269(83) | 0.75300(172) | 2.573(30) | 0.91667 |
| R_p_ = 6.55 %, R_wp_ = 8.29 %, R_exp_ = 4.28, R_bragg_ = 10.6 %, R_f_ = 8.95 % | | | | | |

**Table S7.** HRPD Rietveld refinement results of Li_2_ZrCl_5_Br in 10 wt% LiF–90 wt% Li_2_ZrCl_5_Br.

| **Crystal system** | | | Trigonal | | |
| --- | --- | --- | --- | --- | --- |
| **Space group** | | | *P*$\bar{3}$*m*1 (164) | | |
| **Lattice parameter** | | | a = b = 11.08944(56), c = 6.02082(33) | | |
| **Atom** | **x** | **y** | **z** | **B_iso_ (Å^2^)** | **Occ.** |
| Li1 | 0.33920 | 0 | 0 | 4 | 0.75 |
| Li2 | 0.32520 | 0 | 0.5 | 4 | 0.25 |
| Zr1 | 0 | 0 | 0 | 3.234(43) | 1 |
| Zr2 | 0.33333 | 0.66667 | 0.47368(632) | 3.234(43) | 0.377(9) |
| Zr3 | 0.33333 | 0.66667 | -0.01693(375) | 3.234(43) | 0.623(9) |
| Cl1 | 0.10951(89) | -0.10951(89) | 0.75303(177) | 3.234(43) | 0.83333 |
| Cl2 | 0.22432(114) | -0.22432(114) | 0.28496(177) | 3.234(43) | 0.83333 |
| Cl3 | 0.43985(137) | -0.43985(137) | 0.76225(183) | 3.234(43) | 0.83333 |
| Br1 | 0.10951(89) | -0.10951(89) | 0.75303(177) | 3.234(43) | 0.16667 |
| Br2 | 0.22432(114) | -0.22432(114) | 0.28496(177) | 3.234(43) | 0.16667 |
| Br3 | 0.43985(137) | -0.43985(137) | 0.76225(183) | 3.234(43) | 0.16667 |
| R_p_ = 5.57 %, R_wp_ = 7.03 %, R_exp_ = 3.86, R_bragg_ = 13.8 %, R_f_ = 11.1 % | | | | | |

**Table S8.** HRPD Rietveld refinement results of Li_2_ZrCl_4.5_Br_1.5_ in 10 wt% LiF–90 wt% Li_2_ZrCl_4.5_Br_1.5_.

| **Crystal system** | | | Trigonal | | |
| --- | --- | --- | --- | --- | --- |
| **Space group** | | | *P*$\bar{3}$*m*1 (164) | | |
| **Lattice parameter** | | | a = b = 11.13998(26), c = 6.05696(28) | | |
| **Atom** | **x** | **y** | **z** | **B_iso_ (Å^2^)** | **Occ.** |
| Li1 | 0.33920 | 0 | 0 | 4 | 0.75 |
| Li2 | 0.32520 | 0 | 0.5 | 4 | 0.25 |
| Zr1 | 0 | 0 | 0 | 3.413(39) | 1 |
| Zr2 | 0.33333 | 0.66667 | 0.47342(582) | 3.413(39) | 0.371(10) |
| Zr3 | 0.33333 | 0.66667 | -0.02577(513) | 3.413(39) | 0.629(10) |
| Cl1 | 0.10385(121) | -0.10385(121) | 0.74685(184) | 3.413(39) | 0.75 |
| Cl2 | 0.22496(110) | -0.22496(110) | 0.27916(128) | 3.413(39) | 0.75 |
| Cl3 | 0.44922(164) | -0.44922(164) | 0.75246(163) | 3.413(39) | 0.75 |
| Br1 | 0.10385(121) | -0.10385(121) | 0.74685(184) | 3.413(39) | 0.25 |
| Br2 | 0.22496(110) | -0.22496(110) | 0.27916(128) | 3.413(39) | 0.25 |
| Br3 | 0.44922(164) | -0.44922(164) | 0.75246(163) | 3.413(39) | 0.25 |
| R_p_ = 4.81 %, R_wp_ = 6.24 %, R_exp_ = 3.75, R_bragg_ = 8.53 %, R_f_ = 8.57 % | | | | | |

**Table S9.** HRPD Rietveld refinement results of Li_2_ZrCl_4_Br_2_ in 10 wt% LiF–90 wt% Li_2_ZrCl_4_Br_2_.

| **Crystal system** | | | Trigonal | | |
| --- | --- | --- | --- | --- | --- |
| **Space group** | | | *P*$\bar{3}$*m*1 (164) | | |
| **Lattice parameter** | | | a = b = 11.18985(42), c = 6.08999(29) | | |
| **Atom** | **x** | **y** | **z** | **B_iso_ (Å^2^)** | **Occ.** |
| Li1 | 0.33920 | 0 | 0 | 4 | 0.75 |
| Li2 | 0.32520 | 0 | 0.5 | 4 | 0.25 |
| Zr1 | 0 | 0 | 0 | 3.294(46) | 1 |
| Zr2 | 0.33333 | 0.66667 | 0.47117(442) | 3.294(46) | 0.306(11) |
| Zr3 | 0.33333 | 0.66667 | -0.01898(314) | 3.294(46) | 0.694(11) |
| Cl1 | 0.11038(57) | -0.11038(57) | 0.74856(182) | 3.294(46) | 0.66667 |
| Cl2 | 0.22664(79) | -0.22664(79) | 0.28844(142) | 3.294(46) | 0.66667 |
| Cl3 | 0.44445(59) | -0.44445(59) | 0.76427(180) | 3.294(46) | 0.66667 |
| Br1 | 0.11038(57) | -0.11038(57) | 0.74856(182) | 3.294(46) | 0.33333 |
| Br2 | 0.22664(79) | -0.22664(79) | 0.28844(142) | 3.294(46) | 0.33333 |
| Br3 | 0.44445(59) | -0.44445(59) | 0.76427(180) | 3.294(46) | 0.33333 |
| R_p_ = 4.94 %, R_wp_ = 6.72 %, R_exp_ = 3.79, R_bragg_ = 12.8 %, R_f_ = 17.4 % | | | | | |

**Table S10.** HRPD Rietveld refinement results of Li_2_ZrCl_5.75_I_0.25_ in 10 wt% LiF–90 wt% Li_2_ZrCl_5.75_I_0.25_.

| **Crystal system** | | | Trigonal | | |
| --- | --- | --- | --- | --- | --- |
| **Space group** | | | *P*$\bar{3}$*m*1 (164) | | |
| **Lattice parameter** | | | a = b = 11.02180(45), c = 5.99658(26) | | |
| **Atom** | **x** | **y** | **z** | **B_iso_ (Å^2^)** | **Occ.** |
| Li1 | 0.33920 | 0 | 0 | 4 | 0.75 |
| Li2 | 0.32520 | 0 | 0.5 | 4 | 0.25 |
| Zr1 | 0 | 0 | 0 | 3.026(26) | 1 |
| Zr2 | 0.33333 | 0.66667 | 0.50721(719) | 3.026(26) | 0.486(7) |
| Zr3 | 0.33333 | 0.66667 | -0.01352((772) | 3.026(26) | 0.514(7) |
| Cl1 | 0.10330(90) | -0.10330(90) | 0.77680(166) | 3.026(26) | 0.95833 |
| Cl2 | 0.23178(124) | -0.23178(124) | 0.2550(215) | 3.026(26) | 0.95833 |
| Cl3 | 0.44559(164) | -0.44559(164) | 0.74375(189) | 3.026(26) | 0.95833 |
| I1 | 0.10330(90) | -0.10330(90) | 0.77680(166) | 3.026(26) | 0.04167 |
| I2 | 0.23178(124) | -0.23178(124) | 0.2550(215) | 3.026(26) | 0.04167 |
| I3 | 0.44559(164) | -0.44559(164) | 0.74375(189) | 3.026(26) | 0.04167 |
| R_p_ = 4.39 %, R_wp_ = 5.63 %, R_exp_ = 3.77, R_bragg_ = 5.73 %, R_f_ = 6.02 % | | | | | |

**Table S11.** HRPD Rietveld refinement results of Li_2_ZrCl_5.5_I_0.5_ in 10 wt% LiF–90 wt% Li_2_ZrCl_5.5_I_0.5_.

| **Crystal system** | | | Trigonal | | |
| --- | --- | --- | --- | --- | --- |
| **Space group** | | | *P*$\bar{3}$*m*1 (164) | | |
| **Lattice parameter** | | | a = b = 11.07926(39), c = 6.05093(38) | | |
| **Atom** | **x** | **y** | **z** | **B_iso_ (Å^2^)** | **Occ.** |
| Li1 | 0.33920 | 0 | 0 | 4 | 0.75 |
| Li2 | 0.32520 | 0 | 0.5 | 4 | 0.25 |
| Zr1 | 0 | 0 | 0 | 3.699(49) | 1 |
| Zr2 | 0.33333 | 0.66667 | 0.46710(278) | 3.699(49) | 0.532(12) |
| Zr3 | 0.33333 | 0.66667 | 0.00776(358) | 3.699(49) | 0.468(12) |
| Cl1 | 0.11209(123) | -0.11209(123) | 0.76327(218) | 3.699(49) | 0.91667 |
| Cl2 | 0.23591(114) | -0.23591(114) | 0.27953(164) | 3.699(49) | 0.91667 |
| Cl3 | 0.44510(86) | -0.44510(86) | 0.74902(235) | 3.699(49) | 0.91667 |
| I1 | 0.11209(123) | -0.11209(123) | 0.76327(218) | 3.699(49) | 0.08333 |
| I2 | 0.23591(114) | -0.23591(114) | 0.27953(164) | 3.699(49) | 0.08333 |
| I3 | 0.44510(86) | -0.44510(86) | 0.74902(235) | 3.699(49) | 0.08333 |
| R_p_ = 4.29 %, R_wp_ = 5.43 %, R_exp_ = 3.46, R_bragg_ = 14.9 %, R_f_ = 19.6 % | | | | | |

**Table S12.** HRPD Rietveld refinement results of Li_2_ZrCl_5.25_I_0.75_ in 10 wt% LiF–90 wt% Li_2_ZrCl_5.25_I_0.75_.

| **Crystal system** | | | Trigonal | | |
| --- | --- | --- | --- | --- | --- |
| **Space group** | | | *P*$\bar{3}$*m*1 (164) | | |
| **Lattice parameter** | | | a = b = 11.13497(40), c = 6.10541(39) | | |
| **Atom** | **x** | **y** | **z** | **B_iso_ (Å^2^)** | **Occ.** |
| Li1 | 0.33920 | 0 | 0 | 4 | 0.75 |
| Li2 | 0.32520 | 0 | 0.5 | 4 | 0.25 |
| Zr1 | 0 | 0 | 0 | 4.034(55) | 1 |
| Zr2 | 0.33333 | 0.66667 | 0.47459(467) | 4.034(55) | 0.568(15) |
| Zr3 | 0.33333 | 0.66667 | 0.00096(841) | 4.034(55) | 0.432(15) |
| Cl1 | 0.10467(197) | -0.10467(197) | 0.72844(192) | 4.034(55) | 0.875 |
| Cl2 | 0.21315(376) | -0.21315(376) | 0.23337(224) | 4.034(55) | 0.875 |
| Cl3 | 0.43734(388) | -0.43734(388) | 0.72373(215) | 4.034(55) | 0.875 |
| I1 | 0.10467(197) | -0.10467(197) | 0.72844(192) | 4.034(55) | 0.125 |
| I2 | 0.21315(376) | -0.21315(376) | 0.23337(224) | 4.034(55) | 0.125 |
| I3 | 0.43734(388) | -0.43734(388) | 0.72373(215) | 4.034(55) | 0.125 |
| R_p_ = 3.77 %, R_wp_ = 4.78 %, R_exp_ = 3.37, R_bragg_ = 11.5 %, R_f_ = 13.0 % | | | | | |

**Table S13.** HRPD Rietveld refinement results of Li_2_ZrCl_5_I in 10 wt% LiF–90 wt% Li_2_ZrCl_5_I.

| **Crystal system** | | | Trigonal | | |
| --- | --- | --- | --- | --- | --- |
| **Space group** | | | *P*$\bar{3}$*m*1 (164) | | |
| **Lattice parameter** | | | a = b = 11.15340(44), c = 6.13715(63) | | |
| **Atom** | **x** | **y** | **z** | **B_iso_ (Å^2^)** | **Occ.** |
| Li1 | 0.33920 | 0 | 0 | 4 | 0.75 |
| Li2 | 0.32520 | 0 | 0.5 | 4 | 0.25 |
| Zr1 | 0 | 0 | 0 | 4.080(81) | 1 |
| Zr2 | 0.33333 | 0.66667 | 0.51809(920) | 4.080(81) | 0.639(20) |
| Zr3 | 0.33333 | 0.66667 | 0.92416(1797) | 4.080(81) | 0.361(20) |
| Cl1 | 0.10489(152) | -0.10489(152) | 0.75954(229) | 4.080(81) | 0.83333 |
| Cl2 | 0.22137(180) | -0.22137(180) | 0.29119(253) | 4.080(81) | 0.83333 |
| Cl3 | 0.43986(187) | -0.43986(187) | 0.71342(250) | 4.080(81) | 0.83333 |
| I1 | 0.10489(152) | -0.10489(152) | 0.75954(229) | 4.080(81) | 0.91667 |
| I2 | 0.22137(180) | -0.22137(180) | 0.29119(253) | 4.080(81) | 0.91667 |
| I3 | 0.43986(187) | -0.43986(187) | 0.71342(250) | 4.080(81) | 0.91667 |
| R_p_ = 3.43 %, R_wp_ = 4.34 %, R_exp_ = 3.36, R_bragg_ = 20.5 %, R_f_ = 28.7 % | | | | | |

**Table S14.** HRPD Rietveld refinement results of Li_2_ZrCl_4.5_I_1.5_ in 10 wt% LiF–90 wt% Li_2_ZrCl_4.5_I_1.5_. Atomic positions and value of atomic displacement parameters are less convincing due to insufficient intensity caused by the highly distorted structure of Li_2_ZrCl_4.5_I_1.5_.

| **Crystal system** | | | Trigonal | | |
| --- | --- | --- | --- | --- | --- |
| **Space group** | | | *P*$\bar{3}$*m*1 (164) | | |
| **Lattice parameter** | | | a = b = 11.34419(114), c = 6.24785(143) | | |
| **Atom** | **x** | **y** | **z** | **B_iso_ (Å^2^)** | **Occ.** |
| Li1 | 0.33920 | 0 | 0 | 4 | 0.75 |
| Li2 | 0.32520 | 0 | 0.5 | 4 | 0.25 |
| Zr1 | 0 | 0 | 0 | 2.513(106) | 1 |
| Zr2 | 0.33333 | 0.66667 | 0.48911(675) | 2.513(106) | 0.790(52) |
| Zr3 | 0.33333 | 0.66667 | 0.12263(2409) | 2.513(106) | 0.210(52) |
| Cl1 | 0.10331(164) | -0.10331(164) | 0.80890(596) | 2.513(106) | 0.75 |
| Cl2 | 0.23093(160) | -0.23093(160) | 0.23378(503) | 2.513(106) | 0.75 |
| Cl3 | 0.45940(124) | -0.45940(124) | 0.70470(593) | 2.513(106) | 0.75 |
| I1 | 0.10331(164) | -0.10331(164) | 0.80890(596) | 2.513(106) | 0.25 |
| I2 | 0.23093(160) | -0.23093(160) | 0.23378(503) | 2.513(106) | 0.25 |
| I3 | 0.45940(124) | -0.45940(124) | 0.70470(593) | 2.513(106) | 0.25 |
| R_p_ = 4.29 %, R_wp_ = 5.43 %, R_exp_ = 3.46, R_bragg_ = 14.9 %, R_f_ = 19.6 % | | | | | |

**Table S15.** HRPD profile matching results of Li_2_ZrCl_4_I_2._ Only profile matching results can be achieved due to the highly distorted structure of Li_2_ZrCl_4_I_2_.

| **Crystal system** | Trigonal |
| --- | --- |
| **Space group** | *P*$\bar{3}$*m*1 (164) |
| **Lattice parameter** | a = b = 11.46371(404), c = 6.30879(298) |
| R_p_ = 3.03 %, R_wp_ = 3.88 %, R_exp_ = 3.79, R_bragg_ = 8.52 %, R_f_ = 7.33 % | |

**Table S16.** Comparison of Zr M2 Site Occupancy and Lattice Parameters from HRPD and PDF. The Rietveld refinement of Li_2_ZrCl_4_I_2_ was unsuccessful due to its highly distorted structure.

| **Li_2_ZrCl_6-y_X_y_** | | **HRPD** | | | | **PDF** | | | |
| --- | --- | --- | --- | --- | --- | --- | --- | --- | --- |
| **x** | **y** | **M2 Occ. (%)** | **M3 Occ. (%)** | **a = b (Å)** | **c (Å)** | **M2 Occ. (%)** | **M3 Occ. (%)** | **a = b (Å)** | **c (Å)** |
| Li_2_ZrCl_6_ | | 45.2 | 54.8 | 10.97 | 5.93 | 40.6 | 59.4 | 10.97 | 5.94 |
| Br | 0.50 | 40.7 | 59.3 | 11.03 | 5.98 | 37.2 | 62.8 | 11.03 | 5.99 |
|  | 1.00 | 37.7 | 62.3 | 11.09 | 6.02 | 33.6 | 66.4 | 11.11 | 6.01 |
|  | 1.50 | 37.1 | 62.9 | 11.14 | 6.06 | 32.8 | 67.2 | 11.15 | 6.08 |
|  | 2.00 | 30.6 | 69.4 | 11.19 | 6.09 | 26.0 | 74.0 | 11.19 | 6.13 |
| I | 0.25 | 48.6 | 51.4 | 11.02 | 6.00 | 45.4 | 54.6 | 11.03 | 6.01 |
|  | 0.50 | 53.2 | 46.8 | 11.08 | 6.05 | 46.6 | 53.4 | 11.08 | 6.06 |
|  | 0.75 | 56.8 | 43.2 | 11.13 | 6.11 | 48.7 | 51.3 | 11.07 | 6.06 |
|  | 1.00 | 63.9 | 36.1 | 11.15 | 6.14 | 55.5 | 44.5 | 11.26 | 6.19 |
|  | 1.50 | 79.0 | 21.0 | 11.34 | 6.25 | 65.7 | 34.3 | 11.37 | 6.22 |
|  | 2.0 |  |  | 11.46 | 6.31 | 71.9 | 28.1 | 11.72 | 6.43 |

**Table S17.** Comparison of PDF refinement results of LZCI in range of 1.5−10 Å and 10−20 Å.

| Li_2_ZrCl_6-x_I_x_ | | Lattice parameter | | M2 site occupancy | M3 site occupancy | R_w_ (%) |
| --- | --- | --- | --- | --- | --- | --- |
| fitting  range | X | a=b (Å) | c (Å) |  |  |  |
| 1.5−10 Å | 1.00 | 11.36 | 6.12 | 54.9 | 45.1 | 12.8 |
|  | 1.50 | 11.55 | 6.16 | 69.7 | 30.3 | 11.8 |
|  | 2.00 | 11.72 | 6.43 | 71.9 | 28.1 | 17.0 |
| 10−20 Å | 1.00 | 11.29 | 6.20 | 55.04 | 45.0 | 6.3 |
|  | 1.50 | 11.40 | 6.29 | 63.8 | 36.2 | 7.7 |
|  | 2.00 | 11.59 | 6.48 | 73.7 | 26.3 | 9.4 |

**Table S18.** Lattice parameters and lattice volume of M2F-Li_2_ZrCl_6_, 1SD-Li_2_ZrCl_6,_ and M3F-Li_2_ZrCl_6_, including the decrease rate of lattice parameter c and lattice volume compared to that of M2F-Li_2_ZrCl_6_

| Structure | a (Å) | b (Å) | c (Å) | α (°) | β (°) | γ (°) | Lattice volume (Å^3^) | Decrease rate of c  (vs. M2F) (%) | Decrease rate of lattice volume (vs. M2F) (%) |
| --- | --- | --- | --- | --- | --- | --- | --- | --- | --- |
| M2F-Li_2_ZrCl_6_ | 11.07438 | 11.07438 | 12.11263 | 90 | 90 | 60 | 1286.49477 | - | - |
| 1SD-Li_2_ZrCl_6_ | 11.03501 | 11.03501 | 11.73204 | 90 | 90 | 60 | 1243.55412 | 3.14 | 3.30 |
| M3F-Li_2_ZrCl_6_ | 11.01928 | 11.01928 | 11.57555 | 90 | 90 | 60 | 1217.24706 | 4.43 | 5.38 |

**Table S19.** Lattice parameters and lattice volumes of M2F-Li_3_YCl_6_, 1SD-Li_3_YCl_6,_ and M3F-Li_3_YCl_6_, including a decrease in lattice parameter c and lattice volume compared to that of M2F-Li_3_YCl_6_

| Structure | a (Å) | b (Å) | c (Å) | α (°) | β (°) | γ (°) | Lattice volume (Å^3^) | Decrease rate of c  (vs. M2F) (%) | Decrease rate of lattice volume (vs. M3F) (%) |
| --- | --- | --- | --- | --- | --- | --- | --- | --- | --- |
| M2F-Li_3_YCl_6_ | 11.17641 | 11.17641 | 12.40780 | 89.9151 | 89.9151 | 119.1166 | 1354.02535 | - | - |
| 1SD- Li_3_YCl_6_ | 11.25163 | 11.10078 | 12.38452 | 89.9586 | 89.6971 | 119.5443 | 1345.697303 | 0.18 | 0.61 |
| M3F- Li_3_YCl_6_ | 11.19177 | 11.19177 | 12.19491 | 90.0731 | 90.0731 | 119.9412 | 1323.62108 | 1.72 | 2.25 |

**Table S20.** Li^+^ diffusivity at 600 K during 300 ps along a, b, and c axes, as calculated by AIMD simulations.

| Structure | Composition | Diffusivity along axis, 600 K (cm^2^ s^-1^) | | |
| --- | --- | --- | --- | --- |
|  |  | a | b | c |
| M2F | Li_2_ZrCl_6_ | 1.20 × 10^-5^ | 1.31 × 10^-5^ | 2.87 × 10^-5^ |
|  | Li_2_ZrCl_5.5_Br_0.5_ | 3.24 × 10^-5^ | 1.87 × 10^-5^ | 2.77 × 10^-5^ |
|  | Li_2_ZrCl_5_Br | 1.28 × 10^-5^ | 1.54 × 10^-5^ | 4.12 × 10^-5^ |
|  | Li_2_ZrCl_5.5_I_0.5_ | 4.49 × 10^-5^ | 1.31 × 10^-4^ | 3.95 × 10^-5^ |
|  | Li_2_ZrCl_5_I | 6.18 × 10^-5^ | 5.33 × 10^-5^ | 8.06 × 10^-5^ |
| 1SD | Li_2_ZrCl_6_ | 6.11 × 10^-6^ | 5.26 × 10^-6^ | 1.49 × 10^-5^ |
|  | Li_2_ZrCl_5.5_Br_0.5_ | 1.23 × 10^-5^ | 9.20 × 10^-6^ | 2.60 × 10^-5^ |
|  | Li_2_ZrCl_5_Br | 5.06 × 10^-6^ | 4.45 × 10^-6^ | 2.33 × 10^-5^ |
|  | Li_2_ZrCl_5.5_I_0.5_ | 7.78 × 10^-5^ | 6.75 × 10^-5^ | 3.75 × 10^-5^ |
|  | Li_2_ZrCl_5_I | 4.47 × 10^-5^ | 2.80 × 10^-5^ | 1.75 × 10^-5^ |
| M3F | Li_2_ZrCl_6_ | 1.32 × 10^-6^ | 2.57 × 10^-6^ | 8.73 × 10^-6^ |
|  | Li_2_ZrCl_5.5_Br_0.5_ | 1.68 × 10^-6^ | 6.48 × 10^-9^ | 7.33 × 10^-6^ |
|  | Li_2_ZrCl_5_Br | 1.12 × 10^-9^ | 5.00 × 10^-17^ | 4.60 × 10^-6^ |
|  | Li_2_ZrCl_5.5_I_0.5_ | 9.02 × 10^-6^ | 7.21 × 10^-6^ | 6.91 × 10^-6^ |
|  | Li_2_ZrCl_5_I | 1.18 × 10^-5^ | 3.40 × 10^-5^ | 2.00 × 10^-5^ |

**Table S21.** Summary of previous studies on anion-substituted halide SEs.

| Composition | | Ionic Conductivity (S cm^-1^) | | Space group | Ref |
| --- | --- | --- | --- | --- | --- |
| Before Substitution | After Substitution | Before Substitution | After Substitution |  |  |
| Li_2_ZrCl_6_ | Li_2_ZrCl_6-y_X_y_ (X = Br, I) | 4.0 × 10^-4^ | 9.0 × 10^-4^ | P$\bar{3}$m1 | This work |
| Li_3_ErCl_6_ | Li_3_ErI_6_ | 6.5 × 10^-4^ | 3.9 × 10^-4^ | P$\bar{3}$m1  🡪 C2/c | [S1] |
| Li_2_ZrCl_6_ | Li_3.2_ZrCl_4.8_S_1.2_ | 3.3 × 10^-4^ | 7.5 × 10^-5^ | P$\bar{3}$m1  🡪 C2/m | [S2] |
|  | Li_3.1_ZrCl_4.9_O_1.1_ |  | 1.3 × 10^-3^ | P$\bar{3}$m1  🡪 C2/m |  |
| Li_2_ZrCl_6_ | Li_2_ZrCl_5.5_F_0.5_ | 3.5 × 10^-4^ | 1.2 × 10^-4^ | P$\bar{3}$m1 | [S3] |
|  | Li_2.5_ZrCl_5.5_O_0.5_ |  | 1.39 × 10^-3^ | P$\bar{3}$m1  🡪 C2/m |  |
|  | Li_2.5_ZrCl_5_F_0.5_ O_0.5_ |  | 1.17 × 10^-3^ | P$\bar{3}$m1  🡪 C2/m |  |
| Li_2_ZrF_6_ | Li_2_ZrF_6-x_Cl_x_ | < 10^-10^ | 5.5 × 10^-7^ | P$\bar{3}$m1 | [S4] |
| Li_3_YCl_6_ | Li_3_YCl_3_Br_3_ | 5.1 × 10^-4^ | 1.6 × 10^-3^ | P$\bar{3}$m1  🡪 C2/m | [S5] |
| Li_3_YBr_6_ | Li_3_YBr_6-x_F_x_ | 2.1 × 10^-3^ | 1.8 × 10^-3^ | C2/m | [S6] |
| Li_3_HoCl_6_ | Li_3_HoCl_6-x_Br_x_ | 1.2 × 10^-4^ | 1.24 × 10^-4^ | P$\bar{3}$m1 | [S7] |
| Li_3_HoBr_6_ | Li_3_HoBr_6-x_I_x_ | 1.6 × 10^-3^ | 2.7 × 10^-3^ | C2/m | [S8] |
| Li_3_InCl_6_ | Li_3_InCl_6-x_F_x_ | 1.3 × 10^-3^ | 5.1 × 10^-4^ | C2/m | [S9] |

**Table S22.** Comparison of representative halide SEs.

| Composition | Metal Chloride Precursor | Cost (USD kg^-1^) [S10] | Ionic Conductivity (mS cm^-1^) | Activation energy | Voltage Range (V vs Li/Li^+^) | Ref |
| --- | --- | --- | --- | --- | --- | --- |
| Li_2_ZrCl_5.5_I_0.5_ | ZrCl_4_ | 12.5 | 0.91 | 0.35 | 3.0 – 4.3 | This work |
| Li_3_YCl_6_ | YCl_3_ | 320.3 | 0.51 | 0.40 | 2.52 − 4.22 | [S11] |
| Li_3_InCl_6_ | InCl_3_ | 595.5 | 1.49 | 0.326 | 2.5 − 4.2 | [S12] |
| Li_3_ScCl_6_ | ScCl_3_ | 17,400 | 3.0 | 0.36 | 2.52 − 4.22 | [S13] |
| Li_2.633_Er_0.633_Zr_0.367_Cl_6_ | ErCl_3_ | 2,200 | 1.1 | 0.37 | 3.0 – 4.3 | [S14] |
|  | ZrCl_4_ | 12.5 |  |  |  |  |
| Li_2.7_Yb_0.7_Zr_0.3_Cl_6_ | YbCl_3_ | 6,000 | 1.1 | 0.30 | 3.0 – 4.3 | [S15] |
|  | ZrCl_4_ | 12.5 |  |  |  |  |
| Li_2_In_0.33_Sc_0.33_Cl_4_ | InCl_3_ | 595.5 | 2.0 | 0.33 | 2.8 − 4.3 | [S16] |
|  | ScCl_3_ | 17,400 |  |  |  |  |

**4. References**

[S1] R. Schlem, T. Bernges, C. Li, M. A. Kraft, N. Minafra, W. G. Zeier, *ACS Applied Energy Materials* **2020**, 3, 3684.

[S2] K.-H. Park, S. Y. Kim, M. Jung, S.-B. Lee, M.-J. Kim, I.-J. Yang, J.-H. Hwang, W. Cho, G. Chen, K. Kim, J. Yu, *ACS Applied Materials & Interfaces* **2023**, 15, 58367.

[S3] L. Shen, J.-L. Li, W.-J. Kong, C.-X. Bi, P. Xu, X.-Y. Huang, W.-Z. Huang, F. Fu, Y.-C. Le, C.-Z. Zhao, H. Yuan, J.-Q. Huang, Q. Zhang, *Advanced Functional Materials* **2024**, 34, 2408571.

[S4] E. Umeshbabu, S. Maddukuri, Y. Hu, M. Fichtner, A. R. Munnangi, *ACS Applied Materials & Interfaces* **2022**, 14, 25448.

[S5] Z. Liu, S. Ma, J. Liu, S. Xiong, Y. Ma, H. Chen, *ACS Energy Letters* **2021**, 6, 298.

[S6] T. Yu, J. Liang, L. Luo, L. Wang, F. Zhao, G. Xu, X. Bai, R. Yang, S. Zhao, J. Wang, J. Yu, X. Sun, *Advanced Energy Materials* **2021**, 11, 2101915.

[S7] J. Peng, L. Xian, L.-B. Kong, *Ionics* **2023**, 29, 2657.

[S8] M. A. Plass, S. Bette, R. E. Dinnebier, B. V. Lotsch, *Chemistry of Materials* **2022**, 34, 3227.

[S9] S. Zhang, F. Zhao, S. Wang, J. Liang, J. Wang, C. Wang, H. Zhang, K. Adair, W. Li, M. Li, H. Duan, Y. Zhao, R. Yu, R. Li, H. Huang, L. Zhang, S. Zhao, S. Lu, T.-K. Sham, Y. Mo, X. Sun, *Advanced Energy Materials* **2021**, 11, 2100836.

[S10] K. Wang, Q. Ren, Z. Gu, C. Duan, J. Wang, F. Zhu, Y. Fu, J. Hao, J. Zhu, L. He, C.-W. Wang, Y. Lu, J. Ma, C. Ma, *Nature Communications* **2021**, 12, 4410.

[S11] T. Asano, A. Sakai, S. Ouchi, M. Sakaida, A. Miyazaki, S. Hasegawa, *Advanced Materials* **2018**, 30, 1803075.

[S12] X. Li, J. Liang, J. Luo, M. Norouzi Banis, C. Wang, W. Li, S. Deng, C. Yu, F. Zhao, Y. Hu, T.-K. Sham, L. Zhang, S. Zhao, S. Lu, H. Huang, R. Li, K. R. Adair, X. Sun, *Energy & Environmental Science* **2019**, 12, 2665.

[S13] J. Liang, X. Li, S. Wang, K. R. Adair, W. Li, Y. Zhao, C. Wang, Y. Hu, L. Zhang, S. Zhao, S. Lu, H. Huang, R. Li, Y. Mo, X. Sun, *Journal of the American Chemical Society* **2020**, 142, 7012.

[S14] K.-H. Park, K. Kaup, A. Assoud, Q. Zhang, X. Wu, L. F. Nazar, *ACS Energy Letters* **2020**, 5, 533.

[S15] S. Y. Kim, K. Kaup, K.-H. Park, A. Assoud, L. Zhou, J. Liu, X. Wu, L. F. Nazar, *ACS Materials Letters* **2021**, 3, 930.

[S16] L. Zhou, T.-T. Zuo, C. Y. Kwok, S. Y. Kim, A. Assoud, Q. Zhang, J. Janek, L. F. Nazar, *Nature Energy* **2022**, 7, 83.
